# Discovery of Novel c-di-GMP-Related Genes in *Leptospira interrogans*

**DOI:** 10.3390/pathogens15020151

**Published:** 2026-01-30

**Authors:** Anielle Salviano de Almeida Ferrari, Davi Gabriel Salustiano Merighi, Aline Biazola Visnardi, Gabriela Roberto Silva, Cauê Augusto Boneto Gonçalves, Daniel Enrique Sanchez-Limache, Bruna Sayuri Cardoso Ogusku, Anacleto Silva de Souza, Robson Francisco de Souza, Cristiane Rodrigues Guzzo

**Affiliations:** Department of Microbiology, Institute of Biomedical Sciences, University of São Paulo, São Paulo 05508-000, Brazil; anielle.ferrari@usp.br (A.S.d.A.F.); davism@usp.br (D.G.S.M.); alinebiazola@usp.br (A.B.V.); gabrielarobertosilva@usp.br (G.R.S.); caue.boneto@usp.br (C.A.B.G.); daniel_sanchez@usp.br (D.E.S.-L.); bruna.ogusku@usp.br (B.S.C.O.); anacletosilvadesouza@usp.br (A.S.d.S.); rfsouza@usp.br (R.F.d.S.)

**Keywords:** GGDEF, PilZ, EAL, HD-GYP, c-di-GMP receptors

## Abstract

Cyclic di-GMP (bis-(3′→5′) cyclic dimeric guanosine monophosphate) is a ubiquitous bacterial second messenger that regulates a wide range of cellular processes, including biofilm formation, motility, virulence, and environmental adaptation. Its intracellular levels are dynamically controlled by diguanylate cyclases (DGCs), which synthesize c-di-GMP from GTP, and phosphodiesterases (PDEs), which degrade it into linear pGpG or GMP. The functional effects of cytoplasmic c-di-GMP are mediated through diverse effector proteins, including PilZ domain-containing receptors, transcription factors, and riboswitches. In *Leptospira interrogans*, a major pathogenic species responsible for leptospirosis, the regulatory roles of c-di-GMP remain poorly understood. Here, we performed a comprehensive bioinformatics and structural analysis of all predicted c-di-GMP related proteins in *L. interrogans* serovar Copenhageni strain Fiocruz L1-130, a serovar generally associated with severe manifestations of leptospirosis in humans. Our analysis identified seventeen proteins containing GGDEF domain, five proteins containing both GGDEF and EAL domains, four proteins containing EAL domain, five proteins containing HD-GYP domain, twelve proteins containing PilZ domain, and one protein containing an MshEN domain. Comparative analysis with well-characterized bacterial homologs suggests that *L. interrogans* possess a complex c-di-GMP signaling network, likely involved in modulating biofilm formation, host–pathogen interactions, and environmental survival. These findings provide new insights into the c-di-GMP regulatory network and on signal transduction in *Leptospira* and lay the foundation for future functional studies aimed at understanding its roles in physiology, virulence, and persistence.

## 1. Introduction

Leptospirosis is a globally re-emerging zoonotic disease caused by pathogenic bacteria of the genus *Leptospira*, which infect mainly mammals, including humans [1]. *Leptospira interrogans* is the predominant pathogenic species within the genus, responsible for most human leptospirosis cases and the most severe clinical manifestations of the disease [2,3,4,5]. Leptospirosis symptoms are nonspecific and often mimic other tropical diseases such as dengue, influenza, malaria, rickettsiosis, acute Chagas disease, toxoplasmosis, COVID-19 (Coronavirus Disease 2019), and typhoid fever [6]. The clinical presentation varies widely, ranging from asymptomatic or mild cases—characterized by fever, myalgia (particularly calf pain), headache, nausea/vomiting, diarrhea, arthralgia, conjunctival redness, photophobia, and occasionally rash—to severe manifestations like Weil’s syndrome, which involves jaundice, acute kidney injury, hepatic dysfunction, meningitis, pulmonary hemorrhage, Acute Respiratory Distress Syndrome (ARDS), hypotension, and potentially fatal multi-organ failure [6,7,8].

Leptospirosis is more prevalent in tropical regions, where warm climates and heavy rainfall create favorable conditions for its transmission. However, leptospirosis is also re-emerging in Europe and other countries in the Northern Hemisphere [9,10]. These spirochetes are maintained in nature through chronic renal colonization of asymptomatic reservoir hosts, particularly rodents, which continuously excrete viable bacteria into the environment via urine. Upon release, *Leptospira* can persist in moist soil and aquatic environments for several weeks and, under certain conditions, may exhibit limited replication. Environmental transmission plays a central role in the epidemiology of leptospirosis, with outbreaks frequently associated with heavy rainfall, flooding, and other extreme weather events that increase human exposure to contaminated water [11]. Climate change has intensified these conditions, with rising temperatures and increased rainfall frequency and intensity—especially in tropical areas—further facilitating the spread of the bacteria. An example of this trend occurred in Rio Grande do Sul (Brazil) in 2024, where extreme flooding affected approximately 96% of the state’s municipalities and displaced approximately 600,000 people. That year, the state recorded 1313 cases of leptospirosis, resulting in 53 deaths—a 2.7-fold increase in infections and doubling of the number of fatalities compared to 2023. These deaths accounted for approximately 14% of all fatalities linked to the 2024 flooding and disease outbreak in Rio Grande do Sul [12,13,14,15].

The adaptation mechanisms used by *Leptospira* when exposed to the environment remain poorly understood. Recent efforts have focused on elucidating the biological and environmental factors that contribute to the survival, dissemination, and infectivity of these pathogens outside the host [11,16,17,18,19,20]. Davignon and co-authors performed an RNA-seq analysis comparing 21-day-old biofilms (mature biofilm phase) with 5-day-old exponential cultures (planktonic phase) and showed significant modulation of the expression of c-di-GMP related genes in the two growth conditions, clarifying the importance of c-di-GMP regulation and signaling in *L. interrogans* biofilm [11]. Moreover, the mature biofilm phase exhibited downregulation of motility, energy production, and metabolic pathways, while genes associated with protein quality control, redox balance, and defense against metal stress were strongly induced suggesting a copper-responsive stress adaptation. Despite these physiological changes, virulence assays in a hamster model (*Mesocricetus auratus*) confirmed that *L. interrogans* cells retained their pathogenic potential in the mature biofilm and in the planktonic phases, indicating that biofilm-associated adaptations do not compromise infectivity [11]. In addition, an increase in c-di-GMP triggers biofilm formation in *L. interrogans* and promotes increased survival under environmental stress [16]. Xiao and collaborators showed that the shift from a lower to a higher temperature resulted in low c-di-GMP concentration in *L. interrogans* and most of the c-di-GMP metabolic genes exhibited differential temperature regulation. This was the first work to demonstrate the relevance of c-di-GMP networks in the environmental adaptation for this bacterium. Furthermore, infection of murine J774A.1 macrophage-like cells led to a reduction in intracellular c-di-GMP levels, despite the absence of significant transcriptional changes in genes involved in c-di-GMP metabolism during the course of infection [18]. Such results suggest changes in c-di-GMP levels probably allows this bacteria to better adapt to different complex microenvironments, such as in the environment and infecting mammalian hosts [11,16,18]. In bacteria, c-di-GMP is a ubiquitous second messenger that regulates diverse cellular processes [21,22]. In general, elevated c-di-GMP intracellular concentrations promote biofilm formation through extracellular matrix production, while low levels enhance motility via flagellar activation [23,24]. Such precise control of bacterial behavior makes c-di-GMP signaling crucial for environmental adaptation and pathogenicity [19,22].

**Figure 1:**
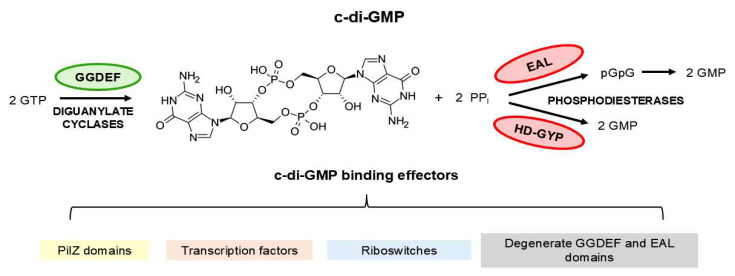
Synthesis and degradation of c-di-GMP. The synthesis of c-di-GMP is catalyzed by diguanylate cyclases (DGCs) through the cooperative action of their catalytic GGDEF domains (green), which promote the conversion of two GTP molecules into one c-di-GMP molecule and two pyrophosphate molecules. The degradation of c-di-GMP is carried out by specific phosphodiesterases (PDEs) containing EAL or HD-GYP domains (red). These enzymes hydrolyze c-di-GMP into 5’-phosphoguanylyl-(3′-5′)-guanosine (pGpG) or two molecules of guanosine monophosphate (GMP), respectively. The regulation of cellular processes mediated by c-di-GMP occurs through the interaction of this molecule with various effectors, including proteins containing PilZ domains, transcription factors, riboswitches, and degenerate GGDEF or EAL domains. In this regard, c-di-GMP influences essential processes such as motility, adhesion, biofilm formation, virulence, and other bacterial behaviors.

The c-di-GMP regulatory network operates through two main components: diguanylate cyclases (DGCs) containing GGDEF domains that synthesize c-di-GMP from two molecules of GTP, and phosphodiesterases (PDEs) like EAL or HD-GYP domains that degrade c-di-GMP to pGpG or GMP, respectively [19,22,25,26] (Figure 1). These enzymes work with diverse effectors that translate c-di-GMP signals into cellular responses, such as riboswitches, PilZ proteins, and other c-di-GMP binding macromolecules present in transcription factors, enzymes, and other targets [20,22,26,27]. The PilZ domain was the first protein domain identified as a c-di-GMP receptor and remains the most well-characterized to date [19,20,22]. It can play key roles in coordinating the transition to biofilm formation and the expression of virulence factors. In pathogens such as *Pseudomonas aeruginosa*, the production of c-di-GMP controls virulence factor expressions and antibiotic resistance mechanisms [28] highlighting its clinical relevance. Previous work by Xiao and coauthors systematically analyzed the c-di-GMP metabolic enzymes of *L. interrogans*, identifying and experimentally characterizing several proteins with GGDEF, EAL, and HD-GYP domains. A comprehensive genome-wide analysis that also includes c-di-GMP receptors such as PilZ and MshEN domain-containing proteins will expand the catalog of potential c-di-GMP receptors and signaling components in *L. interrogans*.

Herein, we present a comprehensive bioinformatic and structural characterization of the c-di-GMP signaling network in *L. interrogans* serovar Copenhageni strain Fiocruz L1-130. Through systematic analysis of protein domains and comparative genomics, we identified: 17 GGDEF-containing proteins (putative DGCs), four EAL, and five HD-GYP domain proteins (putative PDEs), five GGDEF-EAL containing both domains, and 13 potential receptors (12 proteins containing PilZ domains and one protein containing an MshEN domain). This study provides a systematic and comprehensive characterization of the c-di-GMP signaling repertoire in *L. interrogans*, establishing a molecular framework for future mechanistic studies of c-di-GMP-mediated regulatory networks in this neglected zoonotic human pathogen.

## 2. Materials and Methods

### 2.1. Potential Proteins Involved in c-di-GMP Signaling in L. interrogans: Bioinformatic Analysis and Structural Prediction Models

To identify proteins associated with c-di-GMP signaling in the *Leptospira* genus, a systematic search was conducted between February and March 2024. Searches were performed using the UniProt database 2 [29], employing relevant keywords (Table S1) associated with c-di-GMP signaling domains, including GGDEF, EAL, HD-GYP, PilZ, and MshEN, in combination with the term “*Leptospira*”. This yielded 4,913 protein sequences (Supplementary File S1) that potentially contain domains associated with c-di-GMP signaling. Additionally, complementary searches were conducted in the NCBI c-di-GMP database [22,30,31,32,33] to identify genes related to c-di-GMP signaling that had already been characterized or annotated within the *Leptospira* genus. Following the construction of the database, a BLASTp search [34] was performed using the genome of *L. interrogans* serovar Copenhageni strain Fiocruz L1-130 (Lic) (Taxonomy ID: 267671) [35] as the query. The search was conducted against the identified proteins, with an *e*-value threshold ≤ 10^−5^ and sequence identity ≥ 40%. Based on the database of potential proteins identified in Lic containing PilZ, MshEN, GGDEF, EAL, and/or HD-GYP domains, three-dimensional structure predictions were performed for all proteins using AlphaFold (version 3) [36]. Subsequently, the predicted structures were evaluated for their association with c-di-GMP signaling proteins by identifying homologs through searches conducted on the FoldSeek and DALI servers [37,38,39].

Our analysis combined sequence-based and structural bioinformatics approaches to identify proteins containing domains related to c-di-GMP signaling. We first searched the UniProt database [29] using keywords for c-di-GMP-related domains combined with the term “*Leptospira*” to build a preliminary database. This was complemented by searches in the NCBI c-di-GMP database [22,30,31,32,33] to identify previously annotated genes in the *Leptospira* genus. Following database construction, a BLASTp [34] analysis was performed using the Lic genome as the query, as some annotations were present in other *Leptospira* species but absent in Lic. To verify the presence of these genes in Lic, BLASTp searches [34] were conducted, and the corresponding domains were further validated through the structural analysis described below. To validate the identified proteins, three-dimensional structures were predicted with AlphaFold 3 [36] and structural models were evaluated using per-residue pLDDT scores, considering regions with pLDDT values above 70 as reliably predicted. These structures were analyzed using the FoldSeek and DALI servers [37,38,39]. Structural similarities were assessed using DALI Z-scores greater than 5.0, and all hits were manually inspected for topological conservation and modelled regions with pLDDT values above 70.

We also performed domain analysis using the Conserved Domain Database (CDD) [40], InterPro [41], and Pfam [42]. Unlike methodologies previously applied to *Leptospira* [16,18,19,20], our approach is not restricted to primary sequence analysis (e.g., BLAST and its variants, HMMs) and automatic annotation (e.g., PGAP—NCBI Prokaryotic Genome Annotation Pipeline, Prokka, Bakta, etc.). Instead, it integrates validation through structural similarity and iterative BLAST searches across different *Leptospira* species. By incorporating this critical layer of structural validation and homology analysis, we provide a more robust identification of c-di-GMP-signaling proteins.

### 2.2. Functional and Structural Characterization of Identified Proteins: An Analytical Approach

The prediction of the protein subcellular localization was performed using CELLO (version 2.5), while the presence of signal peptides was predicted using SignalP (version 5) or Signal-3L (version 3), and the prediction of the presence of transmembrane domains were predicted using TMHMM (version 2.0) or DeepTMHMM (version 1.0) [43,44,45,46,47]. Three-dimensional structural models were generated using AlphaFold version 3 [36] or obtained from the AlphaFold DB (version 2022-05-31) available through the UniProt server [29].

### 2.3. Multiple Amino Acid Sequence Alignment Using Three-Dimensional Structure Predictions

Multiple sequence alignments (MSAs) were performed using the predicted tertiary structures of each domain generated by the AlphaFold3 [36] as an input for the DALI server [38] to obtain structure-based alignments. MSAs were visualized and curated using Jalview program (version 2.11.2.7) [48]. Each domain was compared to well-characterized reference proteins to infer its potential functional characteristics. The GGDEF domain was compared to PleD (*locus_tag* CCNA_02546, PDB 1W25 [49]) from *Caulobacter vibrioides* (synonym *C. crescentus*, Taxonomy ID: 565050) [50]. The EAL domain was compared to the RocR (*locus_tag* PA3947, PDB 3SY8 [51]) from *P. aeruginosa* (Taxonomy ID: 208964) [52], and the HD-GYP domain was referenced to PmGH (*locus_tag* PERMA_0986, PDB 4MDZ [53]) from *Persephonella maritima* (Taxonomy ID: 123214) [54]. For the PilZ domain, the comparison was performed with MapZ (*locus_tag* PA4608, PDB 5XLY [55]) from *P. aeruginosa*, and the MshEN domain was compared with VcMshEN (*locus_tag* VC_0405) [32] from *Vibrio cholerae* (Taxonomy ID: 243277) [56].

### 2.4. Pangenoma Analysis of Leptospira interrogans Isolates

Complete genome sequences of different *L. interrogans* isolates were obtained from the NCBI’s RefSeq database (release 232) [57]. Using the metadata and assembly statistics table from the Genome Taxonomy Database (version 226) [58], *L. interrogans* genomes were selected based on CheckM2 completeness ≥ 95% and contamination ≤ 5% [59]. For all genomes matching these criteria, the gene annotations provided by RefSeq were used to extract all protein sequences using the rgaparser Python script from the Rotifer’ comparative genomics library (version 0.1, executed by Python version 3.10) [60]. The PanACoTA pangenomics toolkit (version 1.4.0) [61] was used to identify orthologous gene families. A phylogenetic tree based on the complete genomes of all *L. interrogans* isolates was built using the all-versus-all sequence similarity matrix of their nucleotide sequences generated by FastANI (version 1.34) [62] using the Neighbor-Joining (NJ) method implemented in the Bio.Phylo module of the BioPython library (version 1.81) [63].

### 2.5. Presence of the Genes Found in L. interrogans Serovar Copenhageni strain Fiocruz L1-130 in Other Leptospira Genomes

After identifying the genes related to c-di-GMP signaling in *L. interrogans* serovar Copenhageni strain Fiocruz L1-130, we performed BLASTp [34] searches against three other *Leptospira* genomes to assess the distribution and conservation of orthologous genes. Percentages of sequence similarity and identity of homologous genes were compared to those of *L. interrogans* serovar Copenhageni strain Fiocruz L1-130. The genomes used were: (1) *L. interrogans* serovar Lai strain 56601 (Taxonomy ID: 189518) [64], to evaluate the degree of gene conservation within a representative of the same species; (2) *L. borgpetersenii* serovar Hardjo-bovis strain JB197 (Taxonomy ID: 355277) [65], used as a representative of a different pathogenic species. This species shows genome reduction compared to *L. interrogans* and is transmitted strictly through host-to-host contact [65,66,67]; and (3) *L. biflexa* serovar Patoc strain Patoc 1 (Paris) (Taxonomy ID: 456481) [68], as a saprophytic specie.

### 2.6. Graphical, Structural, and Imaging Software Tools

The graphs presented in this study were generated using the R software (version 4.4.1) [69] employing the packages BiocManager [70], ComplexHeatmap [71], and ape (version 5.0) [72]. The images were constructed using the Inkscape program (version 1.3.2) [73], while protein visualization and editing were performed using the PyMOL software (version 3.0, Schrödinger, LLC, New York, NY, USA) [74] or ChimeraX (version 1.18) [75].

## 3. Results

### 3.1. Identification of c-di-GMP-Related Genes in L. interrogans

**Figure 2:**
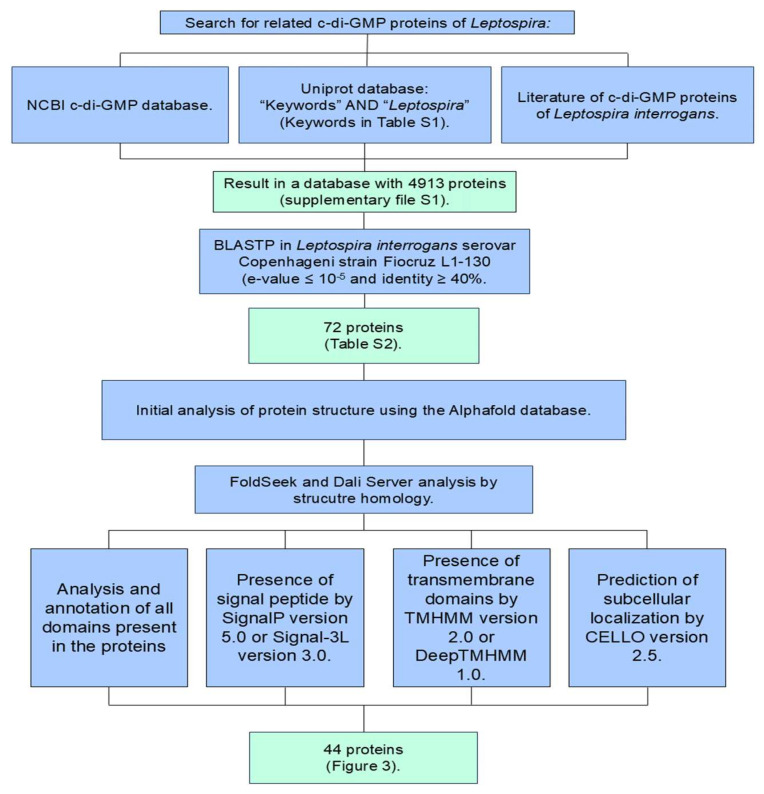
Workflow of the bioinformatics analysis of c-di-GMP-related genes. The database was built by searching the NCBI c-di-GMP database, UniProt, and the literature. The identified proteins were submitted to BLASTp analysis, yielding 72 candidate proteins potentially associated with c-di-GMP signaling (see Table S2 in Supplementary Materials). These proteins were subsequently analyzed using AlphaFold3 for structural prediction and FoldSeek for structural homology assessment. Protein domains were identified and annotated, alongside the analysis of signal peptides, transmembrane regions, and predicted subcellular localization, resulting in the identification of 44 proteins (Figure 3).

**Figure 3:**
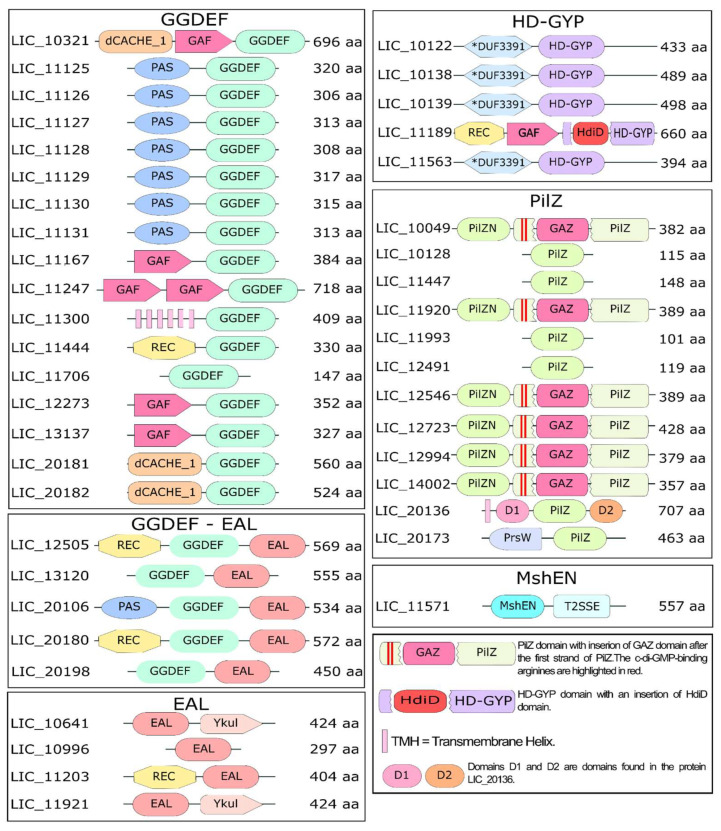
Proteins identified in the genome of *L. interrogans* serovar Copenhageni strain Fiocruz L1-130 containing GGDEF, EAL, PilZ, HD-GYP, and MshEN domains. Domain architecture of c-di-GMP-related proteins in Lic is shown. Protein names are listed on the left, with the corresponding number of amino acids on the right. The protein sequence analysis, domain identification, transmembrane domain prediction (TMH), signal peptide detection were conducted using UniProt, CDD, DeepTMHMM 1.0, and SignalIP-5.0. The genes LIC_10049, LIC_11920, LIC_12546, LIC_12723, LIC_12994, LIC_14002 (not annotated in *L. interrogans* serovar Copenhageni strain Fiocruz L1-130 genome; the locus_tag corresponds to RefSeqGene LIC_14002 or LIC_RS11585, and this gene is orthologous to LA_1489) were identified. The gene with locus_tag LIC_13137 was experimentally characterized and named Lcd1 protein [17]. The paralog group, including LIC_10049, LIC_11920, LIC_12546, LIC_12723, LIC_12994, and LIC_14002, was recognized as members of the YcgR^GAZ^ family [20]. Additionally, LIC_11571 is annotated as GspE, associated with the type II secretion system (T2SS). DeepTMHMM 1.0 shows that most regions of the proteins are predicted to be cytoplasmic, except for the transmembrane domains, which span the membrane. Transmembrane helices (TMH) are shown as light pink bars present in LIC_11300 and LIC_20136. LIC_20173 contains an N-terminal PrsW domain composed of nine transmembrane helices: seven within the PrsW core and two additional helices preceding the PilZ domain (see Table S3). The N-terminal regions of LIC_20173 and LIC_20136 probably are oriented toward the periplasm, likely inserted into the inner membrane by the YidC insertase, which follows the positive-inside rule. In all cases, the GGDEF domain of LIC_11300 and the PilZ domains of LIC_20136 and LIC_20173 are predicted to be located in the cytoplasm. *Possible DUF3391: The N-terminal regions of LIC_10122, LIC_10138, and LIC_10139 likely correspond to DUF3391 (PF11871) domains, although only LIC_11563 is correctly annotated in prediction databases. CDD and InterPro did not detect DUF3391 in the other three proteins, but sequence length and AlphaFold models support structural similarity. All four proteins display globular folds with 4–6 antiparallel β-strands and a C-terminal α-helix, with subtle differences in α-helix positioning (Figure S6A).

To elucidate the c-di-GMP signaling network in *L. interrogans* serovar Copenhageni strain Fiocruz L1-130 (Lic), we conducted a systematic search of the UniProt database using domain-specific keywords (GGDEF, EAL, HD-GYP, PilZ, and MshEN) alongside the term “*Leptospira*” in order to identify candidate proteins potentially involved in c-di-GMP signaling (Supplementary File S1). This search yielded 4913 protein sequences. Complementary searches in the NCBI c-di-GMP database did not yield additional annotated proteins within the Leptospira genus, suggesting our initial dataset was comprehensive. We then performed a BLASTp analysis of these candidates against the Lic genome, applying stringent thresholds (*e*-value ≤ 10^−5^, identity ≥ 40%). These conservative cutoffs were selected to ensure high-confidence assignments, although we acknowledge that such parameters may miss highly divergent homologs, thereby limiting sensitivity. Using this approach, we identified 72 potential homologs (Table S2). Domain validation was achieved through a combination of AlphaFold-predicted structures and FoldSeek-based structural analysis, followed by manual inspection. Through our comprehensive bioinformatics approach (Supplementary File S1, Figure 2, Tables S1 and S2), we identified and confirmed the presence of at least 44 proteins in the *Lic* genome that contain the target domains associated with c-di-GMP signaling (Figure 3). Based on these results, we identified previously unrecognized proteins containing c-di-GMP–related domains that had not been described before: two protein containing PilZ domain (LIC_11993 and LIC_20136), four with GGDEF domains (LIC_10321, LIC_11167, LIC_11247, and LIC_11706), one with HD-GYP domain (LIC_10122), and two proteins containing EAL domains, which were not previously described as having also a GGDEF domain (LIC_20106 and LIC_20198).

Interestingly, LIC_11993 is annotated as a PilZ-domain-containing protein in the UniProt database and as a PilZ domain protein in the KEGG database (*e*-value = 0.0037). Previous results using structural similarities by the DALI server using the structural model of LIC_11993 created by the Alfafold from the UniProt database did not detect proteins containing the PilZ domain using the DALI server analysis. However, manual inspection of its AlphaFold version 3 predicted structure revealed structural similarity to PilZ domain proteins when analyzed using the DALI server (z-score of 7.3 for the PilZ domain of VCA0042, PDB: 3KYG) [20]. Therefore, LIC_11993 is a protein with a divergent PilZ domain. Another protein, LIC_11628, is also annotated in UniProt as a PilZ-domain–containing protein. Nevertheless, manual analysis based on AlphaFold3 predictions and structural similarity searches using both FoldSeek and DALI servers failed to identify any significant structural similarities to canonical PilZ domain structures. In our analysis, we also checked whether the start codons of the genes were correctly annotated, and we identified some misannotations (Table S5). For example, the gene LIC_12273 is missing the first 40 residues in the KEGG and NCBI databases, while it is correctly annotated in the RefSeq database.

The proteins were classified by domain composition: 17 contained GGDEF domains, four possessed EAL domains, five contained both GGDEF and EAL domains, five harbored HD-GYP domains, 12 featured PilZ domains, and one carried an MshEN domain. Structural analysis using AlphaFold, FoldSeek, and the DALI server revealed conserved folds and potential functional diversification. Further characterization via UniProt, CDD, DeepTMHMM, and SignalP-5.0 provided insights into domain organization, transmembrane topology, and secretion signals. Proteins are predicted to be primarily cytoplasmic, with the exception for their transmembrane segments, which span the membrane. In particular, our analysis suggests that the GGDEF domain of LIC_11300 and the PilZ domains of LIC_20136 and LIC_20173 are located in the cytoplasm. The predicted orientation of these transmembrane regions is indicated in the legend of Figure 3 and described in more detail below. These results significantly expand the catalog of c-di-GMP-related proteins in *L. interrogans*, highlighting previously unrecognized components. The integration of structural prediction and multi-database annotation offers a robust framework for future experimental studies targeting specific domains or proteins.

The comparative analysis revealed that most of the c-di-GMP-signaling-related genes identified in *L. interrogans* serovar Copenhageni strain Fiocruz L1-130 are conserved in *L. interrogans* serovar Lai strain 56601 (Taxonomy ID: 189518) [64], showing high sequence identity. In *L. borgpetersenii* serovar Hardjo-bovis strain JB197 (Taxonomy ID: 355277) [65], several orthologs were also detected, although some genes appear to be absent, consistent with the known genome reduction in this species [65,66,67]. In contrast, only a limited number of homologous sequences were found in *L. biflexa* serovar Patoc str. Patoc 1 (Paris) (Taxonomy ID: 456481) [68], reflecting the genetic and ecological differences between pathogenic and saprophytic *Leptospira*. Detailed BLASTp results are provided in Table S6.

### 3.2. Diversity of Sensor Domains Present in c-di-GMP-Related Proteins

Proteins involved in c-di-GMP signaling generally respond to diverse environmental and intracellular stimuli, modulating their activity to promote survival through behavioral or physiological adaptations [26]. To ensure rapid and efficient responses, sensor or signaling domains are frequently associated with c-di-GMP-related proteins, regulating enzymatic or receptor functions via signal transduction pathways [76]. While various signal transduction domains are associated with c-di-GMP signaling proteins, the occurrence of c-di-GMP-related genes without sensor domains is rare; nonetheless, these genes may remain functional [77]. To define the sensor domains associated with c-di-GMP-related proteins in Lic, we generated structural models of these domains separately using AlphaFold3. The resulting structures were analyzed using FoldSeek, DALI server, or HHpred in order to characterize the domain or infer its function based on three-dimensional structural homology. The identified sensor and signaling domains present in *Lic* are summarized in Table S3.

### 3.3. Diguanylate Cyclases

The GGDEF domain is a highly conserved bacterial enzyme that functions as a diguanylate cyclase enzyme (DGC), catalyzing the synthesis of the secondary messenger c-di-GMP from two guanosine-5′-triphosphate (GTP) molecules, via a bi-ter reaction yielding one c-di-GMP and two pyrophosphates. Catalysis requires dimerization to form an active site where the GG(D/E)EF motif (comprising Gly-Gly(Asp/Glu)-Glu-Phe) creates an active site that enables nucleophilic attack on the α-phosphate of each GTP substrate. Structurally, the domain adopts an α/β fold (α0-β1-α1-α2-β2-β3-α3-β4-β5-β6-α4-β7 topology) with five central β-strands flanked by five α-helices [17,19,22,26]. The GG(D/E)EF motif is located in the loop between β2 and β3 of the domain. The glutamic acid residue in this motif plays a key role in catalysis by binding the α-phosphate group of the GTP molecule and coordinating one of the cations in the binding site. In the case of the PleD GGDEF domain of *C. crescentus*, the catalytic site accommodates two magnesium cations, which are coordinated by D327, the backbone of I328 and E370 of the GG(D/E)EF motif. These metal ions are essential for stabilizing the negative charges on the phosphate groups during the catalysis process. The N335 and D344 residues are involved in binding the guanine base of the GTP substrate, contributing to substrate specificity. Meanwhile, the side chains of L294, L347, E370, K442, R446, and the backbone residues of F330, F331, and K332 interact with the phosphate groups of the GTP molecule, further stabilizing the transition state to form the c-di-GMP product [26,78,79].

GGDEF domain activity is regulated through two primary mechanisms: (1) upstream sensor domains that often mediate dimerization, and (2) allosteric inhibition by c-di-GMP binding. The dimeric enzyme contains two symmetrical allosteric sites (I and I’), each composed of four critical residues: the RxxD motif (R359/D362) and R390 from one monomer [26,78]. The fourth residue comes from the adjacent GGDEF monomer (e.g., R313 in PleD) [26,78]. When c-di-GMP binds to these sites, it crosslinks and immobilizes the GGDEF dimer in an inactive conformation. This noncompetitive product inhibition prevents catalysis, thereby controlling cellular c-di-GMP levels. A subclass of GGDEF enzymes, referred to as Hybrid promiscuous (Hypr) GGDEF enzymes, exhibits broader substrate specificity. These enzymes predominantly produce cGAMP (cyclic GMP-AMP), but they can also generate c-di-AMP and c-di-GMP depending on active site modifications [80,81,82]. The ability to synthesize multiple cyclic nucleotides is attributed to a specific residue substitution: in the case of the PleD protein, the aspartate residue at position D344 (located within the α2 helix) is replaced by a serine, which affects the enzyme’s substrate preference by altering how the guanine base of GTP is recognized and bound [80,81,82].. Some GGDEF domains have become catalytically degenerate while acquiring novel functions: a protein from *C. crescentus* (*locus_tag* CC_3396) acts as a GTP sensor that allosterically activates an adjacent EAL domain’s phosphodiesterase activity, while *B. subtilis* YybT has evolved ATPase activity, completely diverging from c-di-GMP synthesis. Moreover, the YybT that has a PAS-GGDEF-DHH-DHHA1 domain architecture also cleaves c-di-AMP and c-di-GMP by the DHH-DHHA1 domains [83]. These examples demonstrate the remarkable functional plasticity of GGDEF domains in bacterial signaling networks [22,83].

**Figure 4:**
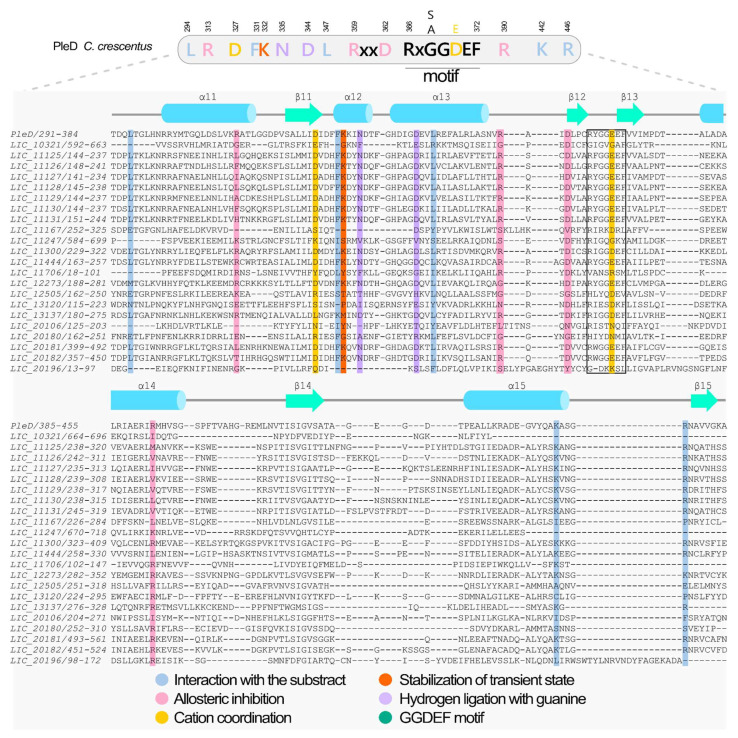
Characterization of the GGDEF domain of different proteins from *L. interrogans* serovar Copenhageni strain Fiocruz L1-130. Structural alignments of GGDEF domains from *L. interrogans* serovar Copenhageni strain Fiocruz L1-130 via the DALI server, using the PleD protein (*locus_tag* CCNA_02546, PDB 1W25 [49]) of *C. crescentus* as a reference. The catalytic mechanism was also inferred from the study published by Paul Wassmann and collaborators [85].

Our structural and functional analysis of the 22 putative diguanylate cyclases identified in Lic revealed 17 proteins containing only the GGDEF domain and five proteins with a tandem GGDEF-EAL domain architecture (Figure 3). Using AlphaFold, we predicted the protein structures and isolated the GGDEF domains using PyMOL for further analysis (as described in Section 2). Structural alignment against the reference GGDEF domain from *C. crescentus* PleD, via the DALI server [38], demonstrated the conservation of key catalytic residues, including the GG(D/E)EF motif (positions 368–372 in PleD), metal-coordinating residues (D327, E370), substrate-binding residues (L294, F331, N335, D344, L347, K442, and R446), and the transition-state stabilizer (K332) [84]. The allosteric regulation sites (RxxD motif, R390, R313) were also preserved in most proteins, though some showed variations suggesting functional divergence. Figure 4 highlights these structural alignments, emphasizing both the conserved features and unique adaptations among Lic GGDEF domains compared to PleD. This comprehensive analysis provides insights into the potential catalytic activity and regulatory mechanisms of these proteins in *L. interrogans*. The residue numbers are from PleD protein from *C. crescentus*. Figure 4 illustrates the GGDEF domain alignment, highlighting conserved residues essential for catalytic activity, relative to the reference protein PleD.

Based on this analysis, 13 out of the 22 proteins containing the GGDEF domain are possibly functional, as they retain the key residues essential for enzymatic catalytic activity. This includes the enzymes within the PAS-GGDEF cluster (LIC_11125, LIC_11126, LIC_11127, LIC_11128, LIC_11129, LIC_11130, and LIC_11131), as well as LIC_11300, LIC_11444, LIC_12273, LIC_13137^Lcd1^, LIC_20181, and LIC_20182 (Figure 4). Absence of these catalytic residues would render these proteins to be considered degenerated. Notably, the remaining proteins, including LIC_10321, LIC_11167, LIC_11247, LIC_11706, and all proteins containing both GGDEF and EAL domains, LIC_12505, LIC_13120, LIC_20106, LIC_20180, and LIC_20196, lack the residues necessary for catalytic activity and are therefore probably unable to synthesize c-di-GMP.

Analysis of the allosteric site revealed that the consensus sequence RxxD and a conserved arginine residue equivalent to PelD R390 are present in most of the canonical GGDEF proteins from Lic, with exception of LIC_12273 that has a degenerated allosteric site. Lic proteins with both symmetrical allosteric sites (I and I’ sites) conserved are LIC_11125, LIC_11126, LIC_11300, LIC_11444, LIC_11706, and LIC_13137^Lcd1^. Based on this analysis, 12 out of the 13 potentially active DGCs are likely to be allosterically regulated. Among the proteins containing the GGDEF domain and predicted to be inactive, we analyzed the presence of the allosteric site to assess their potential as c-di-GMP receptors rather than DGCs. Notably, LIC_11706 retained a conserved allosteric site, suggesting a possible role as a c-di-GMP receptor (Figure 4). Interestingly, the structural similarity matrix obtained from the structural models of the 22 GGDEF domains (Figure S1A) revealed that the GGDEF domain structures of proteins with the same domain composition and order, such as LIC_11125–LIC_11131, LIC_20181–LIC_20182 and LIC_12505–LIC_20180, belong to the same groups. In contrast, other GGDEF family members harboring the same protein domain architectures—LIC_13120 with LIC_20198, and LIC_11167 with LIC_12273 and LIC_13137^Lcd1^—did not cluster together, suggesting that these genes could belong to different (sub)families or may be evolving under higher substitution rates.

### 3.4. Proteins Containing EAL Domains

The Lic genome encodes two types of phosphodiesterases (PDEs) capable of degrading c-di-GMP: (1) those with EAL domains and (2) others with HD-GYP domains [86]. EAL domain-containing proteins are the most extensively studied and best-characterized PDEs, compared with proteins containing an HD-GYP domain [25,79,87,88]. EAL domain-containing proteins primarily function as phosphodiesterases, catalyzing the degradation of c-di-GMP into linear 5′-phosphoguanylyl-(3′→5′)-guanosine (5′-pGpG) (PDE-A activity). While some EAL domains may exhibit weak PDE-B activity (converting pGpG to two molecules of GMP), this secondary function occurs at rates considered physiologically insignificant in most bacteria [79,88,89]. Complete c-di-GMP degradation therefore typically requires two enzymatic steps: initial cleavage by PDE-As followed by pGpG hydrolysis by dedicated PDE-Bs enzymes like oligoribonuclease Orn (e.g., VC0341 in *V. cholerae* containing the RNase_T domain) [90]. Interestingly, accumulated pGpG can inhibit PDE-A activity, making PDE-B enzymes crucial for maintaining c-di-GMP homeostasis. Notably, our analysis revealed no RNase_T domain-containing proteins in the *L. interrogans* genome, suggesting alternative pGpG degradation mechanisms may operate in this organism. It is possible that proteins containing HD-GYP domains may hydrolyze pGpG in two GMP molecules to complete the second step in the *Leptospira* genus. Another possibility is that other proteins not yet described have the PDE-B activity in this group of bacteria.

A comparison with the RocR protein from *P. aeruginosa*, a model for the EAL domain [91], reveals that this domain is characterized by the ExL motif, residues for binding divalent cations (Mg^2+^ or Mn^2+^), catalytic residues necessary for c-di-GMP cleavage, including glutamic acid (Glu352), and loop 6 [19,22,79,91,92,93]. Loop 6, containing the DFG(T/A)GYSS motif, functional active site loop (loop 6) not only mediates the EAL dimerization but it is also important for regulation of substrate and cofactor binding, and it is therefore essential for enzymatic activity [91,94]. The three-dimensional structure of EAL domain-containing proteins adopts a modified TIM-barrel (β/α)_8_ fold [79,94]. The enzymatic activity of these phosphodiesterases is in general inhibited by ions of Ca^2+^ and Zn^2+^ [22,79,87,93]. EAL domain proteins exhibit activity as monomers, dimers, and oligomers in vitro assays [22,87,91,93,94,95]. However, homodimer formation is likely the predominant state in vivo, as it enhances protein stability and it is crucial for PDE activity in response to environmental stimuli [79,87,91,96].

**Figure 5:**
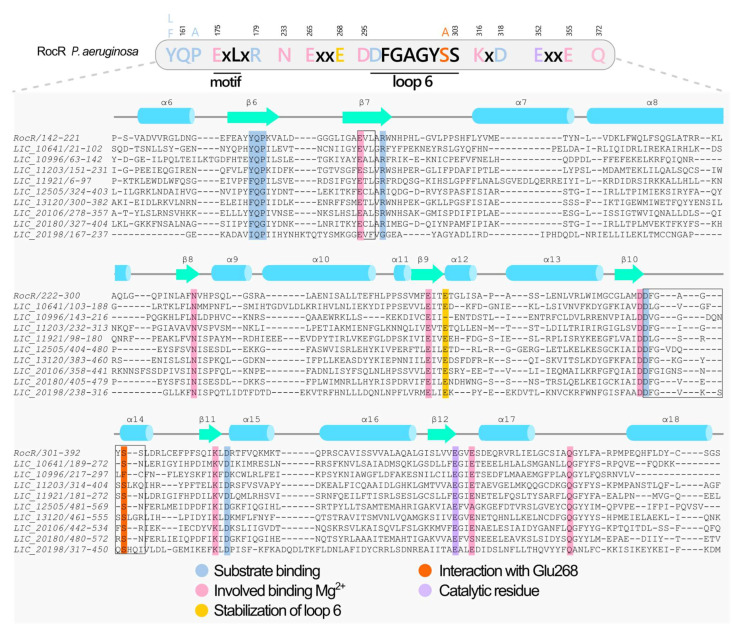
Characterization of the EAL domain of proteins from *L. interrogans* serovar Copenhageni strain Fiocruz L1-130. Structural alignments of EAL domains from *L. interrogans* serovar Copenhageni strain Fiocruz L1-130 via the DALI server, using the RocR (*locus_tag* PA3947, PDB 3SY8 [51]) from *P. aeruginosa* as a reference.

Through searches in the NCBI database looking for proteins containing EAL domains in the genome of Lic as well as in genomes of other *Leptospira* species, we found nine proteins containing the EAL domain. Of these, five are proteins containing both GGDEF and EAL domains (Figure 3). Using the RocR protein from *P. aeruginosa* as a reference, only LIC_20198 has the ExL motif degenerated. All identified enzymes appear capable of binding c-di-GMP, except LIC_20198, which does not have most of the important residues for c-di-GMP binding and catalysis (Figure 5). These alterations may disrupt substrate binding and, consequently, the enzyme’s catalytic function [91,92]. Therefore, eight out of nine proteins containing the EAL domain have the most residues involved in PDE-A activity and they are probably functional. LIC_12505 and LIC_20180 have FQG instead of YQP motif (YQP in RocR is important to substrate interaction). Moreover, LIC_12505 and LIC_20180 contain alanine and serine, respectively, in place of the glutamic acid residue (E355 in RocR from the E^352^xxE^355^ motif). Site-directed mutagenesis of the ExxE motif in the TBD1265 EAL domain showed that substitution of the first glutamic acid (E703) with alanine abolished PDE activity, whereas mutation of the second glutamic acid (E706) had no effect on enzymatic activity [93]. Therefore, substitutions at the equivalent position of glutamic acid (E355 in RocR) in LIC_12505 and LIC_20180 are unlikely to impair the enzymatic activity of these proteins. Therefore, these modifications may not affect PDE-A activity, as the other residues are conserved (Figure 5).

Interestingly, the structural similarity matrix derived from the models of the nine EAL domains (Figure S1B) revealed that, unlike GGDEF domains, EAL domain structures do not cluster with proteins that share the same domain architecture. Notably, the EAL domain of LIC_20198 shares greater structural similarity with LIC_10996, whereas LIC_13120 is more structurally similar to LIC_11203. The *Leptospira* proteins containing EAL domains that share the same domain architecture are: (i) LIC_10641 and LIC_11921, which possess an EAL-YkuI_C domain architecture; (ii) LIC_20198 and LIC_13120, which feature a GGDEF-EAL domain architecture; and (iii) LIC_12505 and LIC_20180, which exhibit a REC-GGDEF-EAL domain architecture (Figure 3).

### 3.5. Proteins Containing HD-GYP Domains

The genome of Lic encodes four annotated HD-GYP domain-containing proteins: LIC_10138, LIC_10139, LIC_11189, and LIC_11563. A fifth protein was identified through a BLASTp search in the NCBI database using the *Leptospira biflexa* LEPBI_I1560 HD-GYP protein as a query, revealing the protein LIC_10122. To facilitate sequence comparison, structural alignments of the HD-GYP domains were performed using Foldseek, with AlphaFold3-predicted models as input. The HD-GYP domain from the *P. marina* PmGH protein (PDB 4MDZ [53]) was used as the reference structure for a canonical HD-GYP domain. The catalytic HD-GYP domain belongs to the HD domain superfamily and features a characteristic five α-helix core (α6–α10), which provides the structural framework for coordinating a bi or trimetal center. In PmGH protein this coordination is mediated by eight conserved side-chain residues: E185, H189, H221, D222, H250, H276, H277, and D305, with K225 also contributing to the stabilization of the trinuclear iron center. The HD motif (H221 and D222), a defining feature of this domain, is located on helix α7. The HD-GYP domain has two additional C-terminal helices (α11 and α12) that pack against α7 and α10. The loop between α19 and α10 harbors the conserved GYP motif, where Y285 is oriented toward the metal-binding center. A conserved sequence motif, HHExxDGxGYPxxxxxxxI, defines HD-GYP proteins and includes a strictly conserved isoleucine (I294 in PmGH) [53]. Mutational analysis revealed that substitutions at key metal-coordinating residues (H189A, H221A, D222A, H250A, H276A, and H277A) significantly reduced phosphodiesterase activity, while mutations at E185A and D305A reduce the c-di-GMP hydrolysis. Interestingly, mutations in the GYP motif and conserved residues involved in c-di-GMP recognition (G284A, Y285A, P286A, I294A, R314A, and K317A) did not substantially affect catalytic activity. However, substitutions in conserved residues near the metal center (D183A, D308A, and K225A) significantly disrupted PDE function.

**Figure 6:**
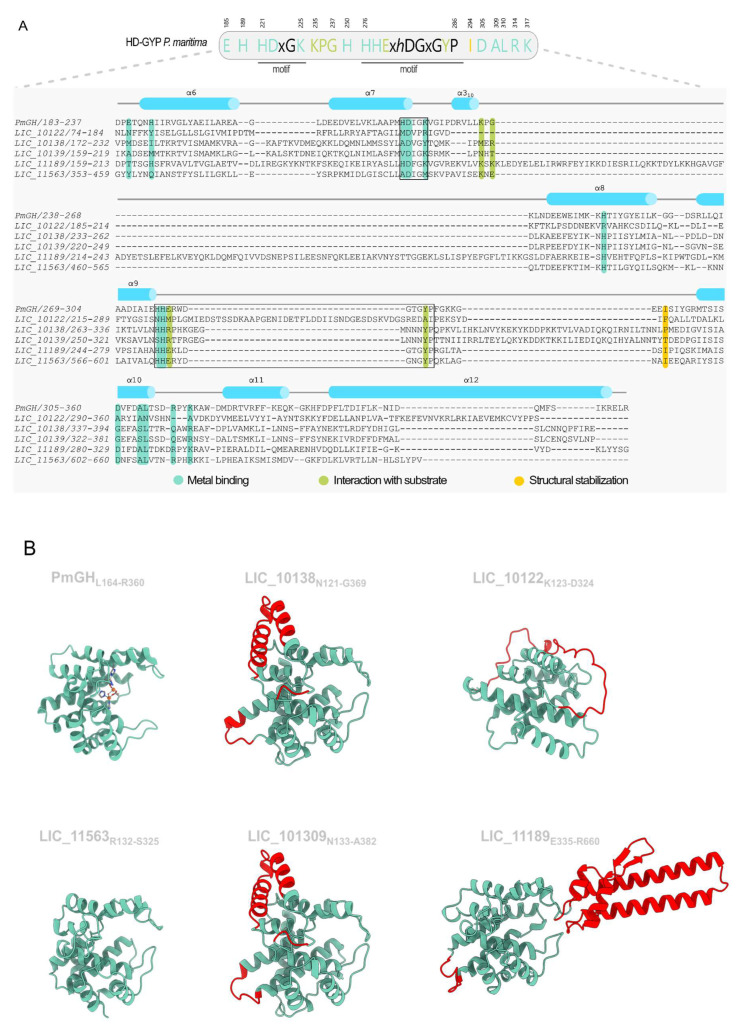
Characterization of the HD-GYP-containing proteins from *L. interrogans* serovar Copenhageni strain Fiocruz L1-130. (**A**) Sequence alignment of HD-GYPs from *L interrogans* serovar Copenhageni strain Fiocruz L1-130 based on structure performed on DALI using the PmGH (*locus_tag* PERMA_0986, PDB 4MDZ [53]) as reference. (**B**) Cartoon representation of the HD-GYP domains from *L. interrogans* predicted by AlphaFold (green). The HD-GYP domain from the *P. marina* PmGH protein (PDB 4MDZ [53]) was used as a reference, and the residues participating in metal coordination (E185, H189, D222, H250, and H277) are shown as sticks. The three Fe^3+^ ions are also shown as orange spheres. Extensions or insertions (relative to PmGH) in the HD-GYP domain from *L. interrogans* are colored in red.

Among the five proteins, only LIC_11189 retains the conserved residues characteristic of a canonical HD-GYP domain. LIC_11563 has most of the key residues suggesting a potential PDE (Table S4). In contrast, LIC_10138, LIC_10139, and LIC_10122 exhibit significant substitutions in catalytic residues, potentially impairing metal-binding capacity and phosphodiesterase activity. Nevertheless, it has been described that HD-GYP domains may accept more diversifications in the key residues without losing its enzymatic activity (Table S4) [53,79,86,97,98,99]. Notably, none of the HD-GYP proteins, including LIC_11189, possess the essential glutamate required for coordinating the third metal ion in a trinuclear catalytic center, suggesting that LIC_11189 may be restricted to a bimetal catalytic mechanism [53,100]. Furthermore, except for LIC_11189, the other HD-GYP proteins lack key residues necessary for substrate interaction, indicating that they are not only catalytically inactive but also unlikely to interact with c-di-GMP molecules (Figure 6A and Table S4). The five HD-GYP proteins share a three-domain architecture with distinct variations. LIC_10122, LIC_10138, LIC_10139, and LIC_11563 harbor the HD-GYP domain as the central domain, flanked by N- and C-terminal regions. In contrast, LIC_11189 localizes its HD-GYP domain to the C-terminal region, preceded by two sensory domains: REC and GAF (Figure 3 and Figure S2). LIC_10138 and LIC_10139 are highly similar in sequence and structure (Figure 6B and Figure S1C), consistent with a recent gene duplication event. In contrast, LIC_10122 is the most divergent, displaying the lowest sequence similarity to the other four proteins (Figure S1C). LIC_10138 and LIC_10139 each contain two additional helices immediately following the mutated GYP motif (Figure 6B). LIC_10122, however, harbors a poorly conserved GYP motif within a long, flexible C-terminal loop, with minimal structural similarity to PmGH (Figure S2). Interestingly, LIC_11189 features a unique insertion of a subdomain comprising two long helices, two short helices, and one beta-hairpin in the middle of the domain, further distinguishing it from the other HD-GYP proteins (Figure 6B and Figure S3).

### 3.6. The Insertion in the HD-GYP of LIC_11189 Is Widely Distributed in Response-Regulator HD-GYP Proteins

LIC_11189, the unique canonical HD-GYP protein in *L. interrogans*, is characterized by a REC-GAF-HD-GYP domain architecture. Foldseek and DALI searches using the HD-GYP domain insertion (LIC_11189 residues K416 to D536) as a query failed to identify homologous structures in the PDB, yielding only low-confidence matches aligning primarily with the two long helices. For example, DALI identified succinate dehydrogenase 2 from *Mycobacterium smegmatis* (PDB 6LUM [101]; z-score of 7.0 and RMSD equal to 2.5 Å), while FoldSeek PDB100 matched the RNAP-SutA complex from *P. aeruginosa* (PDB 7XL3 [102]; probability and *e*-value of 0.25 and ~9.1). In contrast, Foldseek AFDB50 revealed highly similar structures across diverse bacterial species, with the insertion consistently embedded within HD-GYP domains. Remarkably, even when only the aminoacid insertion sequence was used as a query, most of the retrieved proteins displayed a domain architecture closely resembling that of LIC_11189, spanning bacteria beyond the *Spirochaetota* phylum. In phylogenetically distant species, including *Vibrio mediterranei* (Taxonomy ID: 689), *Vibrio variabilis* (Taxonomy ID: 990271) [103], *Paraburkholderia caballeronis* (Taxonomy ID: 416943), and *Thalassomonas viridans* (Taxonomy ID: 137584) [104], the insertion contained an extended flexible loop between the two short β-strands that form a hairpin (Figures S3 and S4). The HD-GYP insertion domain was found closely associated with a preceding GAF domain, which was sometimes incomplete, and N-terminal domains that were frequently highly or moderately structurally similar to REC domains. Interestingly, this insertion appears strongly linked to HD-GYP domains fused to response regulator domains, suggesting a potential ancient role in two-component system signaling.

Sequence conservation analysis revealed conserved hydrophobic residues likely mediating apolar interactions that stabilize the connection between the two long helices with the short secondary structure elements (Figure S5). In addition, highly conserved polar residues are positioned near the phosphodiesterase active site, adjacent to the substrate-binding pocket (Figure S5). Notably, such structure has not been described in previously characterized HD-GYP proteins. We hypothesize that this unique architecture may represent a novel mechanism for interaction with c-di-GMP or confer increased permissiveness for binding other cyclic dinucleotides. We named this domain the HD-GYP insertion domain (HdiD).

### 3.7. Potential Distant Members of DUF3391 Family

The limited information available on the DUF3391 (PF11871) domain poses challenges in elucidating its relationship with the N-terminal domain of HD-GYP-containing proteins from *L. interrogans* such as LIC_10122, LIC_10138, LIC_10139, and LIC_11563. According to InterPro, DUF3391 is an uncharacterized N-terminal domain frequently associated with HD-GYP proteins across diverse bacterial taxa. Among the HD-GYP proteins in *L. interrogans*, only LIC_11563 has its N-terminal domain annotated as DUF3391 in the CDD. In contrast, CDD and InterPro analyses failed to identify domains in the N-terminal regions of LIC_10122, LIC_10138, and LIC_10139. Excluding the HD-GYP domain, the N-terminal portions of the four proteins exhibit comparable lengths, ranging from 119 to 132 residues (Figure S6A). BLASTp searches using these N-terminal regions identified homologs exclusively within the *Spirochaetota* phylum, predominantly in the *Leptospira* genus (more information in Supplementary Materials). Canonical DUF3391 domains exhibited four to five antiparallel β-strands and one to two α-helices within the globular domain, followed by a C-terminal α-helix. In some cases, DUF3391 domains appeared independently, without an associated HD-GYP domain; however, genomic analyses often revealed downstream HD domains separated by frameshift mutations, as observed in G3N55_00320 (Taxonomy ID: 1765737) [105], which contains a premature stop codon disrupting the HDOD domain (Figure S6B). The globular domains of *L. interrogans* proteins were predicted to contain 4–6 antiparallel β-strands with a C-terminal α-helix. Notably, LIC_10122 and LIC_11563 contain an additional α-helix preceding the last β-strand, while LIC_10138 and LIC_10139 exhibit topologies similar to canonical DUF3391 domains but with an α-helix preceding β4, followed by two additional β-strands before the C-terminal α-helix (Figure S6A).

A multiple sequence alignment, as described by Galperin and co-authors revealed high conservation of key hydrophobic residues among the four *L. interrogans* N-terminal domains and canonical DUF3391 domains, suggesting functional significance (Figure S7A) [86]. Interestingly, despite its DUF3391 annotation, LIC_11563 clustered phylogenetically with the other *L. interrogans* proteins rather than with canonical DUF3391 domains (Figure S7B). The DUF3391 domain remains poorly characterized, with substantial variability across its members. Structural predictions and sequence alignments suggest that many unannotated N-terminal regions fused to HD-GYP domains may belong to this family. Furthermore, analysis of domain architectures identified in InterPro for the DUF3391 Pfam model revealed that this domain is consistently located at the N-terminus of proteins fused to other domain families, such as T2SSB (a member of the PilP superfamily), and in a few isolated cases associated with enzymatic domains, including glucose/sorbosone dehydrogenases and thioredoxin-like alkyl hydroperoxide reductases (AhpC/TSA). Interestingly, in addition to the many fusions to HD homologs, some examples of fusions to other signal transduction effector domains are also observed, such as fusions to C-terminal SpoIIE and methyl-accepting chemotaxis proteins (MCPsignal). These patterns of C-terminal fusions suggest that DUF3391 could have a regulatory role, acting as a sensor that regulates the activity of the enzymatic/effectors domains. Still, additional, experimental and computational studies will be necessary to clarify the biological role of these domains and refine their classification. Accordingly, we propose that the N-terminal domains of *L. interrogans* HD-GYP-containing proteins represent a divergent subgroup within the DUF3391 family.

### 3.8. A Novel C-Terminal Domain in HD-GYP-Containing Proteins

In addition to the N-terminal DUF3391 domain present in LIC_10122, LIC_10138, LIC_10139, and LIC_11563, all four proteins contain an unannotated C-terminal domain from 68 to 120 amino acids in length. While CDD and InterPro analyses failed to identify this region as a known domain, AlphaFold predictions yielded models with low confidence for LIC_10122_E325-K433_ and LIC_11563_L326-A394_ but high confidence for the paralogs LIC_10138_S371-A489_ and LIC_10139_L383-A50_. PDBsum [106] topology representations revealed conserved secondary structures in LIC_11563_L326-A394_, LIC_10138_S371-A489_, and LIC_10139_L383-A503_, characterized by five to six antiparallel β-strands followed by a C-terminal α-helix, forming a barrel-like structure. In contrast, LIC_10122_E325-K433_ is predicted to begin with a long helix preceding two pairs of β-strands (Figure S8). Structural analysis using PDB90 and Foldseek PDB100 did not reveal a potential function for this new domain. The C-terminal domains of the proteins LIC_10122, LIC_10138, and LIC_11563 show divergent sequences (more information in Supplementary Materials and Figures S9–S15). The predicted three-dimensional models and subsequent structural analyses did not reveal clear structural similarities that would allow us to infer their functions or establish homology (see Supplementary Materials for details). Therefore, further studies are needed to better understand the nature of these domains found in the C-terminal region of these proteins containing the HD-GYP domain of *L. interrogans*.

### 3.9. Proteins Containing PilZ Domain

**Figure 7:**
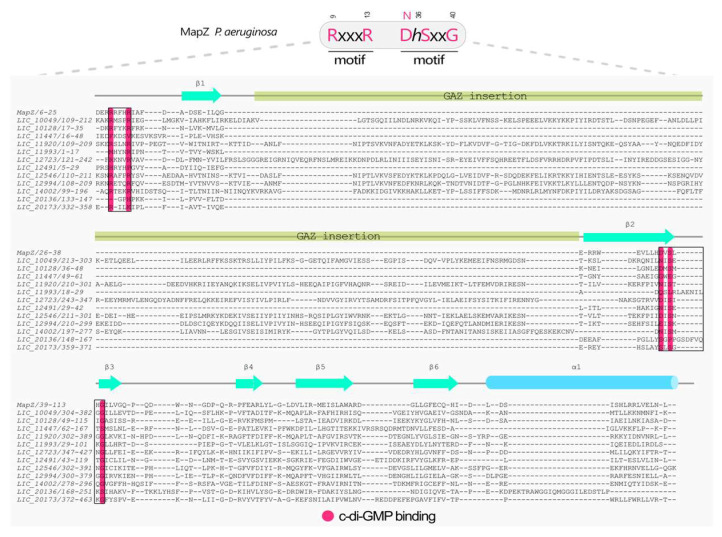
Characterization of the PilZ domain of proteins from *L. interrogans* serovar Copenhageni strain Fiocruz L1-130. Structural alignments of PilZ domains from *L. interrogans* serovar Copenhageni strain Fiocruz L1-130 via the DALI server, using the protein MapZ (*locus_tag* PA4608, PDB 5HTL [108]) from *P. aeruginosa* as a reference.

The PilZ domain was one of the first specific binding domains identified for the c-di-GMP and remains among the most extensively studied c-di-GMP effector proteins [107]. PilZ domains are characterized by a closed β-barrel containing six antiparallel β-strands followed by an α-helix [21]. This unique structural architecture, encoded in most bacterial genomes, underscores the importance of the PilZ domain in regulating cellular processes mediated by c-di-GMP. Canonical PilZ domains contain two conserved motifs essential for c-di-GMP binding. The first motif is an arginine-rich sequence (RxxxR) located in a loop preceding the first β-strand, while the second motif (D/N)xSxxG is found at the end of the second β-strand [33]. Binding of c-di-GMP often induces conformational changes in the PilZ domain, facilitating interactions with other proteins or modulating cellular activities. Hydrophobic residues within the domain core are critical for maintaining structural integrity and regulating the activity of associated domains [107]. Genome analysis of Lic, using NCBI database searches, local alignments, and orthology investigations, identified 12 proteins containing PilZ domains (Figure 3). These proteins either possess isolated PilZ domains or PilZ domains coupled with additional domains. Among them, LIC_10128 emerged as a functional PilZ, containing the conserved RxxxR and (D/N)xSxxG motifs necessary for c-di-GMP interaction. In contrast, five proteins (LIC_20173, LIC_20136, LIC_12491, LIC_11993, and LIC_11447) were classified as non-functional PilZ variants due to the absence of the conserved arginines required for c-di-GMP binding (Figure 7).

Six out of twelve identified proteins are annotated in UniProt as containing the DUF1577 domain, which, through in-depth bioinformatic analyses, was revealed to be a fusion of three distinct domains: the PilZN domain (formerly YcgR_N), a GAF-like domain (known as GAZ domain), and a C-terminal PilZ domain [20]. The PilZ domain has the presence of the GAZ domain, which has lost its dimerization capability due to the absence of the α1 secondary structure element. Despite the domain insertion, the c-di-GMP–binding regions remain conserved, suggesting preservation of functionality. Consistently, LIC_11920 retains the ability to bind c-di-GMP [20].

The group of PilZ proteins sharing the same domain architecture includes the YcgR^GAZ^ proteins (LIC_10049, LIC_11920, LIC_12546, LIC_12723, LIC_12994, and LIC_14002) and four proteins containing only the PilZ domain (LIC_10128, LIC_11447, LIC_11993, and LIC_12491) (Figure 3 and Figure 7) [20]. Structural similarity analysis showed that the YcgR^GAZ^ proteins clustered together, whereas the single-domain PilZ proteins were structurally divergent (Figure S1D). Additionally, two novel domain architectures were identified in Lic PilZ proteins, as described below, suggesting potential diversification of PilZ-related signaling mechanisms within *Leptospira*.

### 3.10. The LIC_20136 and LIC_20173 Represent Novel PilZ-like Families with Unique Domains Architecture

DeepTMHMM predictions and the top-ranked AlphaFold model for LIC_20136 indicate that this protein contains a transmembrane segment (residues 16–38), followed by a globular pentahelical domain (residues 39–131), which we designated as D1 domain. This region is connected to a non-canonical PilZ domain (residues 132–241) and a long C-terminal domain (residues 243–707), which we designated as D2 domain (Figure S16A). Structural similarity searches of D1, conducted using the DALI, yielded borderline significant alignments (z-score less than 5.6). The closest structural match was to the C-terminal domain described as ribosome-associated complex head domain (RAC_head, PF_16717) of the Zuotin protein (PDB 7X34 [109]) with a C_α_ RMSD of 3.3 Å and 10% identity of sequence to this domain. The RAC_head has also been described as the C-terminal four-helix bundle (4HB) domain [110,111]. Notably, the final α-helix of D1 is absent in the 4HB domain, indicating a degree of structural divergence. The first helix of the 4HB domain mediates interactions with ES12 (helix 44 of 18S rRNA) through six lysine residues, which are not present in LIC_20136 D1 (Figure S16B). Nevertheless, the presence of positive residues in this helix, along with the multiple sequence alignment representation by WebLogo diagram shows conserved positively charged residues in this region (Figure S16D), suggests a potential RNA-binding function. YcgR homologs containing NpzN domains (“N-terminal to PilZN”), referred to as YcgR^NpzN^, share a conserved domain architecture. This consists of a transmembrane helix, followed by the NpzN domain—characterized by four α-helices arranged in perpendicular pairs—and the classical PilZN and PilZ domains typical of the YcgR family. Although the D1 domain resembles the NpzN domain, we do not observe the same domain architecture [20]. This discrepancy may be due to limitations in the structural prediction of the D1 domain.

In the case of the D2 domain, residues 239 to 707, searched by domain characterized using the interPro and CD-search, did not identify any domain. Searches for homology detection and structure prediction by HMM-HMM comparison (HHpred) also did not identify any domain. Structural similarity analysis using the DALI server and the structure predicted by AlphaFold observed structural similarities of residues 413 to 650 of the D2 domain with: protein Zmp1 from *Clostridioides difficile* (strain 630) that has the Pro-Pro endopeptidase domain described by the PFAM as ATLF domain (Anthrax toxin lethal factor, N- and C-terminal domain, PF07737, IPRO14781) with Z-score of 6.4 (PDB 6R4Z [112]); the ATLF domain of the anthrax lethal factor protein (residues 63 to 279) with Z-score of 5.8 (PDB 1JKY [113]); the ATLF domain of the certhrax toxin from *Bacillus cereus,* residues 2 to 226, with Z-score of 5.7 (PDB 4FXQ [114]); the Peptidase_M4 domain (Thermolysin metallopeptidase, catalytic domain) of the zinc metalloprotease ProA of *Legionella pneumophila*, residues from 222 to 385, with Z-score of 5.5 (PDB 6YA1 [115]); the ATLF-like domain of the Edema factor exotoxin of the Anthrax bacteria, residues 60 to 273, with Z-score of 5.5 (PDB 1XFU [116]) (Figure S16C). The active site of Zmp1 is built by the H_142_E_143_xxH_146_ motif, along with residues E_140_, W_103_, and Y_178_ (Figure S16C). However, only Y_178_, corresponding to Y_608_, is present in the D2 domain. This analysis suggests that the D2 domain is unlikely to bind zinc or function as a protease in the same manner as the Zmp1 protein. The ATLF domain of the anthrax lethal factor (LF) protein binds to the membrane-translocating component of anthrax toxin, the protective antigen (PA), which is crucial for host cell binding and facilitates the entry of LF. Notably, the ATLF domain lacks the HExxH motif required for zinc binding and protease activity. The same thing happens with the ATLF domain of the certhrax toxin from *B. cereus*. In the case of the toxin ProA of *L. pneumophila* the Peptidase_M4 domain is responsible for cleaving a broad spectrum of substrates such as casein or gelatin and promotes infection of human lung tissue. The protease activity is mediated by the same motifs as the ATLF domain, the HExxH motif and other accessory residues, most of which are absent in the D2 domain. Based on this analysis the D2 domain is homologous to the ATLF domain but lacks the residues important to zinc coordination and catalysis and may instead work, as proposed for the ATLF domain of the anthrax lethal factor (LF) protein and the certhrax toxin from *B. cereus* that are involved in protein–protein interactions. LIC_20136 is widely distributed within the genus *Leptospira*, but we did not identify it in other genera (Figure S16E).

Domain characterization using InterPro and CD-Search revealed that LIC_20173 contains a PrsW domain in its N-terminal region (residues 1–210) but no additional domains were detected. To further investigate its domain architecture, we analyzed the AlphaFold-predicted structural model of LIC_20173 using the DALI and FoldSeek server, which identified a PilZ domain in the C-terminal region (Figure S17A). Additionally, DeepTMHMM predictions indicated that the protein possesses nine transmembrane helices: seven within the PrsW domain and two additional transmembrane helices (Figure S17A). The only functionally characterized PrsW domain is the protein YpdC of *Bacillus subtilis,* which has four motifs, correspond to E_75_E_76_xxK_79_, followed by F_110_xxxE_114_, a conserved histidine (H138) as the third motif, and the fourth motif H_175_xxxD/N_179_ [117] (Figure S17A,B). The motifs E_75_E_76_xxK_79_ and H_175_xxxD/N_179_ are located in the transmembrane region of the protein (Figure S17E) [118].

Site-directed mutagenesis in YpdC indicates that either double point mutation of the two conserved glutamates in the first motif (E75A/E76A), or a single mutation of the conserved histidine in the fourth motif (H175A), are of functional importance. Using YpdC from *B. subtilis* as a model for the PrsW domain active site, sequence alignment (Figure S17C,D) shows that LIC_20173 contains the two conserved glutamate residues in the first motif and a histidine residue in the fourth motif, suggesting that the PrsW domain of LIC_20173 is catalytically active. The PrsW domain of YpdC and probably in the case of LIC_20173 have an N-terminal domain starting in the periplasm, with these proteins set, probably, by an insertase protein YidC. The YidC insertase follows the positive inside rule to set the protein position in the inner membrane [119]. Probably the protein LIC_20136 also assembles in the inner membrane in the same way as LIC_20173, with the N-terminal region located in the periplasm and the C-terminal domains located in the cytoplasm in a way that the PilZ domain is located in the cytoplasm.

### 3.11. Proteins Containing the MshEN Domain in L. interrogans Serovar Copenhageni strain Fiocruz L1-130

**Figure 8:**
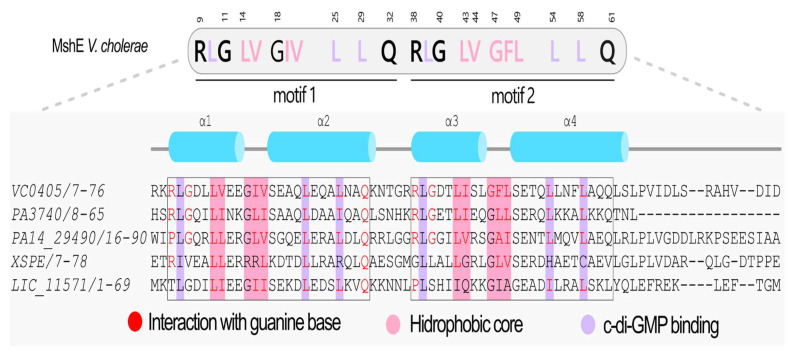
Characterization of the MshEN domain of proteins from *L. interrogans* serovar Copenhageni strain Fiocruz L1-130. In the MshEN domain, c-di-GMP is accommodated by two 24-residue motifs [RLGxxL(L/V/I)xxG(L/V/I/F)(L/V/I)xxxxLxxxLxxQ], connected by a 5-residue linker, forming a complete 53-residue-long domain. The MshEN domain used as a model MshEN_N (l*ocus_tag* VC_0405, PDB: 5HTL [32]) of *V. cholerae*, PA3740 (probably functional), and PA14_29490 (experimentally demonstrated to be functional) of *P. aeruginosa* [31,32,121], XspE of *X. campestris* strain 17 (PDB: 2D27 [122]), which has been shown that it is a degenerate MshEN domain.

The identification of proteins containing the MshEN domain in the genome of *Leptospira interrogans* serovar Copenhageni strain Fiocruz L1-130 was initiated by searching the NCBI database for proteins annotated with this domain using the genus *Leptospira* as a query. The resulting dataset was then used to screen the *L. interrogans* genome in an interactive manner. This strategy, described in detail in Section 2, enabled the detection of proteins not previously annotated as containing the MshEN domain. Using this approach, we identified a single protein, LIC_11571, with an MshEN domain in its N-terminal region in the Lic genome. This gene is located in the cluster of the type II secretion system (T2SS) and encodes the GspE protein (T2SS ATPase). In the MshEN domain, c-di-GMP is accommodated by two motifs connected by a five-residue linker [32] (Figure 8). Wang and collaborators studied the MshEN domain from *V. cholerae* (locus_tag VC0405), a protein associated with the formation of the mannose-sensitive hemagglutinin type IV pilus (T4P) [32,120]. A VC0405 homolog from *P. aeruginosa*, PA14_29490, also contains a T2SSE ATPase domain and is involved in the T2SS [33]. Both proteins interact with c-di-GMP through their MshEN receptor domains. MshE proteins exhibit high-affinity binding to c-di-GMP, with the interaction occurring in their N-terminal T2SSE_N domains (hereafter referred to as MshEN). In contrast, the ATPase domain, which binds ATP, does not interact with c-di-GMP. Notably, residues R9 and Q32 of MshEN from *V. cholerae* play pivotal roles in c-di-GMP binding. Indeed, crystallographic studies have revealed that MshEN contains two 24-residue motifs connected by five non-conserved residues, cooperatively forming a 53-residue domain that interacts with c-di-GMP [32]. This c-di-GMP-binding domain is found across various bacteria, often fused with ATPase, glycosyltransferase, or other domains. These proteins exhibit c-di-GMP binding affinities with dissociation constants (*K*_D_) ranging from 14 nM to 0.5 μM, highlighting MshEN as a highly sensitive c-di-GMP receptor capable of participating in diverse c-di-GMP-mediated bacterial processes [32]. The crystal structure of the MshEN-c-di-GMP complex from *V. cholerae* reveals two subdomains: an N-terminal MshEN_N four-helix bundle (α1-α4) and a C-terminal MshEN_C subdomain featuring antiparallel β-strands (β1-β3) flanked by three helices. MshEN_N subdomain binds c-di-GMP primarily and the D108 in MshEN_C may also contact the guanine base of c-di-GMP.

The electrostatic surface of the MshEN_N subdomain of *V. cholerae* is predominantly positive, composed of residues K5, R7, K8, R9, and R38, which stabilize c-di-GMP interactions via electrostatic forces while the guanine bases of c-di-GMP are stabilized by hydrophobic interactions with L25, L29, and L39. Mutation analysis showed that R9A/D12A, R88A/R89A, and D108A/D111A variants reduced c-di-GMP interactions, while R146A/R147A and E191A/D192A mutations did not affect c-di-GMP binding. Figure 8 illustrates the conserved residues of this protein, highlighting the critical roles of R9, L14, and Q32 in c-di-GMP binding, with hydrophobic interactions and hydrogen bonds further stabilizing the complex. The LIC_11571 does not have any arginines at the motif RLG in the motif 1 and 2, suggesting that it is unlikely to function as a c-di-GMP receptor.

### 3.12. Distribution of c-di-GMP Genes Among L. interrogans Isolates

**Figure 9:**
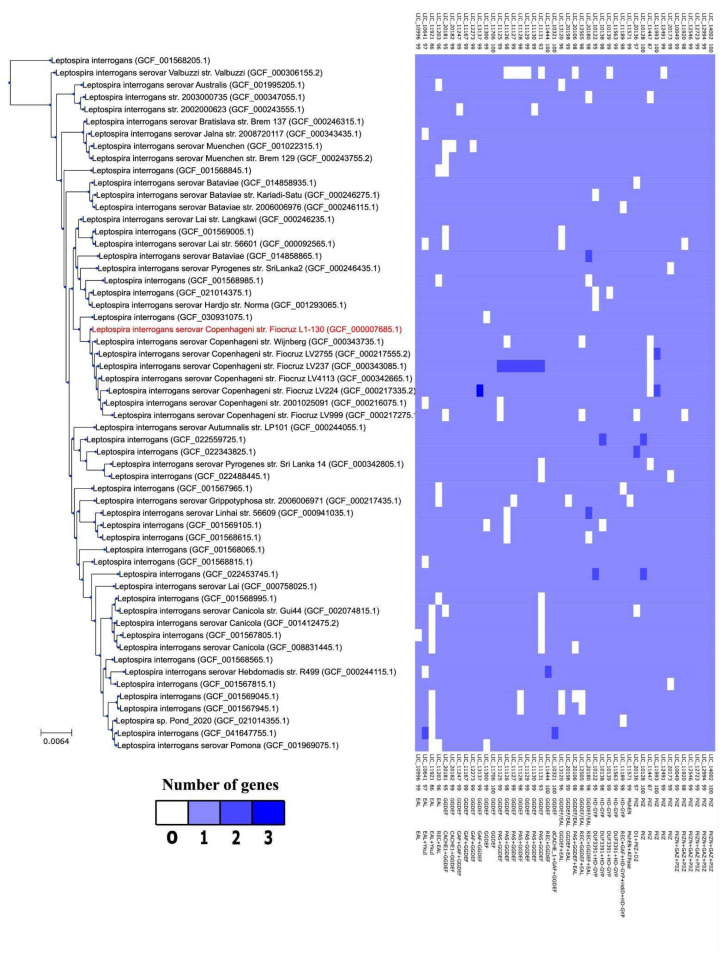
Phylogeny of representative *L. interrogans* isolates. The tree on the left was inferred using the neighbor-joining method based on genome similarity estimates calculated with the FastANI (version 1.34) software. The heatmap represents the number of genes in each orthologous group related to c-di-GMP turnover in each genome. The header and footer indicate, from top to bottom: (i) the gene identifiers of *L. interrogans* serovar Copenhageni strain Fiocruz L1-130; (ii) the percentage of genomes in the total pangenome that contain members of each group; (iii) the family or subfamily of the c-di-GMP–binding domain; and (iv) the domain architecture of the proteins in each group. The tree was rooted using the midpoint method, and the order of its leaves was used to order the heatmap rows. The figure was rendered using the ete3 library [123]. Figure S19 shows the version of this phylogeny with all 365 isolates.

To evaluate whether the diversity and complexity of the c-di-GMP gene content was conserved across different *L. interrogans* serovars and isolates, we analyzed the pangenome composition of a selection of 365 high quality *L. interrogans* genomes downloaded from the RefSeq database. These genomes were selected based on completeness and contamination estimated by CheckM2 and the groups of orthologs and paralogs were defined using the PanACoTA pangenome toolkit. Our analysis revealed that the *L. interrogans* pangenome is most likely an open pangenome, thus implying that the repertoire of accessory genes is expected to increase as new isolates are sequenced (Figure S18). We found that 27 of the 44 c-di-GMP-related genes identified in *L. interrogans* serovar Copenhageni strain Fiocruz L1-130 are present in more than 99% of the isolates (Table 1) and, therefore, are part of the core genome of this species. Although the overall pattern of presence and absence of the c-di-GMP orthologous groups in *L. interrogans* isolates suggest a mostly random pattern of gains and losses, a phylogenomic tree based on nucleotide sequence similarity revealed that certain gene losses are shared by the members of monophyletic clades corresponding to known serovars (Figure 9). The loss of LIC_11447, a protein characterized by the presence of a single non-functional PilZ domain, in several closely related Copenhageni and Icterohaemorrhagiae isolates, is one example of the pattern of lineage specific gene loss of some genes. Another example is the absence of LIC_11921 homologs in several isolates of the Pomona and Canicula serovars. This protein contains the fusion of a N-terminal EAL phosphodiesterase domain and a C-terminal YkuI domain. YkuI domains belong to the CACHE superfamily and could play a role in these proteins as both a sensor and a dimerization domain, implying that these two serovars may share the loss of a common intracellular regulator. Canicola strains also share the loss of LIC_11131, one of the paralogous PAS and GGDEF fusions present as in-tandem duplications at a single loci in many *L. interrogans* serovars. The cluster of seven in-tandem PAS + GGDEF paralogs, from LIC_11125 to LIC_11131, is greatly reduced in all isolates of the Valbuzzi serovar, where only LIC_11125 and LIC_11130 remain.

## 4. Discussion

The production of c-di-GMP in bacteria orchestrates diverse physiological processes, including biofilm formation, enhanced resistance to environmental stressors, modulation of antibiotic susceptibility, and regulation of virulence [16,25,28]. The precise regulation of c-di-GMP synthesis is crucial for bacterial fitness, as it sustains a coordinated signaling network by maintaining well-defined intracellular concentrations of this second messenger, thereby preserving cellular homeostasis [124,125]. To elucidate how c-di-GMP influences the behavior of *L. interrogans*, it is essential to identify the proteins involved in its signaling pathways. In *L. interrogans*, c-di-GMP production is associated with biofilm formation, contributing to infection persistence within hosts and reservoir animals, as well as survival under adverse physical and chemical conditions, including high UV radiation, fluctuating salinity, pH variations, and antimicrobial exposure [11,16,18].

In this study, we developed a novel strategy to identify proteins containing GGDEF, EAL, HD-GYP, PilZ, and MshEN domains. Our analysis combined amino acid sequence-based searches with three-dimensional structural bioinformatics to systematically identify c-di-GMP signaling proteins. We first built a preliminary database by querying UniProt with c-di-GMP–related keywords in combination with the term *Leptospira*, complemented by searches in NCBI to recover previously annotated genes in the genus. To address inconsistencies across annotations, we performed manual BLASTp analyses using the *L. interrogans* genome as the query, since some genes were annotated in other species but absent in *L. interrogans*. The candidate proteins were then validated through structural prediction with AlphaFold3, followed by homology comparisons using FoldSeek and DALI, and manual inspection. Additional domain analyses were conducted using the Conserved Domain Database (CDD), InterPro, and Pfam. Unlike methodologies previously applied, which were largely restricted to primary sequence analysis (e.g., BLAST, HMM) and automatic annotation, our approach integrates structural validation and interactive BLAST searches across different *Leptospira* species.

Using this approach we identified 44 proteins associated with c-di-GMP signaling in *L. interrogans*, including seven proteins not well annotated as having a c-di-GMP–related domain in the KEGG, CD-Search, or InterPro databases. These seven proteins include two with PilZ domains (LIC_20136 and LIC_11993), one with an HD-GYP domain (LIC_10122), and four with GGDEF domains (LIC_10321, LIC_11167, LIC_11247, and LIC_11706) (Figure 3). The bacterium exhibits a high density of proteins involved in c-di-GMP metabolism, encompassing 17 proteins with GGDEF domains, four with EAL domains, five with both GGDEF and EAL domains, five with HD-GYP domains, 12 with PilZ domain, and one with MshEN domain. This corresponds to a c-di-GMP intelligence quotient (IQ)—the density of turnover proteins per megabase pair (Mbp) of genome—of 9.4 (44/4.69) per Mbp, surpassing the average of many bacteria (4.11 per Mbp) [79,126,127,128]. The estimated value of 9.4 per Mbp for *L. interrogans* may represent an overestimation. Based on our analysis, 21 proteins are predicted to function as either PDEs or DGCs, while seven proteins with PilZ domains are likely c-di-GMP receptors. Nevertheless, not all enzymes or receptors are necessarily involved directly in c-di-GMP turnover; instead, some may participate in signal transduction. A well-characterized example is FimX from *Xanthomonas citri*, which contains degenerated GGDEF and EAL domains. In this case, the EAL domain of FimX binds c-di-GMP and also interacts with a degenerated PilZ domain, and the complex regulates type IV pilus function. The PilZ domain appears to act as an adapter linking FimX to PilB, the ATPase required for PilA polymerization [129]. Therefore, inclusion of all proteins containing GGDEF, EAL, HD-GYP, PilZ, and MshEN domains in the IQ calculation represents a reasonable and systematic approach. It should be noted, however, that other c-di-GMP receptors, such as riboswitches, are not accounted for in this analysis.

This high c-di-GMP IQ, suggesting that *L. interrogans* has a highly complex and likely finely tuned regulatory network for c-di-GMP, consistent with its need to adapt between environmental and host-associated lifestyles. The present study focused on identifying GGDEF, EAL, HD-GYP, PilZ, and MshEN domains through structural homology, leveraging their established catalytic or c-di-GMP binding residues as curated in the NCBI c-di-GMP database [22,30,31,32]. All analyses were conducted in silico, including assessments of degenerate domains. However, predicting novel receptors remains a considerable challenge using bioinformatics alone, owing to the structural and functional diversity of these receptors. Many c-di-GMP receptors lack well-defined conserved motifs, and binding can occur at multiple sites depending on the c-di-GMP receptor domain via distinct molecular interactions, complicating identification of new c-di-GMP receptors based solely on known sequences or structural templates [22,28,130]. In *L. interrogans*, 22 diguanylate cyclases (DGCs) have been identified, 13 are predicted to be functional based on the conservation of residues essential for enzymatic activity (Figure 4). Even degenerate GGDEF domains can act as GTP or c-di-GMP sensors or regulate associated EAL domains, despite losing catalytic activity [19,22]. Experimental validation is needed to confirm these findings since bacterial species exhibit diversity in motif functionality. Moreover, some proteins with GGDEF domain containing the canonical catalytic residues can be inactive such as GdpS from *Staphylococcus aureus* and *S. epidemitis*, c21220 and B54690 from *Sinorhizobium fredii*, HmsT from *Yersinia pestis* [22,131,132,133]. In *L. interrogans*, many DGCs can not bind GTP due to mutations in guanosine-binding residues but they may still function as c-di-GMP receptors or participate in protein–protein interactions. Among the DGCs of *L. interrogans*, only LIC_11706 may function as a c-di-GMP receptor. The synthesis of c-di-GMP is often modulated by negative feedback through two inhibitory allosteric sites: the I and I’ sites. Binding of c-di-GMP to these sites immobilizes DGCs in a non-catalytic state [22,134]. Most *L. interrogans* DGCs appear to have functional inhibitory sites, with the exception of LIC_12273. Overall, the functionality of *L. interrogans* GGDEF domain proteins is shaped by a complex interplay of catalytic site conservation, motif variation, and regulatory feedback mechanisms. Experimental studies will be essential to unravel the physiological relevance of these DGCs and their contributions to bacterial signaling and environmental adaptation.

The EAL domain is widely recognized for its role in proteins that regulate cyclic diguanylate monophosphate (c-di-GMP) metabolism. This domain is associated with phosphodiesterase activity, catalyzing the degradation of c-di-GMP into pGpG, which is subsequently hydrolyzed into two molecules of GMP (Figure 1). Bacteria often encode multiple proteins containing EAL domains, which may exist either as standalone proteins or fused with other signaling or output domains. This structural diversity enables EAL-containing proteins to participate in complex regulatory networks, where different domain combinations confer distinct functional properties, including sensitivity to various stimuli or interactions with other signaling molecules. Thus, EAL domains, whether isolated or combined with other domains, play diverse roles in regulating cellular processes mediated by c-di-GMP. Genome analysis of *L. interrogans* identified nine EAL family members containing the conserved ExL and DFG(T/A)GYSS motifs, which are critical for coordinating metal ions like magnesium (Mg^2+^) or manganese (Mn^2+^). The glutamate in loop 6 and arginine residues within the ExLxR motif directly participate in this coordination, stabilizing the cofactors essential for phosphodiesterase activity [91,92]. These metal ions play a key role for catalyzing c-di-GMP hydrolysis, ensuring proper EAL domain function, and regulating intracellular signaling pathways. Using the RocR protein from *P. aeruginosa* as a reference, several Lic proteins appear capable of binding c-di-GMP, except LIC_20198, which does not have some residues for c-di-GMP binding and catalysis having a possible PDE activity (Figure 5). Interestingly, LIC_20198 carries a substitutions of RocR tyrosine 160 for isoleucine and arginine 179 for glycine, which may disrupt substrate binding and enzymatic activity [79,91,92]. However, transposon insertion studies by Thibeaux and co-authors revealed that disrupting the EAL domains of LMANV2_v2_270021 (LIC_20180) and LMANV2_v2_90001 (LIC_20198) increases biofilm production, suggesting these enzymes likely degrade c-di-GMP in vivo [16]. Additional functional experiments are necessary to clarify the activity and regulatory roles of these enzymes. Negative feedback regulation of c-di-GMP degradation is vital for bacterial homeostasis, maintaining appropriate intracellular concentrations of this secondary messenger. EAL domain-containing phosphodiesterases play an important role in this process by hydrolyzing c-di-GMP to pGpG, while responding to intracellular levels of pGpG, thus balancing synthesis and degradation, and preserving cellular equilibrium.

Among c-di-GMP turnover domains, HD-GYP is notably less common and less studied than EAL domains, and therefore, on occasion, described as the “neglected small sibling” [86,135]. The HD-GYP catalytic mechanism depends on di-metal or tri-metal centers coordinated by specific amino acid residues. The HxxxHDxxxHxxxHxxxD motif is essential for phosphodiesterase activity, as it organizes the dinuclear structure [100,135]. A third ion may be coordinated by an additional glutamate residue, such as E185 in PmGH [53]. In *L. interrogans*, none of the HD-GYP proteins contain this extra glutamate necessary for tri-metal coordination. Among them, LIC_11189 is the only protein retaining canonical residues required for di-metal center formation, whereas LIC_10122, LIC_10138, and LIC_10139 contain mutations in critical residues, such as the histidine within the HD motif. The LIC_11563 protein harbors conserved residues essential for phosphodiesterase (PDE) activity, supporting its classification as a putative PDE enzyme, in agreement with the findings of Xiao and collaborators (2018) [18]. Still, previous studies demonstrated that site-directed mutagenesis of any residue responsible for metal ion coordination abolishes phosphodiesterase activity [53]. This strongly suggests that LIC_10122, LIC_10138, and LIC_10139 are catalytically inactive due to their inability to coordinate the di-metal center. Furthermore, only LIC_11189 and LIC_11563 conserve the residues required for c-di-GMP binding, whereas LIC_10122, LIC_10138, and LIC_10139 lack these residues, rendering them incapable of binding either metal ions or c-di-GMP [86]. Notably, LIC_10122 appears to be fully degenerated as an HD-GYP protein (Figure 6 and Table S4). Interestingly, the HD-GYP proteins of *L. interrogans* exhibit unique features not yet described in the literature. LIC_11189, the only protein predicted to be catalytically active, contains an insertion within the HD-GYP domain that probably does not disrupt its folding. Highly conserved polar residues near the c-di-GMP binding pocket suggest a potentially novel interaction mechanism with this cyclic dinucleotide. Similarly, the extended β-sheets in the C-terminal domains of LIC_10138 and LIC_10139 may create a comparable effect despite the absence of canonical c-di-GMP binding residues. The presence of a highly conserved lysine residue in both proteins could implicate interactions through a new unconventional mechanism, potentially compensating for the missing binding residues. Although CDD and InterPro did not annotate LIC_11563 beyond its N-terminal domain, structural similarities suggest that all four proteins may represent a divergent group within the DUF3391 family. DUF3391, an uncharacterized domain commonly found in the N-terminal region of HD-GYP proteins, is structurally solved in *Bdellovibrio bacteriovorus* (PDB 3TM8 [136]). Previous studies revealed that DUF3391 deletion in some HD-GYP proteins did not affect enzymatic activity and the domain does not participate in c-di-GMP binding, leaving its biological role unclear [86,137,138]. Additionally, this work identifies similar previously undescribed domains in the C-terminal regions of LIC_10122, LIC_10138, LIC_10139, and LIC_11563. Structural predictions suggest these regions fold into compact globular domains. Among them, LIC_10122 is the most divergent. This analysis highlights the structural and functional diversity of HD-GYP proteins in *L. interrogans*, expanding our understanding of this enigmatic protein family and suggesting potential novel mechanisms of c-di-GMP interaction beyond canonical binding motifs.

Interestingly, a novel phosphodiesterase named CpdB (bifunctional 2′,3′-cyclic-nucleotide 2′-phosphodiesterase/3′-nucleotidase) has been described that degrades several bacterial-derived cyclic dinucleotides (CDNs) but not the host-derived 2′3′-cGAMP, thereby modulating the innate immune response. Notably, CpdB is itself inhibited by 2′3′-cGAMP and can hydrolyze its own secreted c-di-GMP in vivo. The CpdB is structurally unrelated to previously characterized CDN phosphodiesterases of the metallophosphoesterase superfamily. Homologous proteins have been reported in *E. coli* (CpdB), *Streptococcus suis* (SntA), *Staphylococcus agalactiae* (CdnP), but not in *L. interrogans* [139].

Through bioinformatics and structural biology analyses, we identified twelve proteins containing a PilZ domain in *L. interrogans* (Figure 3 and Figure 7). Among these, LIC_10128 lacks accessory domains but retains critical residues, including arginines at positions 19 and 23 in the N-terminal region, as well as the (D/N)xSxxG motif in the C-terminal region. These features suggest that this PilZ domain is functional and may bind c-di-GMP. In contrast, the other three proteins, LIC_1447, LIC_11993, and LIC_1249, contain a stand-alone PilZ domain that is predicted not to bind c-di-GMP. Similar functional diversity among PilZ-containing proteins has been described in other bacteria. For instance, in *Pseudomonas aeruginosa*, the Alg44 protein interacts with c-di-GMP to activate the alginate secretion complex [140,141]. Likewise, in *Caulobacter crescentus*, the DgrA protein, a PilZ homolog, binds c-di-GMP to regulate the transition from a motile, flagellated cell to an adherent, sessile form—a crucial step in the bacterium’s life cycle [132]. Similarly, in *E. coli*, the YcgR protein, upon binding c-di-GMP (YcgR–c-di-GMP), interacts with MotA and FliG at their interface [142]. This interaction increases resistance and reduces or extinguishes energy transfer between the stator and rotor of the flagellar motor, resulting in decreased or arrested swimming motility [142]. Notably, this motility inhibition occurs only when YcgR interacts with both MotA and FliG; mutations disrupting either interaction site lead to a reduced counterclockwise bias and restoration of swimming speed [143]. Interestingly, *X. campestris* contains an atypical, tetrameric PilZ family (tPilZ), also found in *Campylobacter jejuni*. This tPilZ family lacks the canonical c-di-GMP binding motifs but appears to interact with the flagellar machinery similarly to YcgR [144]. Remarkably, the *Campylobacterota* phylum lacks GGDEF domains and does not produce c-di-GMP [144]. Nevertheless, interaction predictions and Cryo-ET experiments indicate that even non-canonical PilZ domains can bind MotA and potentially interact with FliG, with conserved interaction residues despite the absence of the (D/N)xSxxG motif [144]. It is possible that these non-canonical PilZ domains, stabilized by two additional α-helices between the first and second beta-strands, evolved to function independently of c-di-GMP signaling [144]. Despite the diversity of PilZ domain configurations, we found no evidence for the presence of the tetrameric PilZ family in *L. interrogans*. Neither sequence homology searches nor structural fold analyses of orthologs revealed this family in the *L. interrogans* genome, suggesting that the PilZ domains in this organism are more likely to function through classical c-di-GMP binding rather than through an alternative, c-di-GMP-independent mechanism.

PilZ domains play fundamental roles in controlling gene expression, exemplified by the interaction of c-di-GMP with the MrkH protein in *Klebsiella pneumoniae* [145]. In this regard, MrkH functions as a transcriptional activator, facilitating the association of RNA polymerase with suboptimal promoters that regulate the expression of type III fimbriae [145]. Structurally, MrkH is characterized by a C-terminal PilZ domain, specialized in binding c-di-GMP, and an N-terminal β-barrel domain, similar to other PilZ and YcgRN domains [146]. Notably, the PilZ domain of MrkH contains a positively charged helix that promotes interaction with DNA, indicating that the C-terminal motif plays a crucial role in binding additional partners beyond c-di-GMP [21]. These examples illustrate the structural and functional diversity of non-canonical PilZ domains, enabling bacteria to respond more broadly to c-di-GMP-mediated signals.

The LIC_20173 protein exemplifies functional diversity in *L. interrogans*. It is a membrane protein composed of a non-canonical PilZ domain associated with a PrsW domain, a metalloprotease similar to that found in *Bacillus subtilis* (Figure S17). The PrsW domain of LIC_20173 is predicted to be an active protease based on our analysis (Figure S17). In *Bacillus*, PrsW functions as an independent membrane protease, whereas in *Leptospira*, it is fused to a PilZ domain. Its primary role is likely to cleave the anti-sigma factor RsiW, thereby activating genes involved in stress response and antimicrobial defense [118,147,148]. Extracytoplasmic function (ECF) sigma factors constitute a specialized signaling system that regulates the response to environmental stresses. Under normal conditions, their activity is inhibited by anti-sigma factors, which prevent sigma factor interaction with RNA polymerase. The detection of an external signal triggers intramembrane proteolysis, releasing the sigma factor and promoting the expression of genes associated with cellular adaptation [149,150,151]. LIC_20173 shares similarities with PrsW from *B. subtilis*, whose function is to cleave RsiW, activating genes involved in stress response, including resistance to antimicrobial peptides and cell wall integrity [148]. In *Clostridium difficile*, PrsW plays a similar role [148]. Indeed, mutation studies have shown that deletion of this gene reduces ECF sigma factor expression, increasing sensitivity to antimicrobial peptides and impairing colonization ability, highlighting the importance of this regulatory pathway [148].

Exploring further into the evolution of PilZ domains, there are also truncated PilZ domains, characterized by the presence of a GAF-like domain within their core structure, known as GAZ domain. This insertion results in a unique architecture that differs from canonical PilZ domains. The insertion of the GAZ domain may influence the function of the PilZ domain, altering its interactions and its response to c-di-GMP. Additionally, these proteins feature a third domain in the N-terminal region, PilZN (also known as YcgR_N), which may play an additional role in the functional regulation of these proteins [138]. The *L. interrogans* proteins with this structural configuration (YcgR^GAZ^) include LIC_10049, LIC_11920, LIC_12546, LIC_12723, LIC_12994, and LIC_14002. Due to their distinct architecture, these proteins have been annotated in the database as belonging to the DUF1577 domain. This classification reflects the structural and functional uniqueness of the truncated PilZ domains that contain an inserted GAF-like domain. Although the exact function of these proteins is not yet fully elucidated, their structure suggests a potential role in cellular signaling and adaptation to different environmental conditions. However, upon analyzing the sequences of proteins annotated as DUF1577, the presence of the conserved motifs RxxxR and (D/N)xSxxG is evident. This conservation suggests that, despite the insertion of the GAF-like domain, these proteins still have the potential to interact with c-di-GMP. Our recent study showed that LIC_11920, one of the proteins annotated as DUF1577, is capable of interacting with the c-di-GMP molecule [20]. This interaction occurs through the RxxxR motif. Another important finding in this study is that the GAF-like domain of LIC_11920 completely loses its dimerization capability [20]. This limitation is attributed to the absence of an α-helix in the GAF-like domain of LIC_11920. The absence of this essential structure prevents dimer formation. This observation underscores the evolutionary implications of structural modifications in GAF-like domains and their consequences for the biology of proteins associated with c-di-GMP [20]. Analysis of the genomic context of genes encoding the PilZ domain–containing proteins did not provide sufficient evidence to infer their possible functional roles. Therefore, experimental approaches will be essential to elucidate their biological functions in *Leptospira* species. The abundance of proteins containing PilZ domains in *L. interrogans* highlights the complexity of the c-di-GMP signaling network in this bacterium and the importance to investigate the biological function of each c-di-GMP circuit.

**Figure 10:**
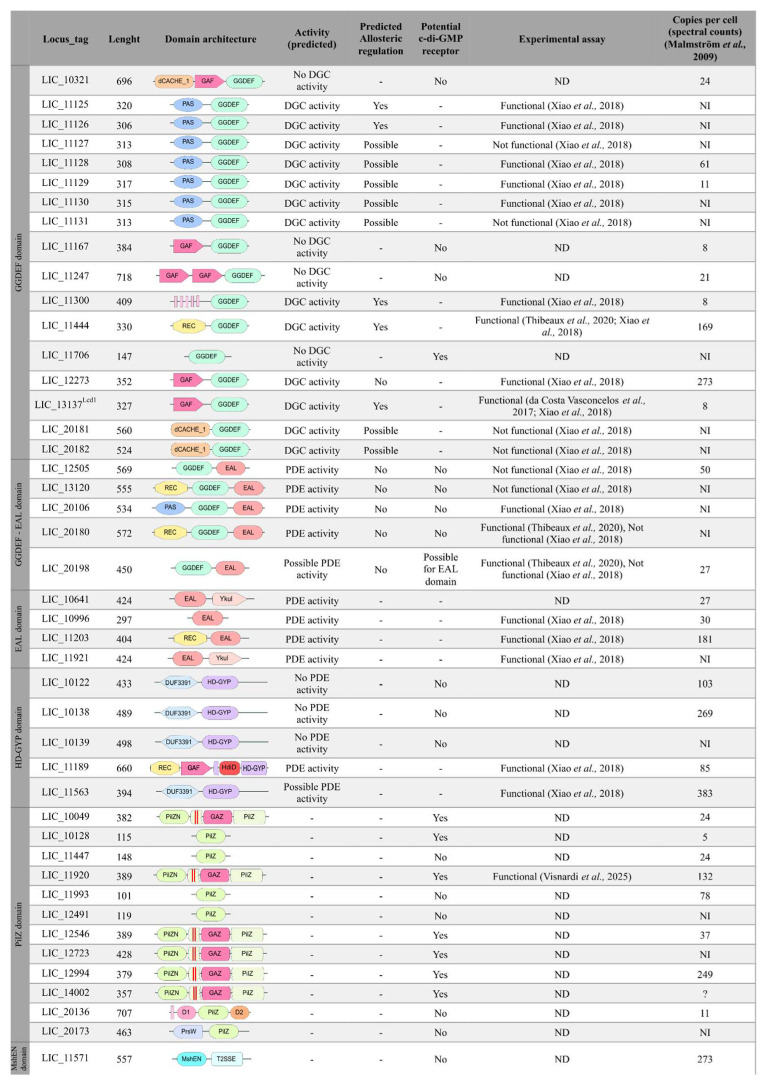
Number of proteins containing GGDEF, EAL, HD-GYP, or PilZ domains in *L. interrogans* serovar Copenhageni Fiocruz L1-130. Experimental evidence was obtained by several authors, in different ways: From recombinant proteins and heterologous expression in *E. coli* [17,18,20] or by producing knockouts in *L. interrogans* [16]. For proteins containing both GGDEF and EAL domains, only the predicted PDE activity was tested, as all of them possess a degenerate GGDEF domain [18]. Data of copies per cell was from a proteome of the cell sample subjected to extensive mapping via LC-MS/MS experiments [160]. (-) means that this functional is not applied to this protein; (?) we could not identify the protein in the list of proteins provided by the article. (ND) no data was found for this protein. (NI) not identified. The gene with RefSeqGene LIC_14002 or LIC_RS11585 is not annotated in *L. interrogans* serovar Copenhageni strain Fiocruz L1-130 genome, and this gene is orthologous to LA_1489.

Figure 10 summarizes the enzymes described in this study, integrating our activity predictions with the experimental assays previously reported by Xiao et al. and Thibeaux et al. for most DGCs and PDEs [16,18]. Based on our comparative analysis, which integrates bioinformatics data generated in this study with information previously reported in the literature, it becomes evident that most c-di-GMP receptors have not yet been experimentally characterized, and that not all genes predicted to encode DGC or PDE activities have been tested, including LIC_10321, LIC_11167, LIC_11247, LIC_11706, LIC_10641, LIC_10122, LIC_10138, and LIC_10139. For the gene LIC_11706, we predict the absence of DGC enzymatic activity, however, we propose that the protein may function as a c-di-GMP receptor, since it retains an intact allosteric binding site.

Our predictions for DGCs and PDEs proteins are consistent with experimental data in 17 cases out of 23 proteins experimentally tested. We identified six discrepant proteins (LIC_11127, LIC_11131, LIC_20181, LIC_20182, LIC_12505, and LIC_13120) that we predicted to be enzymatically active whose activity was not detected experimentally. In these cases, we hypothesize that enzymatic activity might be modulated by the protein’s effector domain, a factor that was not addressed in the studies that evaluated their enzymatic properties [18]. Furthermore, it is noteworthy that only one study, focused on Lcd1 [17], has directly investigated the allosteric regulation of a DGC and identified the effector ligand acting through the sensory domain to modulate the activity of the GGDEF domain [17]. Since most DGCs are thought to be regulated both by product feedback and by their associated sensory domains, this underscores a significant gap in our understanding of their regulatory mechanisms. Overall, our analysis supports the majority of experimental data available in the literature but also uncovers critical gaps that require further biophysical investigations to better elucidate the regulatory mechanisms and c-di-GMP signaling pathways in this medically relevant spirochete.

Interestingly, in Lic all proteins detected to contain both GGDEF and EAL domains the GGDEF domain is degenerated [18] (Figure 10). In the literature, hybrid enzymes containing both GGDEF and EAL domains can exhibit a variety of functional states: only one may be active, or both may be degenerated [22,79,129,152], or both domains may be active, rendering the protein bifunctional [152,153,154]. Typically, even when both domains are conserved, these proteins exhibit a predominant catalytic activity, with only one domain being active under specific conditions. The predominant activity is often modulated by self-produced or external signals [155,156,157]. For example, the CdgB protein from *Azospirillum baldaniorum* is a bifunctional enzyme in which either DGC or PDE activity is observed, depending on the conditions under which the protein is expressed [158]. Such differences may arise from species-specific signals or from the experimental conditions used during the assays [158]. Another example well-documented is the FimX protein from *Xanthomonas citri* pv. citri, where the GGDEF and the EAL domains are degenerated but the EAL domain binds c-di-GMP and modulates twitching motility [159]. In *Leptospira interrogans*, however, the reasons why hybrid enzymes appear to retain only EAL activity remain unclear.

**Figure 11:**
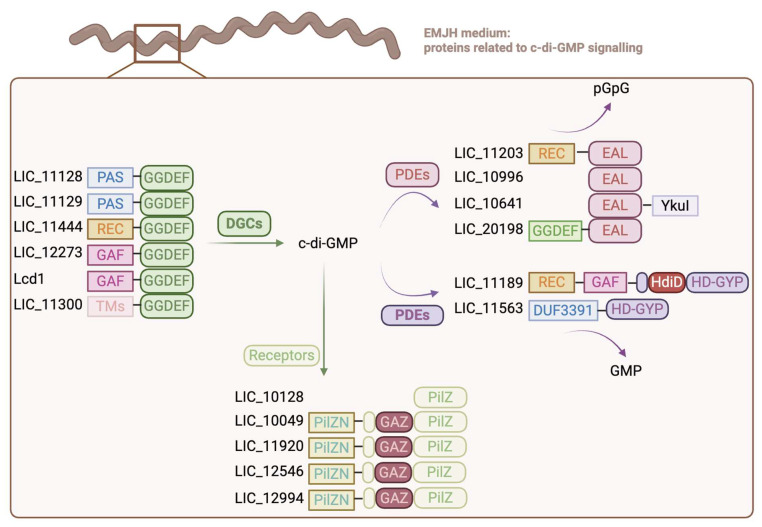
Number of proteins containing active GGDEF, EAL, or PilZ domains in *L. interrogans* serovar Copenhageni strain Fiocruz L1-130 when grown in EMJH medium, based on previously published mass spectrometry data [160]. The illustration was created using BioRender (Guzzo, C. (2026) https://BioRender.com/z8me4hv, accessed on 1 December 2025) and summarizes the results presented in Figure 10, which integrates our activity predictions with previously reported experimental assays [16,17,18,20].

Interestingly, *L. interrogans* cultured in EMJH medium expresses a diverse repertoire of c-di-GMP signaling components, including six proteins with GGDEF domains and seven PDEs (five with EAL domains and two with HD-GYP domains), and five predicted c-di-GMP receptors containing PilZ domains [160]. Finally, integrating our predictions with previously published experimental data, we summarized the number of *L. interrogans* serovar Copenhageni strain FioCruz L1-130 proteins containing active GGDEF, EAL, or PilZ domains that are present when the bacterium is grown in EMJH medium, based on mass spectrometry evidence [160]. This overview, illustrated in Figure 11, provides a global perspective on the subset of c-di-GMP signaling components under laboratory conditions, reinforcing the dynamic nature and functional relevance of this regulatory network in *L. interrogans* physiology. This complex system underscores the remarkable regulatory potential of c-di-GMP signaling in *L. interrogans* physiology and pathogenicity. Future studies should aim to elucidate the regulatory mechanisms governing the expression of these genes, as well as to identify the specific signals sensed by the regulatory domains that precede DGC and PDE catalytic modules. Moreover, mapping the network of protein–protein interactions and signal transduction pathways underlying this system will be essential to understand how c-di-GMP signaling integrates environmental and host-derived cues. Finally, the discovery and characterization of novel c-di-GMP receptors not explored in this work will provide key insights into how *L. interrogans* coordinates motility, biofilm formation, and virulence through this intricate second messenger network.

## 5. Conclusions

This study provides a comprehensive analysis of the c-di-GMP signaling network in *L. interrogans*, revealing an intricate regulatory system composed of potentially 44 proteins involved in cyclic di-GMP metabolism, including diguanylate cyclases (DGCs), phosphodiesterases (PDEs), and c-di-GMP-binding effectors. Our findings highlight the remarkable c-di-GMP signaling capacity of *L. interrogans*, with a high turnover domain density (IQ of 9.4 per Mbp), exceeding the bacterial average. This suggests that c-di-GMP plays a pivotal role in bacterial adaptation, biofilm formation, and survival under environmental stressors. Through structural homology and sequence conservation analyses, we identified 22 putative DGCs, of which 13 are predicted to be enzymatically active, and nine have been previously confirmed to exhibit diguanylate cyclase activity [16,18]. The observed inconsistencies in LIC_11127, LIC_11131, LIC_20181, and LIC_20182 likely reflect the modulatory effects of associated effector or regulatory domains on enzyme activation by ligands, emphasizing the need for comprehensive experimental analyses to clarify the functional roles of these proteins. We also identified one degenerate GGDEF domain (LIC_11706), while lacking catalytic activity, may still function as a c-di-GMP receptor.

Similarly, among the nine identified EAL domain-containing proteins, eight retain the conserved residues essential for PDE activity and one is predicted to have a possible PDE activity. Notably, five of these proteins also contain a GGDEF domain; however, only the EAL domain is predicted to be enzymatically active. Previous experimental assays have been performed for eight of these proteins, with consistent PDE activity observed for six, while two (LIC_12505 and LIC_13120) were reported to be inactive [16,18]. This inconsistency is likely due to the influence of associated effector or regulatory domains on enzyme activation, highlighting the need for more detailed experimental studies to elucidate the functionality of these proteins. Further biochemical and structural analyses will be required to clarify the mechanisms governing the activity and regulation of this group of proteins. We identified five proteins containing HD-GYP domains that exhibit a striking degree of structural diversity, with only one catalytically active candidate (LIC_11189). One of them, LIC_11563, may have a possible PDE activity. The experimental PDE activity of them was previously confirmed by Xiao and collaborators [18]. In this study, we identified a novel insertion domain within an HD-GYP protein, designated here as HdiD (HD-GYP insertion domain), found in LIC_11189. This domain may participate in c-di-GMP binding and appears to be widely distributed among HD-GYP proteins across different bacterial phyla. We also identified an unannotated C-terminal domain in the proteins LIC_10122, LIC_10138, LIC_10139, and LIC_11563. However, due to the high degree of sequence divergence, we were unable to characterize these domains, underscoring the need for further in-depth analyses to elucidate their structure and function.

The identification of 12 PilZ domain-containing proteins suggests multiple regulatory roles for c-di-GMP in *L. interrogans*, likely influencing motility, biofilm formation, and gene expression. Our analysis revealed that seven out of 12 are predicted to be a c-di-GMP receptor. Of the 12 proteins containing a PilZ domain, only one (LIC_11920) has been previously characterized by our group, where we demonstrated its ability to bind c-di-GMP; however, its biological function remains to be elucidated [20]. Our analyses reveal that LIC_20136 and LIC_20173 represent two previously uncharacterized PilZ-like proteins with distinctive domain architectures that may define novel functional families within *Leptospira*. For LIC_20136, we describe and designate two unique regions—the D1 and D2 domains—identified here for the first time, with the D1 domain resembling an RNA-binding four-helix bundle–like structure and the D2 domain showing structural similarity to ATLF domains yet lacking catalytic residues, suggesting a role in protein–protein interactions rather than proteolysis. In contrast, LIC_20173 combines a predicted active PrsW protease domain with a C-terminal PilZ domain, forming an intriguing transmembrane regulatory module likely anchored to the inner membrane. The fusion of protease and PilZ domains in LIC_20173 and the presence of an ATLF-like domain in LIC_20136 highlight the evolutionary diversification of PilZ-containing signaling proteins in *Leptospira*.

Our analysis shows that the c-di-GMP signaling network of *L. interrogans* is largely conserved, with most genes forming part of the species’ core genome. However, lineage-specific gene losses—such as those affecting LIC_11447, LIC_11921, and LIC_11131—indicate evolutionary diversification among serovars. These variations suggest that while the core regulatory framework is maintained, peripheral signaling components undergo adaptive changes that may influence the physiology and virulence of distinct *L. interrogans* lineages.

Altogether, this study expands our understanding of the c-di-GMP regulatory network in *L. interrogans*, providing insights into the functional complexity of this second messenger system. However, experimental validation is essential to confirm the enzymatic activities, regulatory interactions, and physiological relevance of these proteins. Future studies integrating biochemical, genetic, and structural approaches will be crucial to elucidate the full scope of c-di-GMP-mediated regulation in *L. interrogans* and its implications for pathogenesis and environmental persistence. In summary, our study provides a comprehensive overview of the repertoire of proteins in *L. interrogans* potentially involved in c-di-GMP turnover and binding. The novelty and relevance of our findings lie in the identification of previously uncharacterized proteins, including two novel PilZ domain proteins (LIC_11993 and LIC_20136), four proteins with GGDEF domains (LIC_10321, LIC_11167, LIC_11247, and LIC_11706), one with an HD-GYP domain (LIC_10122), and two proteins containing EAL domains without a previously described GGDEF domain (LIC_20106 and LIC_20198). We further annotated the presence of a dCACHE_1 domain in LIC_20181 and DUF3391 domains in LIC_10122, LIC_10138, and LIC_10139. Importantly, we identify a novel insertion domain within the HD-GYP protein family, here designated HdiD, which is present in the only enzymatically active protein, LIC_11189. We also describe novel domains, here named D1 and D2 domains in LIC_20136, in association with a PilZ domain. In addition, our analysis highlights misannotations in the UniProt database, including incorrect domain assignments and wrongly defined start codons, as exemplified by LIC_12273. Altogether, these findings expand the current knowledge of c-di-GMP signaling components in *L. interrogans*, providing a valuable framework for future functional and structural investigations.

## Figures and Tables

**Table 1:** Number of genomes of *L. interrogans* isolates with orthologs of 27 core c-di-GMP-related genes. Locus names refer to the original annotation of *L. interrogans* serovar Copenhageni strain Fiocruz L1-130. Only genes present in more than 99% of the isolates are considered part of *L. interrogans* core genome.

Locus	Genomes	Percentage
LIC_10049	364	99.73%
LIC_10128	365	100%
LIC_10139	362	99.18%
LIC_10321	365	100%
LIC_10996	364	99.73%
LIC_11125	362	99.18%
LIC_11127	362	99.18%
LIC_11129	362	99.18%
LIC_11130	363	99.45%
LIC_11167	364	99.73%
LIC_11247	362	99.18%
LIC_12273	364	99.73%
LIC_11300	362	99.18%
LIC_11444	365	100%
LIC_11563	363	99.45%
LIC_11571	364	99.73%
LIC_11706	365	100%
LIC_11993	365	100%
LIC_12994	364	99.73%
LIC_13137	363	99.45%
LIC_12491	363	99.45%
LIC_12723	363	99.45%
LIC_12546	364	99.73%
LIC_14002	365	100%
LIC_20173	362	99.18%
LIC_20198	364	99.73%
LIC_20182	363	99.45%

## Data Availability

The data presented in this study are openly available in Preprints at https://doi.org/10.20944/preprints202505.0068.v1.

## Supplementary Materials

The following supporting information can be downloaded at: https://www.mdpi.com/article/10.3390/pathogens15020151/s1, Figure S1. A dendrogram with heatmap showing the results of a DALI all-against-all structure comparison of GGDEF, EAL, HD-GYP, and PilZ domains containing-proteins from *L. interrogans*; Figure S2. Domain architecture of HD-GYP domain containing-proteins from *L. interrogans*; Figure S3. Secondary structure topology of the HD-GYP domains; Figure S4. Distribution and domain architecture of proteins containing the insertion from LIC_11189 HD-GYP domain; Figure S5. Residue conservation analysis of the HdiD (HD-GYP insertion domain) in LIC_11189; Figure S6. Topology comparison of annotated DUF3391 and N-terminal domains from *L. interrogans* HD-GYP proteins; Figure S7. Sequence alignment and phylogeny of the N-terminal domains; Figure S8. Predicted structure and topology analysis of the C-terminal domains from *L. interrogans* HD-GYP proteins; Figure S9. Structural comparison of LIC_10138_S371-A489_ and its Dali and FoldSeek matches; Figure S10. Structural comparison of LIC_101398S371-A489_L383-A503_ with mitochondrial ribosome subunit and its FoldSeek best match; Figure S11. Residue conservation analysis of LIC_11563_L326-A394_; Figure S12. Residue conservation analysis of LIC_10138_S371-A489_; Figure S13. Residue conservation analysis of LIC_10139_L383-A503_; Figure S14. Charges of the side chains of amino acids inward the barrel-like structure of LIC_11563_L326-A394_, LIC_10138_S371-A489_, and LIC_10139_L383-A503_; Figure S15. Participation of conserved residues in LIC_10138_S371-A489_, and LIC_10139_L383-A503_ in the interaction among the HD-GYP and the C-terminal domain; Figure S16. LIC_20136 contains a Transmembrane and D1, PilZ and D2 Domains; Figure S17. Structure and amino acid sequence alignment of the PrsW domain of the YpdC protein from *B. subtilis* and LIC_20173 from *L. interrogans* serovar Copenhageni strain Fiocruz L1-130; Figure S18. Rarefaction curve of the *L. interrogans* pangenome; Figure S19. Genome-based phylogeny of all 365 *L. interrogans* isolates; Table S1. Keywords to search proteins related to c-di-GMP in the Uniprot database; Table S2. Table with the proteins identified by Blastp in *L. interrogans* serovar Copenhageni strain Fiocruz L1-130, which may be related to c-di-GMP signaling; Table S3: Sensor and signaling domains found in proteins associated with c-di-GMP signaling in the *L. interrogans* genome; Table S4: Functions of the key residues in the HD-GYP domain, using as a model of the protein of *Persephonella marina* (locus_tag PERMA_0986); Table S5: Corrected sequences of proteins used in this work based on the sequence of RefSeq proteins with the name of old locus_tag and the locus_tag of RefSeq are below; Table S6. Orthologs proteins presents in the genomes of *L. interrogans* serovar Lai strain 56601 (Taxonomy ID: 189518), *L. borgpertersenii* serovar Hardjo-bovis strain JB197 (Taxonomy ID: 355277), and *L. biflexa* serovar Patoc strain Patoc 1 (Paris) (Taxonomy ID: 456481).
