# Supplementary material for: Discovery of Novel c-di-GMP-Related Genes in Leptospira interrogans
Source: Pathogens. 2026 Jan 30;15(2):151. doi: 10.3390/pathogens15020151 (PMC12943098; doi:10.3390/pathogens15020151)
Supplement: Supplementary file 1 [file pathogens-15-00151-s001.zip › Supplementary Figures and Tables.pdf]

Supplementary Materials

Contents

Supplementary text

Potential Distant Members of DUF3391 Family:

A novel C-terminal Domain in HD-GYP-containing Proteins

Figure S1. A dendrogram with heatmap showing the results of a DALI all-against-all structure comparison of GGDEF, EAL, HD-GYP, and PilZ domains containing-proteins from *L. interrogans*.

Figure S2. Domain architecture of HD-GYP domain containing-proteins from *L. interrogans*

Figure S3. Secondary structure topology of the HD-GYP domains.

Figure S4. Distribution and domain architecture of proteins containing the insertion from LIC_11189 HD-GYP domain.

Figure S5. Residue conservation analysis of the HdiD (HD-GYP insertion domain) in LIC_11189.

Figure S6. Topology comparison of annotated DUF3391 and N-terminal domains from *L. interrogans* HD-GYP proteins.

Figure S7. Sequence alignment and phylogeny of the N-terminal domains.

Figure S8. Predicted structure and topology analysis of the C-terminal domains from *L. interrogans* HD-GYP proteins.

Figure S9. Structural comparison of LIC_10138S371-A489 and its Dali and FoldSeek matches.

Figure S10. Structural comparison of LIC_101398S371-A489L383-A503 with mitochondrial ribosome subunit and its FoldSeek best match.

Figure S11. Residue conservation analysis of LIC_11563L326-A394.

Figure S12. Residue conservation analysis of LIC_10138S371-A489.

Figure S13. Residue conservation analysis of LIC_10139L383-A503.

Figure S14. Charges of the side chains of amino acids inward the barrel-like structure of LIC_11563L326-A394, LIC_10138S371-A489, and LIC_10139L383-A503.

Figure S15. Participation of conserved residues in LIC_10138S371-A489, and LIC_10139L383-A503 in the interaction among the HD-GYP and the C-terminal domain.

Figure S16. LIC_20136 contains a Transmembrane and D1, PilZ and D2 Domains.

Figure S17. Structure and amino acid sequence alignment of the PrsW domain of the YpdC protein from B. subtilis and LIC_20173 from *L. interrogans* serovar Copenhageni strain Fiocruz L1-130.

Figure S19. Genome-based phylogeny of all 365 *L. interrogans* isolates.

Table S1 – Keywords to search proteins related to c-di-GMP in the Uniprot database.

Table S2 - Table with the proteins identified by Blastp in *L. interrogans* serovar Copenhageni strain Fiocruz L1-130, which may be related to c-di-GMP signaling.

Table S3 - Sensor and signaling domains found in proteins associated with c-di-GMP signaling in the *L. interrogans* genome.

Table S4 - Functions of the key residues in the HD-GYP domain, using as a model of the protein of Persephonella marina (locus_tag PERMA_0986).

Table S5 - Corrected sequences of proteins used in this work based on the sequence of RefSeq proteins with the name of old locus_tag and the locus_tag of RefSeq.

Table S6 - Orthologs proteins presents in the genomes of *L. interrogan*s serovar Lai strain 56601 (Taxonomy ID: 189518), *L. borgpertersenii* serovar Hardjo - bovis strain JB197 (Taxonomy ID: 355277), and, *L. biflexa* serovar Patoc strain Patoc 1 (Paris) (Taxonomy ID: 456481).

References

Supplementary text

Potential Distant Members of DUF3391 Family:

BLASTp searches [1] using LIC_11563 (residues M1–V131) identified proteins annotated with DUF3391, with high scores (probability 1.0; e-values from $1.3x10^{-18}$ to $5.7x10^{-3}$). When the full-length LIC_11563 was used as a query, the PA4108 HD-GYP protein from *Pseudomonas aeruginosa* PAO1, which also contains a DUF3391 domain, returned with a probability of 1.0 and an e-value of $4.3x10^{-20}$ [2]. For LIC_10122, LIC_10138, and LIC_10139, matches similarly corresponded to N-terminal regions of HD-GYP-containing proteins, although most lacked explicit annotations. Among the matches for LIC_10122, COS84_03205 (from *Armatimonadetes bacterium* CG07_land_8_20_14_0_80_40_9, Taxonomy ID: 1973916) [3] encoded a standalone DUF3391 protein (not fused to an HD-GYP domain) with moderate similarity (probability 0.97; e-value $9.0x10^{-2}$). A subsequent FoldSeek AFDB50 search identified G3N55_00320 (from Dissulfurirhabdus thermomarina strain DSM 100025, Taxonomy ID: 1765737) [4] — another standalone DUF3391 protein — with a higher similarity score (probability of 1.0 and e-value of $8.6x10^{-8}$). LIC_10138 matched to E4G96_08570 from a *Chrysiogenales bacterium* (metagenomic data, Taxonomy ID: 2528039), containing a DUF3391 domain with lower similarity scores (probability=0.69 and e-value = $1.7x10^{-1}$). Using this sequence as a query, FE240_05590 from *Aeromonas simiae [5]*, containing a DUF3391 domain, returned with high confidence (probability of 1.0). Due to the strong sequence and structural similarity between LIC_10138 and LIC_10139, FoldSeek results for LIC_10139 were nearly identical to those for LIC_10138, including matches to some DUF3391 domains. Topology representations of LIC_10122, LIC_10138, LIC_10139, and LIC_11563, alongside PA4108, Dvul_2450 (from *Nitratidesulfovibrio vulgaris* strain DP4 (Taxonomy ID: 391774), FE240_05590, F0M16_21925, and G3N55_00320 as DUF3391 representatives, were generated using PDBsum [6]. Owing to the low confidence of AlphaFold-predicted models, topology analysis of the *L. interrogans* domains was limited.

A novel C-terminal Domain in HD-GYP-containing Proteins

Dali PDB90 and Foldseek PDB100 searches using $LIC\_10122_{E325\text{-}K433}$ as query returned only matches with low similarity ($Z < 3.5$ for DALI and probability of 0.1 in Foldseek). Foldseek AFDB50 analysis identified 25 proteins, 12 with significant scores (probability = 1.0, e-value between $1.7x10^{-18}$ and $3.9x10^{-4}$), including hypothetical proteins

from *Leptospira* species and one HD-GYP protein from *Turneriella parva* DSM 21527 (probability = 1.0, e-value = $2.8x10^{-6}$). Consistent with Foldseek, BLASTp analysis revealed homologs only in closely related *Leptospira* species, including *L. interrogans*, *L. kirschneri*, *L. noguchii*, *L. weilii*, and *L. santarosai* (100% query coverage, 95.4–100% identity) limiting the residue conservation analysis (**data not shown**).

Dali PDB90 analysis of $LIC\_11563_{L326-A394}$ returned structural matches with *Pyrococcus furiosus* transcription elongation factor Spt4/5 (PDB 3P8B [7]), human TDRD1 extended Tudor domain (PDB 5M9N, unpublished article) [8], and *Saccharomyces cerevisiae* Ski238 complex (PDB 8Q9T [9]) with z-score ranging from 5.8 to 5.6 and $RMSD_{C\alpha}$ values ranging from 2.2 to 2.4 Å. Foldseek PDB50 analysis identified matches with the human nucleolar pre-60S ribosomal subunit (PDB 8FKR [10]) and *Nanoarchaeum equitans* ATP synthase core complex (PDB 5BN5 [11]) (both with probability of 0.6 and e-value of $7.3x10^{-1}$ and $3.7x10^{-1}$, respectively). For $LIC\_10138_{S371-A489}$ and $LIC\_10139_{L383-A503}$, DALI analysis returned human SGF29 in complex with R2AK4me3 (PDB 3MEV [12]) and a hypothetical protein from an uncultured marine organism (PDB 3BY7 [13]) as top results (z-score = 5.3, RMSD = 3.1 Å). $LIC\_10139_{L383-A503}$ yielded identical DALI matches to $LIC\_10138_{S371-A489}$, reflecting structural similarity. Foldseek PDB50 analysis for $LIC\_10139_{L383-A503}$ identified the mitochondrial ribosome from *Polytomella magna* (PDB 8APO [14]) as the best hit (probability = 0.84, e-value = $6.8x10^{-2}$), a match also identified for $LIC\_10138_{S371-A489}$, although with lower confidence (probability = 0.41, e-value of $1.4x10^{-1}$) (**Figures S9** and **S10**). Despite structural similarities, sequence alignments with these homologs were poor, likely due to the generic barrel-like architecture of the domain, which may contribute to false-positive homolog identification and moderate confidence scores.

Homology searches for $LIC\_11563_{L326-A394}$, $LIC\_10138_{S371-A489}$, and $LIC\_10139_{L383-A503}$ identified matches exclusively with the C-terminal regions of HD-GYP proteins from diverse bacterial species beyond the *Spirochaetota* phylum. BLASTp analysis of $LIC\_11563_{L326-A394}$ revealed numerous HD-GYP proteins containing this C-terminal domain, including sequences outside the *Spirochaetota* phylum. Divergent sequences were manually curated, aligned using Clustal, and analyzed for residue conservation with WebLogo. Highly conserved residues were identified, with prolines (P328, P351, P354) likely disrupting secondary structures and forming flexible loops, while inward-facing apolar residues (I333, L335, V343, L375, I383) stabilized β-sheet folding through hydrophobic interactions. Conserved surface-exposed residues (R353, R357, K360, K377) formed stabilizing

intradomain hydrogen bonds (R353, K360) or remained solvent-accessible, suggesting roles in protein–protein interactions or ligand binding (**Figure S11**).

BLASTp analysis of LIC_10138$_{S371\text{-}A489}$ and LIC_10139$_{L383\text{-}A503}$ identified homologs exclusively within the *Leptospira* genus. Forty representative hits were selected, aligned, and filtered for redundancy (95% threshold). Residue conservation analysis revealed no strong correlation with LIC_11563$_{L326\text{-}A394}$, aside from conserved R-P-X-X-R and R-P-X-X-X-R motifs in LIC_11563$_{L326\text{-}A394}$ and LIC_10138/LIC_10139, respectively (**Figures S11**, **S12**, and **S13**). All three proteins contained five β-strands with inward-facing apolar side chains, inducing curvature and forming a hydrophobic pocket within the incomplete barrel-like structure (**Figure S14**). Unlike LIC_10122$_{E325\text{-}K433}$, which features a long N-terminal helix separating it from the HD-GYP domain, LIC_11563$_{L326\text{-}A394}$, LIC_10138$_{S371\text{-}A489}$, and LIC_10139$_{L383\text{-}A503}$ are in close proximity to their HD-GYP domains. LIC_10138$_{S371\text{-}A489}$ and LIC_10139$_{L383\text{-}A503}$ also possess longer β4 and β5 strands near the degenerated c-di-GMP binding pocket (**Figure S2**). Absolutely conserved residues, such as K430 in LIC_10138 and K443 in LIC_10139, mediate interactions with the HD-GYP domain via hydrogen bonding with E325 and E338, respectively. These glutamic acid residues are oriented toward the c-di-GMP binding pocket, suggesting a potential role in nucleotide interaction, analogous to that proposed for LIC_11189. Additionally, a hydrophobic pocket along the extended β-sheets mediates apolar interactions with the HD-GYP domain, with absolute conservation of interacting residues suggesting their importance for structural integrity (**Figure S15**).

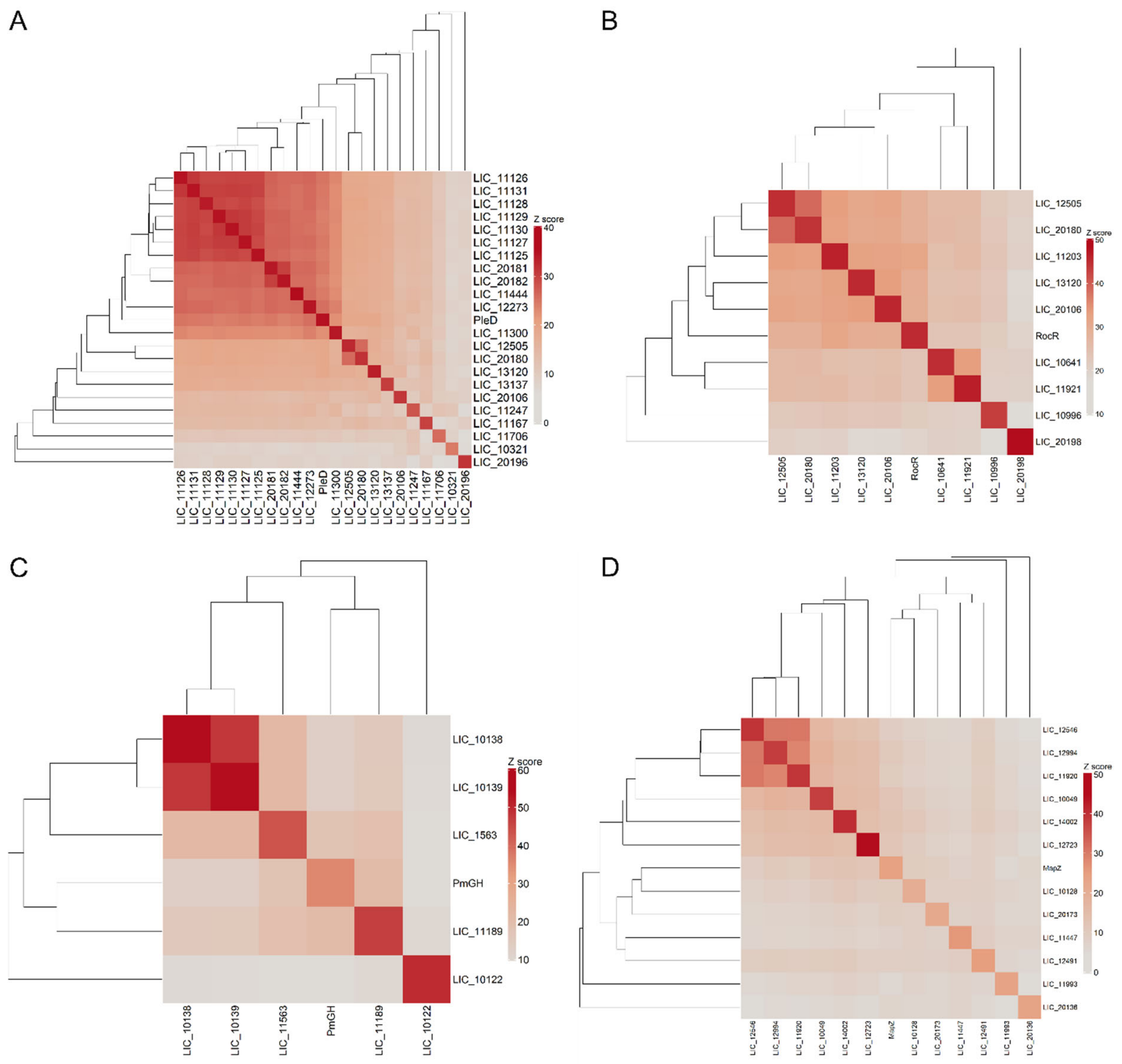

Figure S1. A dendrogram with heatmap showing the results of a DALI all-against-all structure comparison of GGDEF, EAL, HD-GYP, and PilZ domains containing-proteins from *L. interrogans*.

(A) Proteins containing GGDEF domain. (B) Proteins containing EAL domain. (C) Proteins containing HD-GYP domain. (D) Proteins containing the PilZ domain. The colours are based on the DALI Z-score [15]. The figure was made using R software (version 4.4.1) [16] with the packages ComplexHeatmap [17], and ape 5.0 [18].

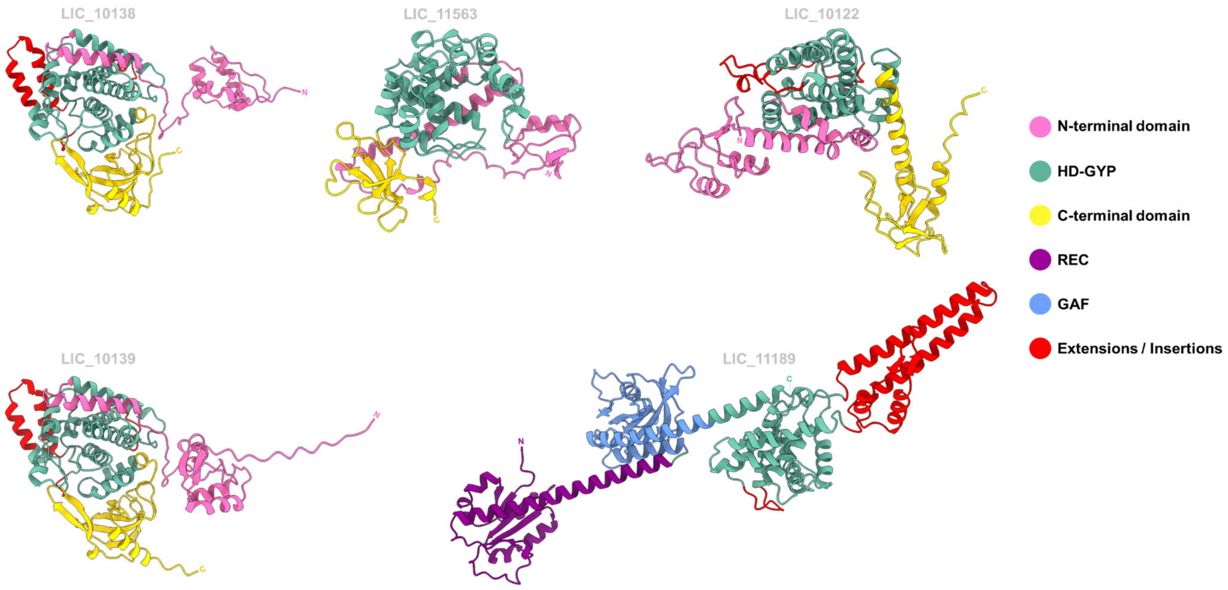

Figure S2. Domain architecture of HD-GYP domain containing-proteins from *L. interrogans*.

The HD-GYP domain is colored in green and extensions/insertions in red compared to PmGH structure (PDB 4MDZ [19]). Conserved N and C-terminal domains are colored in hot pink and yellow, respectively. For LIC_11189, REC and GAF domains are colored in purple and blue, respectively. Cartoon representation of HD-GYP structures colored in ChimeraX version 1.9 [20].

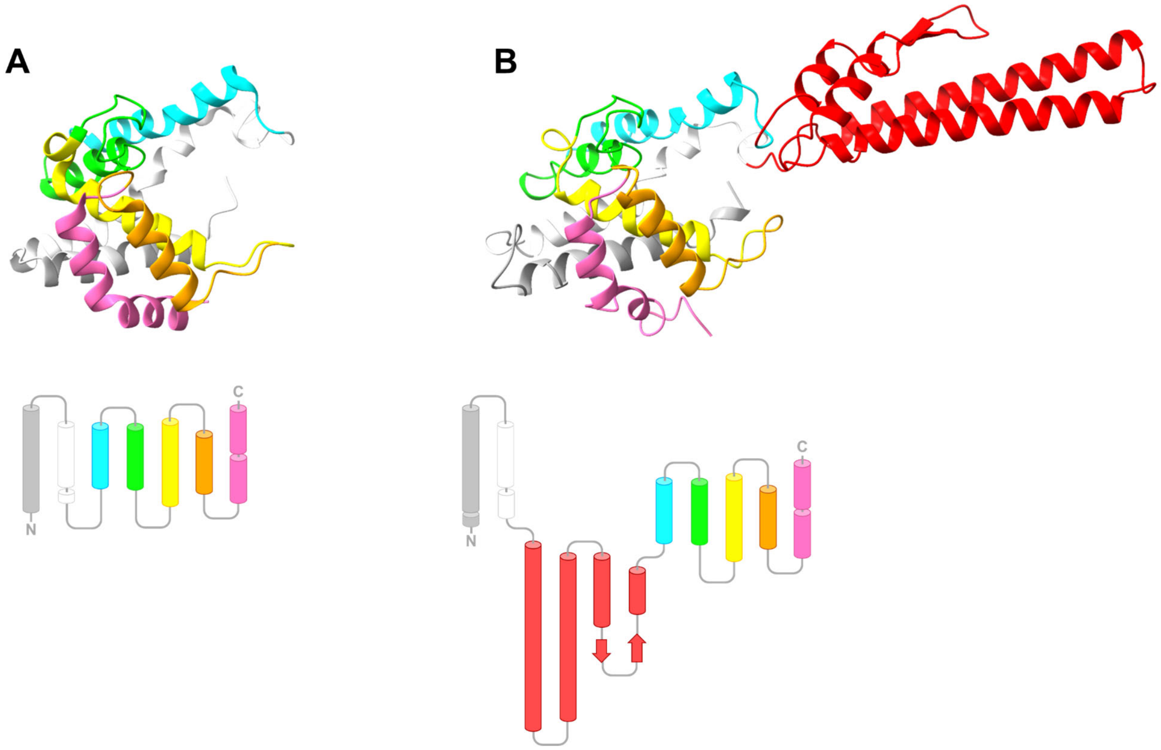

Figure S3. Secondary structure topology of the HD-GYP domains.

PmGH structure (PDB 4MDZ) (**A**) and LIC_11189 (**B**). Cartoon representation of HD-GYP structures colored in ChimeraX version 1.9 [20]. Secondary structure topology figures were manually adapted from the topology map generated by PDBsum software [6] using the crystal structure of PmGH and the Alphafold model of LIC_11189. The LIC_11189 insertion of a subdomain comprising two long helices, two short helices, and one hairpin in the middle of the domain is colored in red while the HD-GYP domain is colored in other colors.

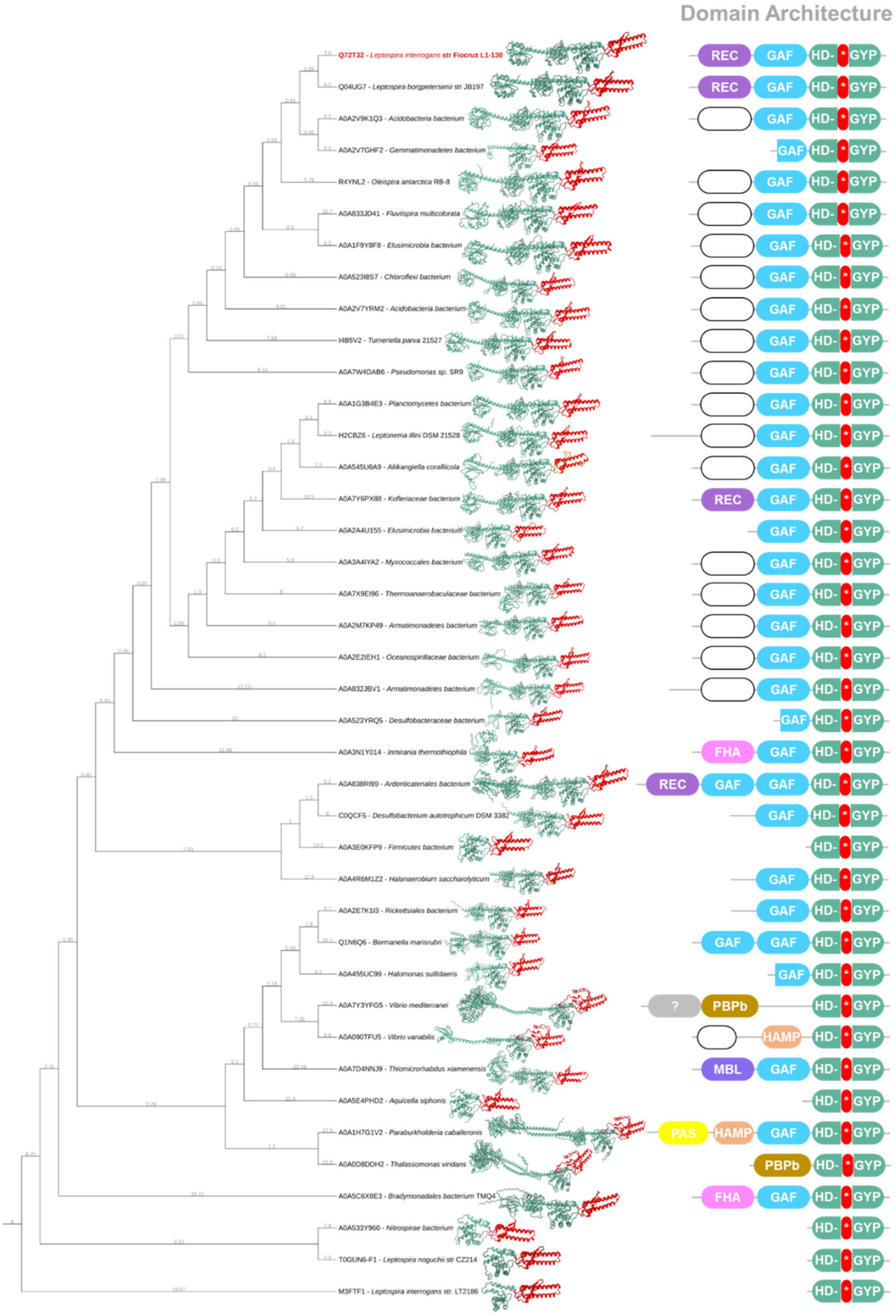

Figure S4. Distribution and domain architecture of proteins containing the insertion from LIC_11189 HD-GYP domain.

Matches from a FoldSeek AFDB50 search [21] (probability = 1.0) using the LIC_11189$_{K416-D536}$ region were manually selected to construct a dendrogram in the DALI server based on structural similarity (left). AlphaFold3 [22] codes for each predicted structure, depicted in

green cartoon representation, are shown alongside the bacterial species names. The HD-GYP insertion is highlighted in red. LIC_11189$_{FL}$ is highlighted in dark red. Branches length are written in gray. Domain annotations were obtained using InterPro [23] and the Conserved Domain Database (CDD) [24], with the following color scheme: HD-GYP domains in green, the insertion (*) in red, GAF domains in blue, phosphoacceptor receiver (REC) domains in purple, forkhead-associated (FHA) domains in pink, penicillin-binding protein (PBPb) domains in brown, HAMP dimerization domains in orange, PAS domains in yellow, structurally similar REC-like domains in white, and domains with no similarity to known proteins in gray (?). Cartoon representation of HD-GYP structures colored in ChimeraX version 1.9 [20]

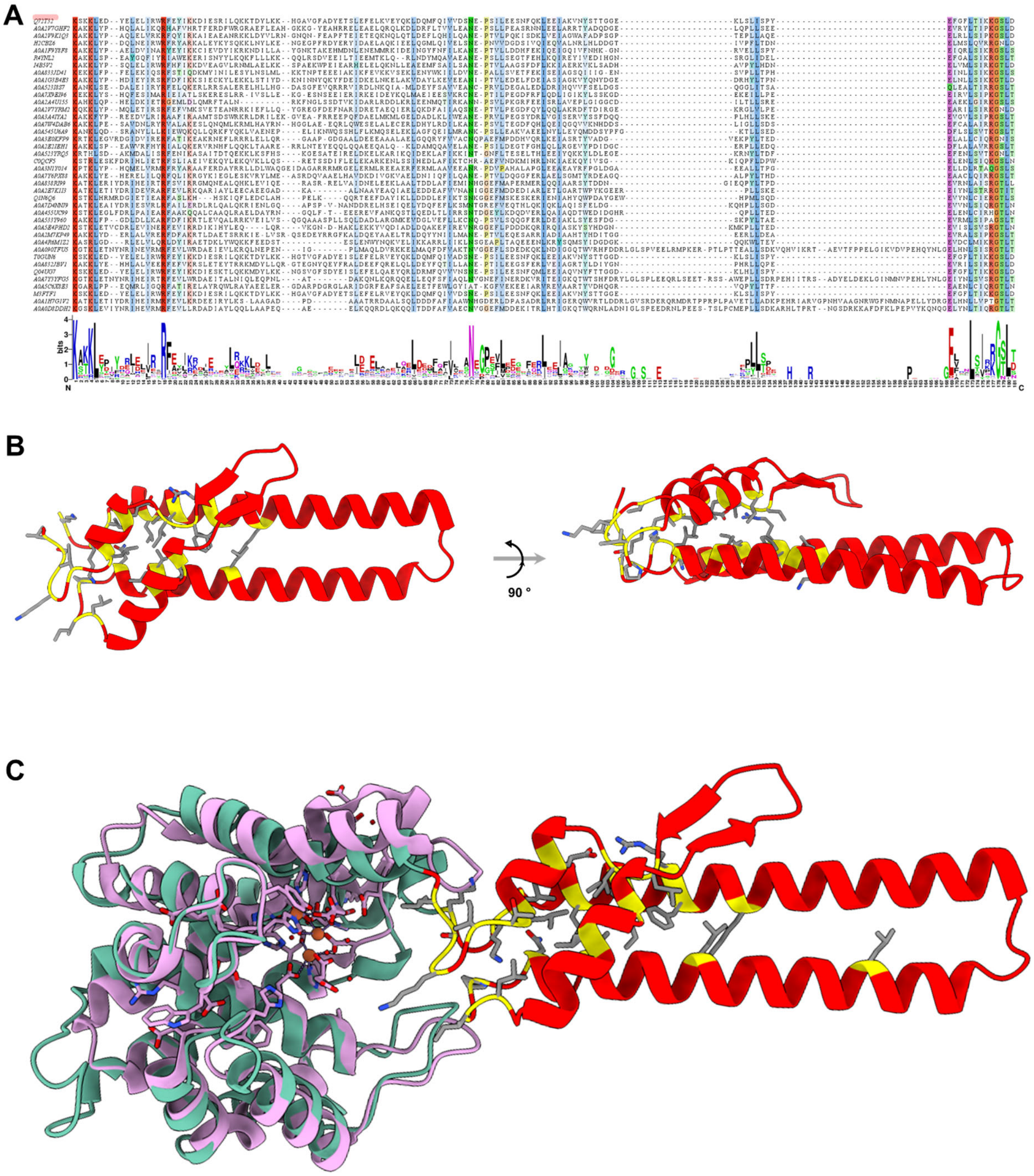

Figure S5. Residue conservation analysis of the HdiD (HD-GYP insertion domain) in LIC_11189.

(A) Sequence alignment of the HD-GYP domain insertion from proteins identified in the FoldSeek AFDB50 search. Highly conserved residues are highlighted using the Clustal color scheme in Jalview [25]. Residue conservation was visualized using WebLogo2 software

[26,27], based on the pre-aligned sequences. **(B)** Cartoon representation of top and side views of the HD-GYP insertion from LIC_11189$_{K416-D536}$, with the insertion colored in red. Conserved residues are highlighted in yellow and shown as sticks, illustrating a hydrophobic pocket that connects the short α-helices and β-sheets to the long α-helices. **C.** Structural alignment of HD-GYP domains from PmGH (light purple) and LIC_11189 (green), displaying the conserved residues in yellow and the atoms as sticks. Cartoon representation of HD-GYP structures colored in ChimeraX version 1.9 [20]

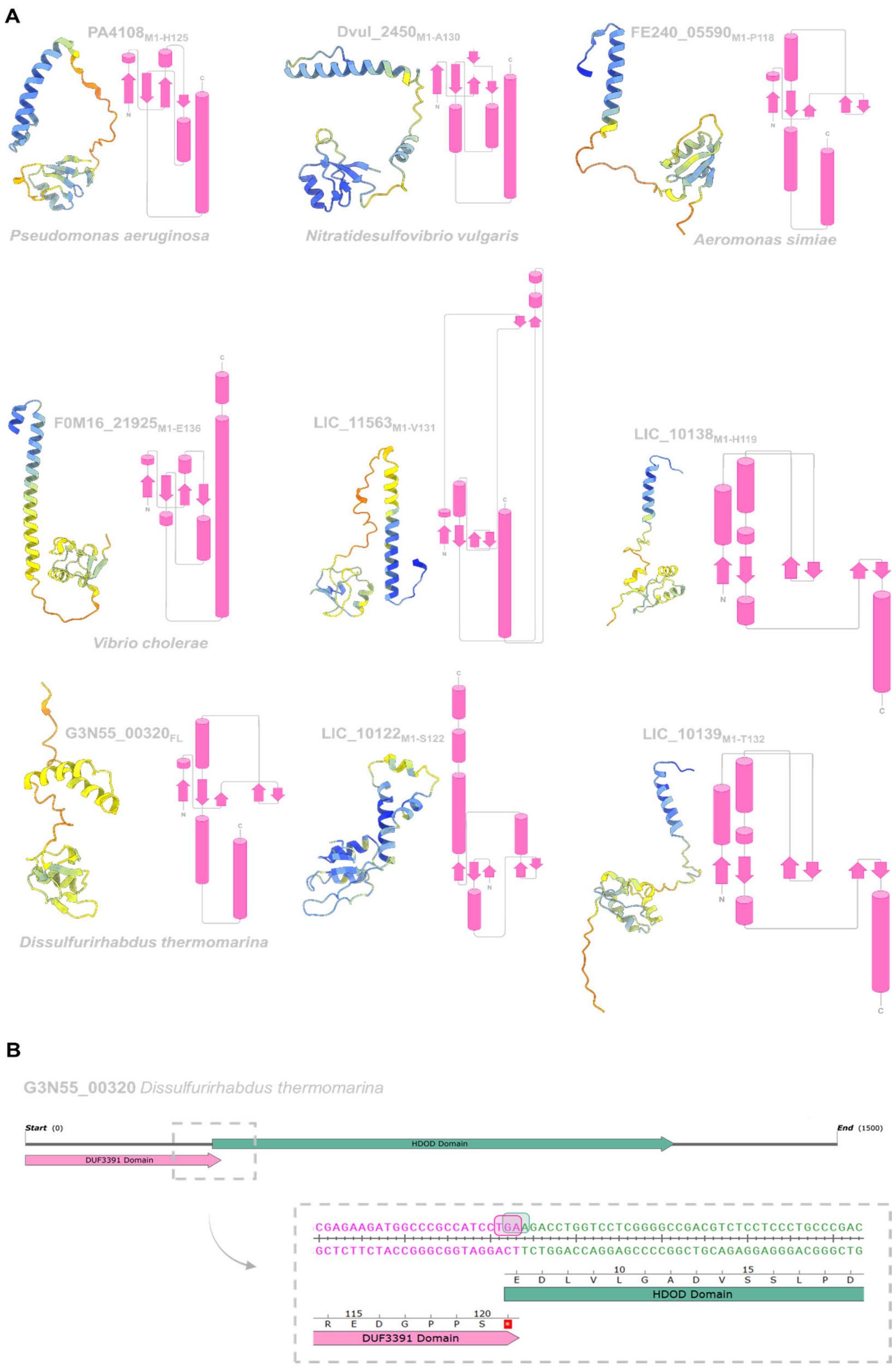

Figure S6. Topology comparison of annotated DUF3391 and N-terminal domains from *L. interrogans* HD-GYP proteins.

(A) Cartoon representations of AlphaFold-predicted structures for DUF3391 domains from *Pseudomonas aeruginosa* (PA4108), *Nitratidesulfovibrio vulgaris* (Dvul_2450), *Aeromonas*

simiae (FE240_05590), *Vibrio cholerae* (F0M16_21925), and *Dissulfurirhabdus thermomarina* (G3N55_00320), alongside N-terminal domains from LIC_10122, LIC_10138, LIC_10139, and LIC_11563. Models are colored based on AlphaFold [22] confidence scores: dark blue (pLDDT > 90), light blue (90 > pLDDT > 70), yellow (70 > pLDDT > 50), and orange (pLDDT < 50). Topology diagrams were generated using PDBsum [6] and manually refined. **(B)** Genomic organization of the DUF3391 protein from *Dissulfurirhabdus thermomarina* (G3N55_00320), showing a mutation that likely resulted in a premature stop codon, separating the DUF3391 domain from the HDOD domain. Cartoon representation of HD-GYP structures colored in ChimeraX version 1.9 [20]

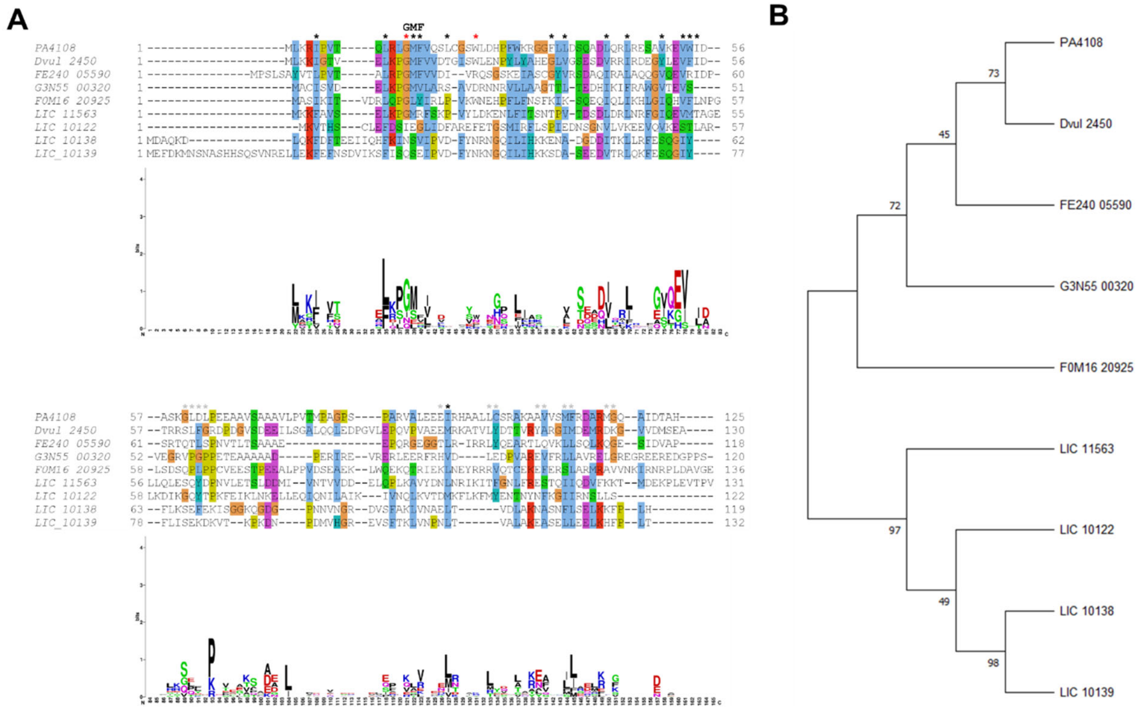

Figure S7. Sequence alignment and phylogeny of the N-terminal domains.

(A) Sequence alignment of DUF3391 domains and the N-terminal domains from *L. interrogans* performed by Clustal and visualized with Jalview [25], with residues colored based on the Clustal Omega [28] scheme. Conserved residues described by Galperin and Chou (2022) [29] are highlighted with asterisks: red asterisks denote conserved Gly12 and Trp22 as identified on the Pfam website [30], while black and gray asterisks mark highly conserved and semi-conserved residues, respectively. Residue conservation analysis for these aligned sequences was generated using WebLogo2 [26,27]. **(B)** Phylogenetic tree of DUF3391 domains and the N-terminal domains from *L. interrogans*, constructed in MEGA software [31] using the Maximum Likelihood (Bootstrap = 100) method based on the aligned sequences .

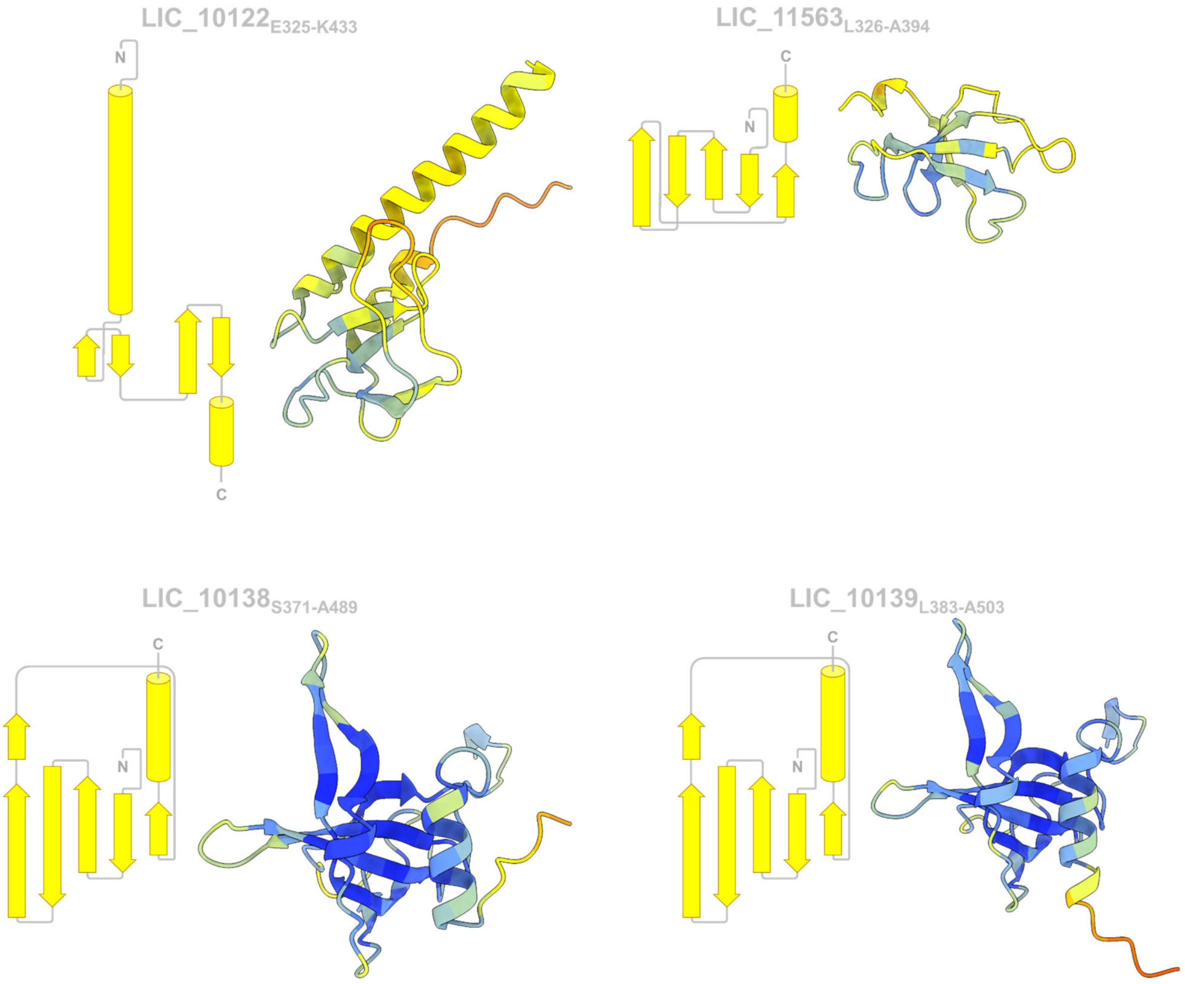

Figure S8. Predicted structure and topology analysis of the C-terminal domains from *L. interrogans* HD-GYP proteins.

Predicted models were generated using AlphaFold 3 [22] and visualized in ChimeraX [20], with confidence scores indicated by color: dark blue (pLDDT > 90), light blue (90 > pLDDT > 70), yellow (70 > pLDDT > 50), and orange (pLDDT < 50). Topology representations were created in PDBsum [6] and manually refined.

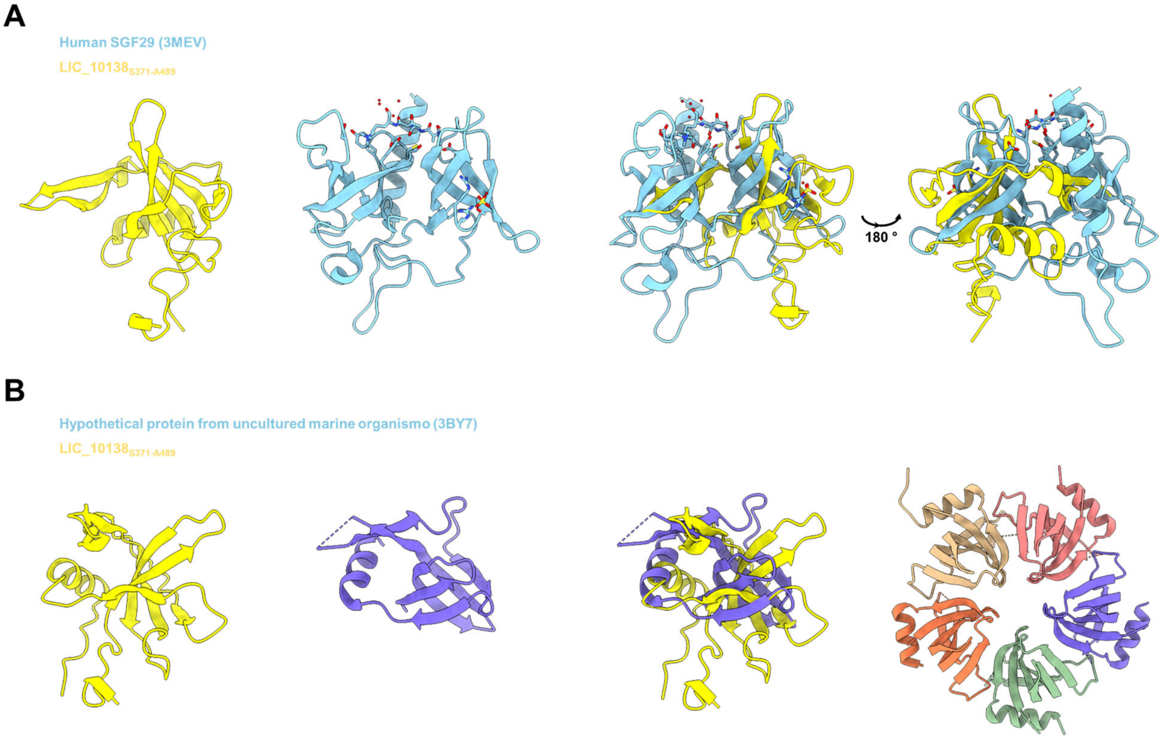

Figure S9. Structural comparison of LIC_10138$_{S371\text{-}A489}$ and its Dali [15] **and FoldSeek** [32] **matches.**

(A) Structural alignment of LIC_10138$_{S371\text{-}A489}$ (yellow) with the human SGF29 (PDB: 3MEV, light blue) [12]. Amino acids from the Tudor domain and the H3K4me3 peptide atoms are shown as sticks. **(B)** Structural alignment of LIC_10138$_{S371\text{-}A489}$ (yellow) with a hypothetical protein from an uncultured marine organism (PDB: 3BY7, purple) [13]. The pentameric form of the latter structure is colored by chains.

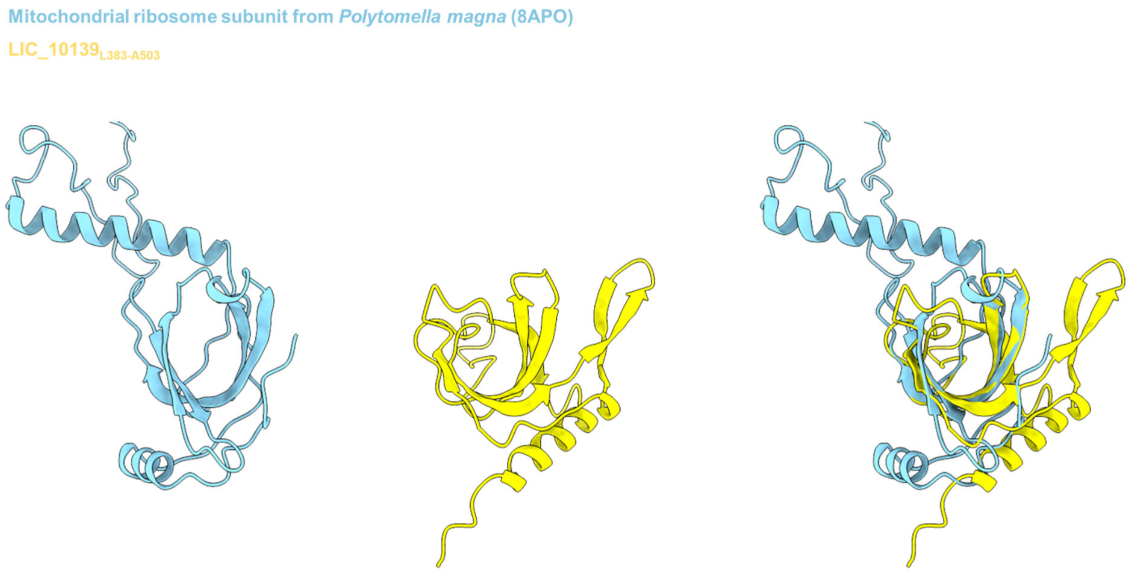

Figure S10. Structural comparison of LIC_101398S371-A489$_{L383\text{-}A503}$ with mitochondrial ribosome subunit and its FoldSeek [32] **best match.**

Structural alignment of LIC_10139$_{L383\text{-}A503}$ (yellow) with the mitochondrial ribosome subunit from *Polytomella magna* (PDB: 8APO [14], light blue).

A

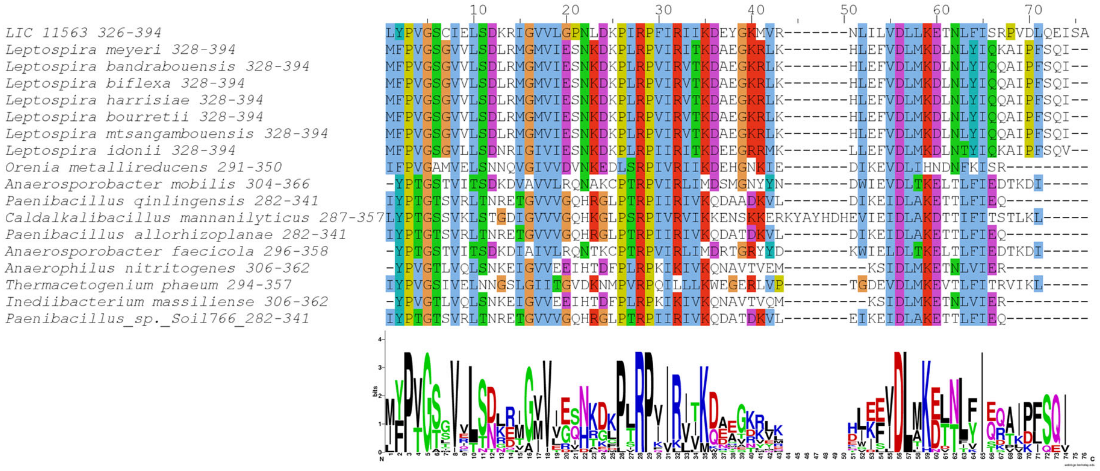

B

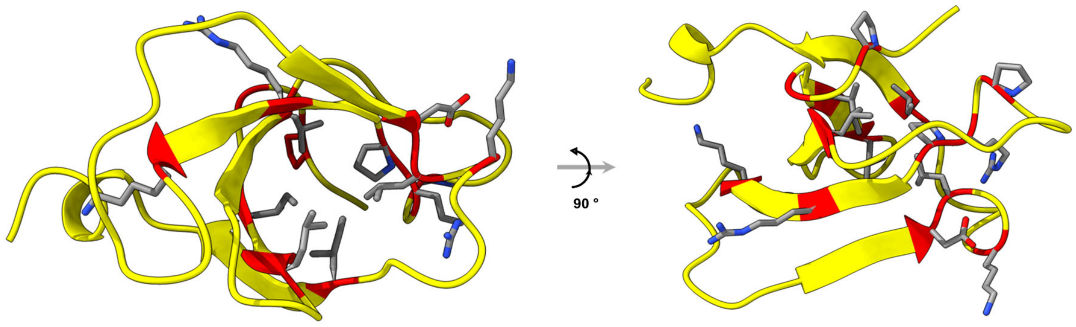

Figure S11. Residue conservation analysis of LIC_11563$_{L326\text{-}A394}$.

(A) Homologous sequences were manually selected from a BLASTp [1] search using LIC_11563$_{L326\text{-}A394}$ as the query, with criteria of query coverage > 85% and sequence identity between 40–55%. Sequences were aligned and colored by Clustal, and residue conservation was visualized using WebLogo 2 [26,27] based on the pre-aligned sequences in Jalview [25]. **(B)** Structural representation of LIC_11563$_{L326\text{-}A394}$ (yellow) highlighting conserved residues (red), generated in ChimeraX [20].

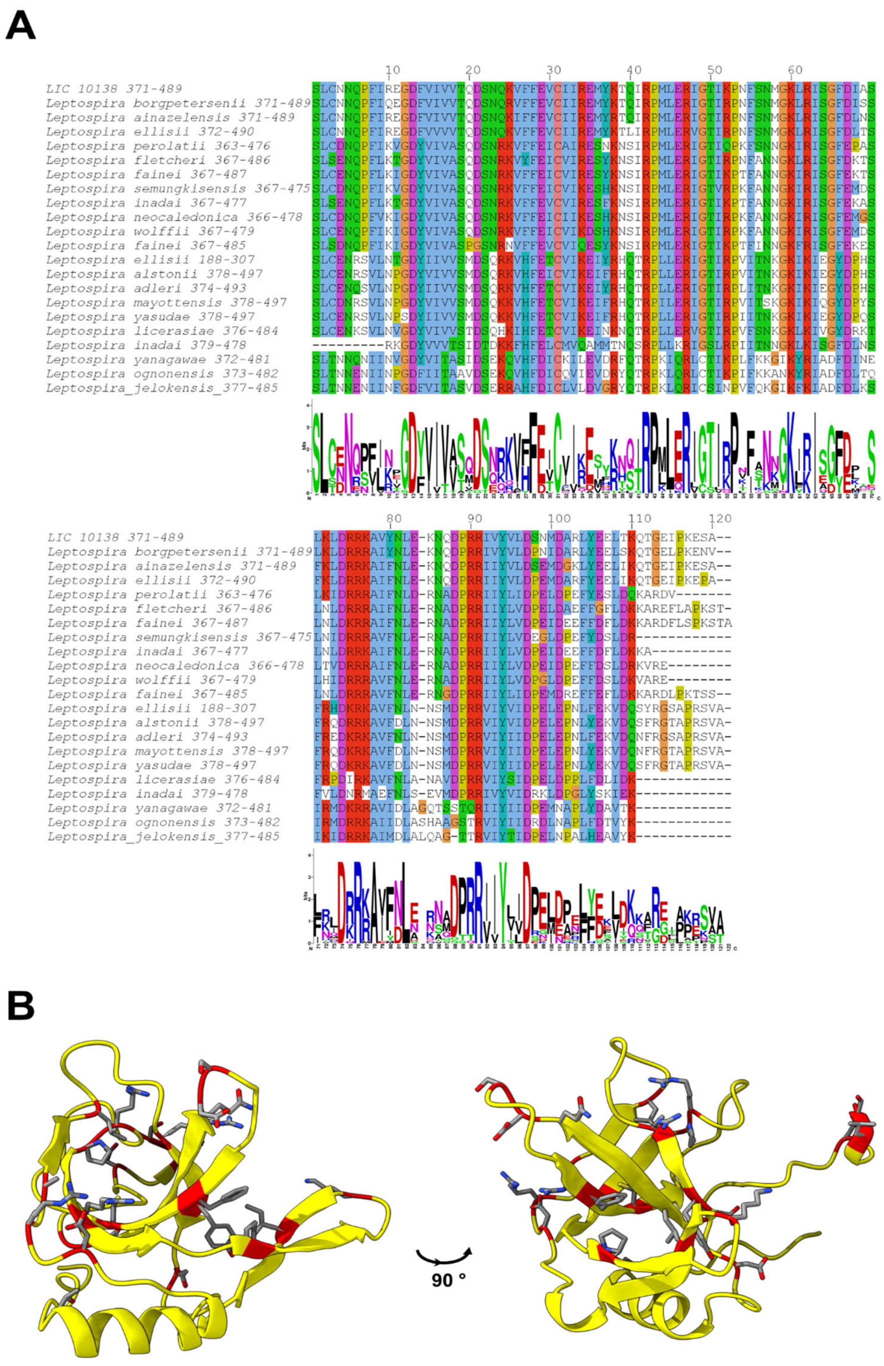

Figure S12. Residue conservation analysis of LIC_10138$_{S371-A489}$.

(A) Homologous sequences were manually selected from a BLASTp [1] search using LIC_10138$_{S371-A489}$ as the query, with criteria of query coverage > 85% and sequence identity between 40–90%. Sequences were aligned and colored by Clustal, redundancy was removed using a value of 95 in Jalview software [25] and residue conservation was visualized using WebLogo 2 [26,27] based on the pre-aligned sequences in Jalview. **(B)** Structural representation of LIC_10138$_{S371-A489}$ (yellow) highlighting conserved residues (red), generated in ChimeraX [20].

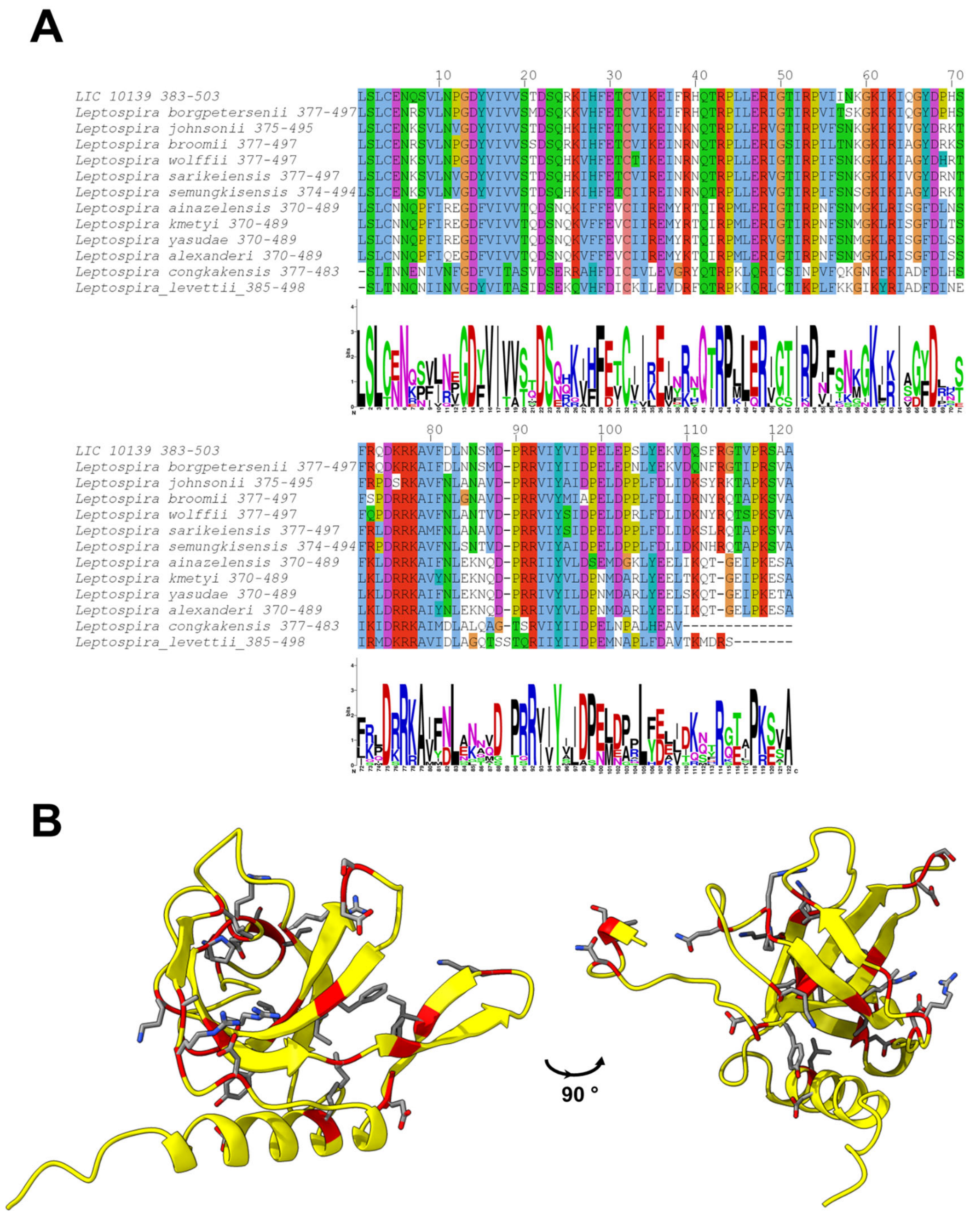

Figure S13. Residue conservation analysis of LIC_10139$_{L383\text{-}A503}$.

(A) Homologous sequences were manually selected from a BLASTp [1] search using LIC_10139$_{L383\text{-}A503}$ as the query, with criteria of query coverage > 85% and sequence identity between 40–90%. Sequences were aligned and colored by Clustal Omega [28], redundancy was removed using a value of 95 in Jalview software [10] and residue conservation was visualized using WebLogo [26,27] based on the pre-aligned sequences in Jalview [25]. **(B)** Structural representation of LIC_10139$_{L383\text{-}A503}$ (yellow) highlighting conserved residues (red), generated in ChimeraX [20].

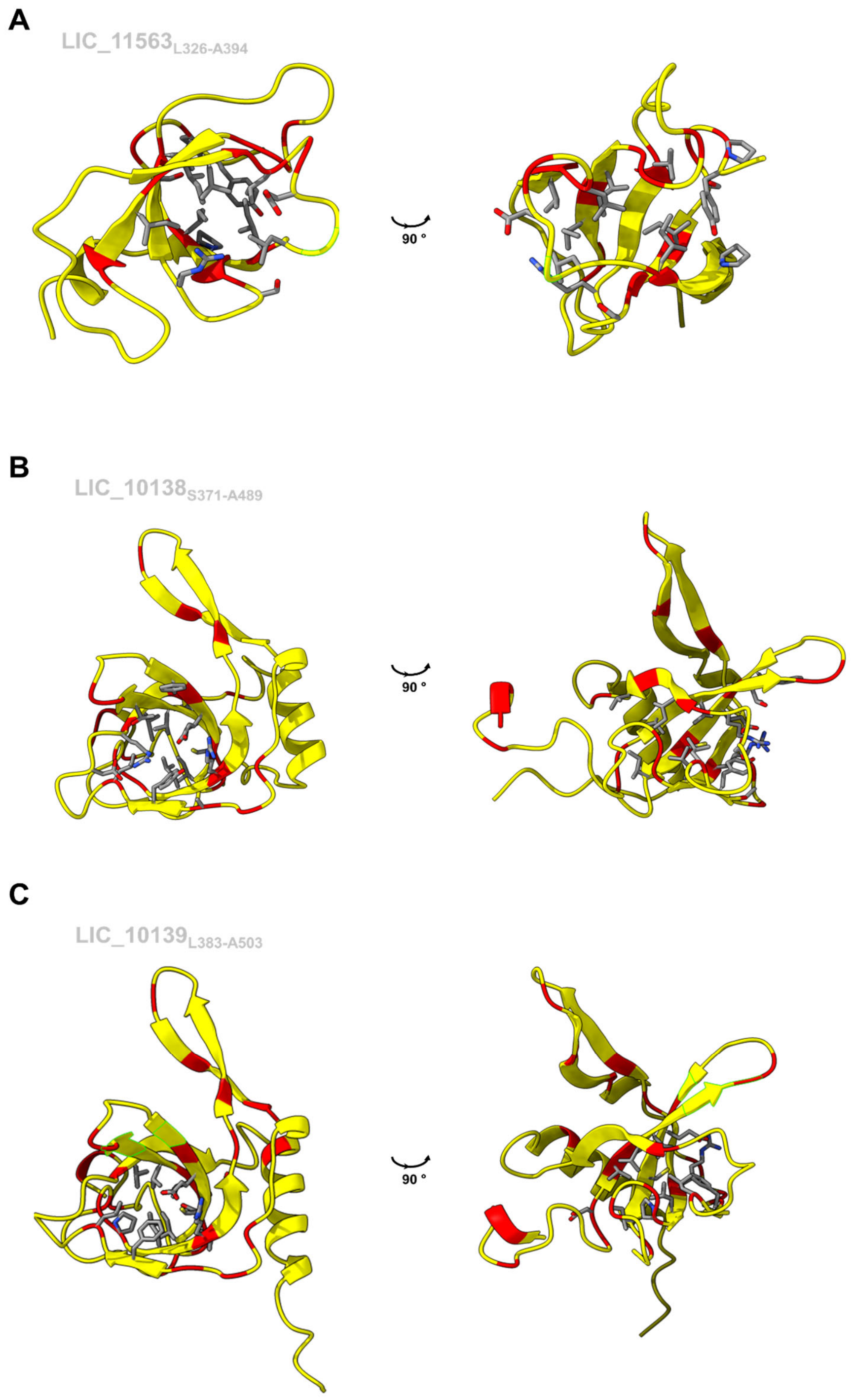

Figure S14. Charges of the side chains of amino acids inward the barrel-like structure of LIC_11563$_{\mathrm{L326\text{-}A394}}$, LIC_10138$_{\mathrm{S371\text{-}A489}}$, and LIC_10139$_{\mathrm{L383\text{-}A503}}$.

(**A**) LIC_11563$_{\mathrm{L326\text{-}A394}}$, (**B**) LIC_10138$_{\mathrm{S371\text{-}A489}}$, and (**C**) LIC_10139$_{\mathrm{L383\text{-}A503}}$. Cartoon structures are shown in yellow, with the conserved residues displayed in red. Side chain residues from amino acids forming the barrel-like structures positioned inward of the pocket are displayed as sticks.

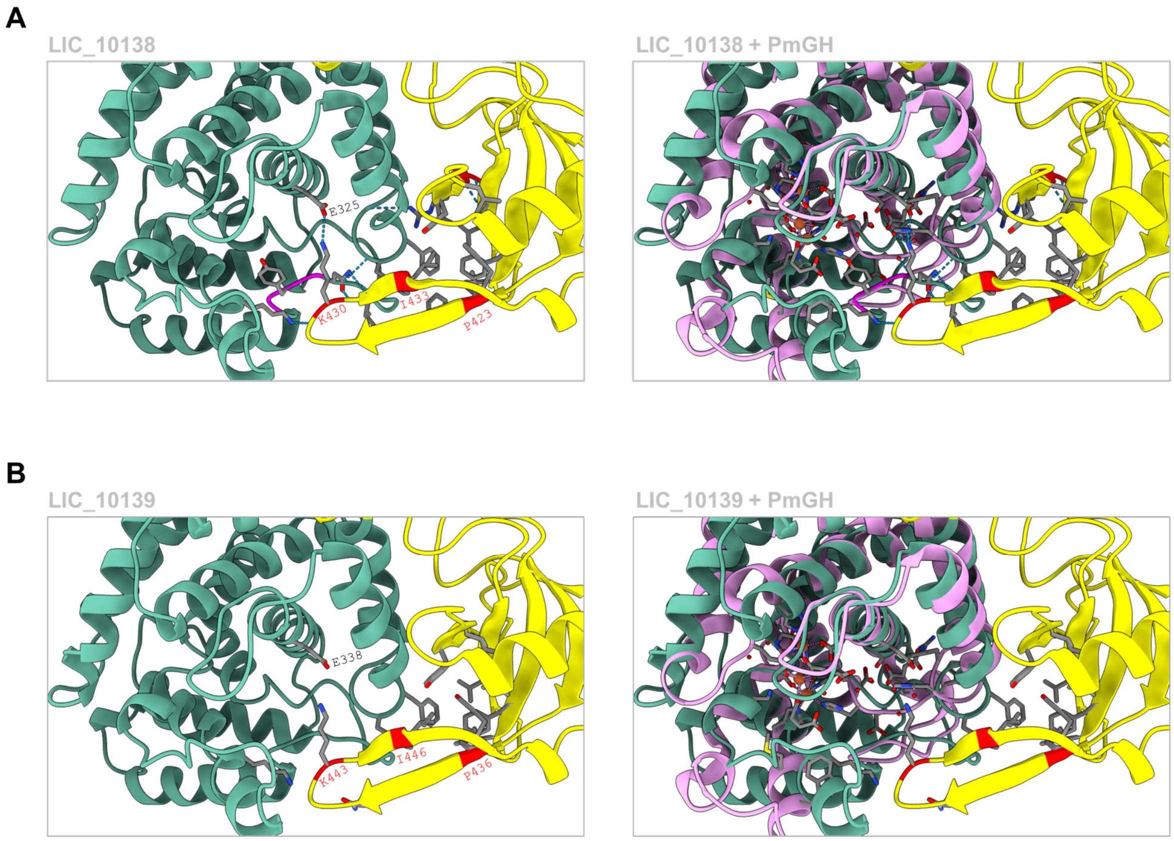

Figure S15. Participation of conserved residues in LIC_10138$_{S371\text{-}A489}$, and LIC_10139$_{L383\text{-}A503}$ in the interaction among the HD-GYP and the C-terminal domain.

Comparison of the domain interaction interface in LIC_10138 (**A**) and (**B**) LIC_10139, including a structural alignment with the HD-GYP domain of the PmGH protein (PDB: 4ME4 [19]). HD-GYP and C-terminal domains in LIC_10138 and LIC_10139 are shown in green and yellow, respectively. Absolutely conserved residues in both proteins are highlighted in red. Amino acid side chains displayed as sticks. The PmGH structure is shown in light purple.

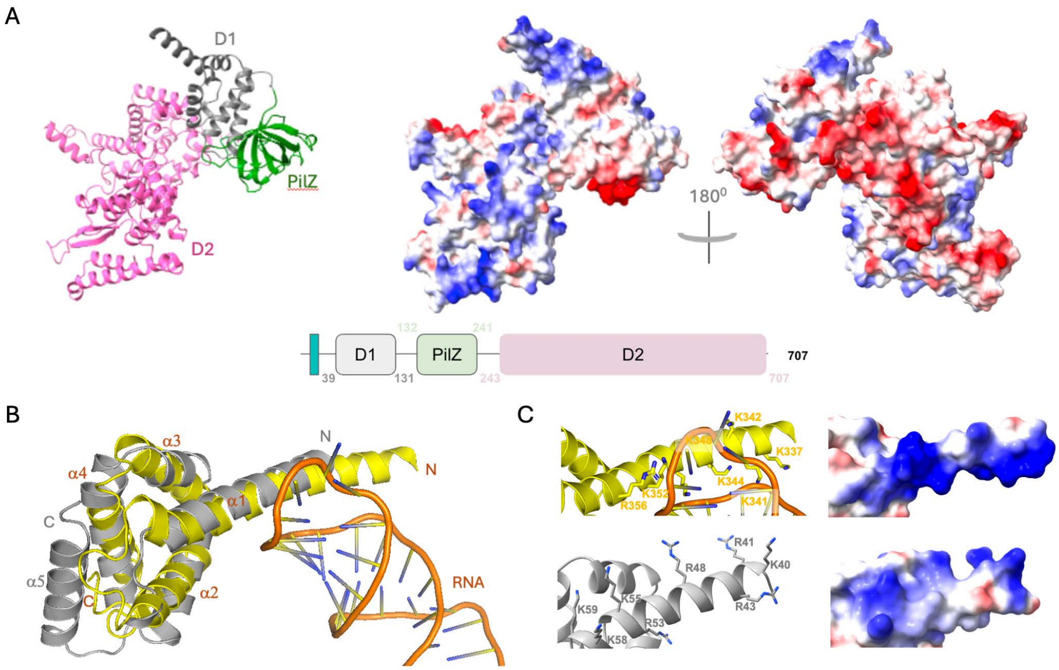
A
D1
PilZ
D2
180⁰
132
241
D1
PilZ
D2
707
39
131
243
707
B
α3
α4
α1
α2
α5
N
N
C
C
RNA
C
K342
K337
K344
K341
K352
R356
R41
K40
R48
R43
K59
K55
R53
K58

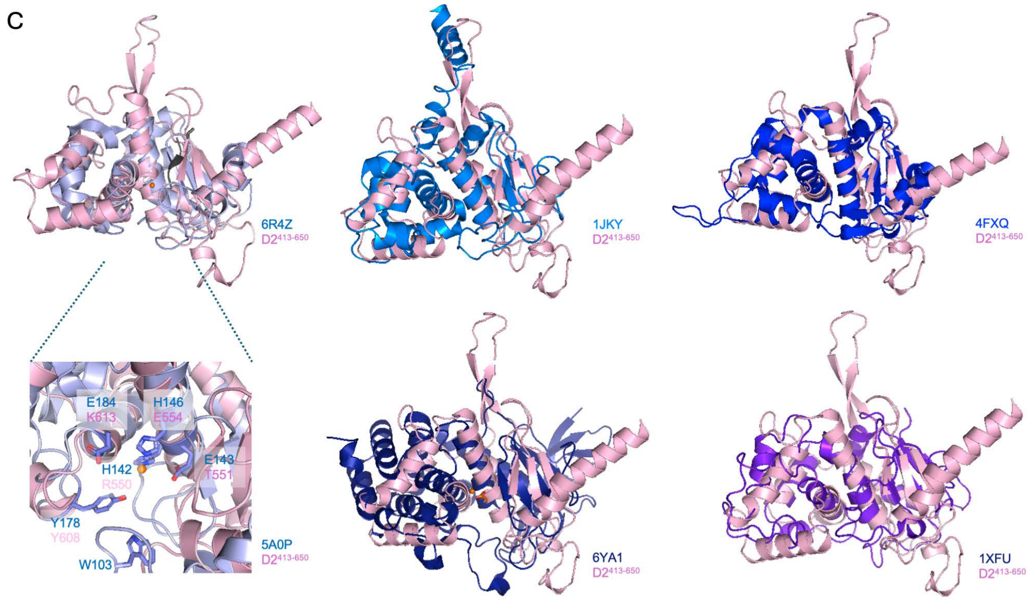
C
6R4Z
D2413-650
1JKY
D2413-650
4FXQ
D2413-650
E184
K613
H146
E554
H142
R550
E143
T551
Y178
Y608
W103
5A0P
D2413-650
6YA1
D2413-650
1XFU
D2413-650

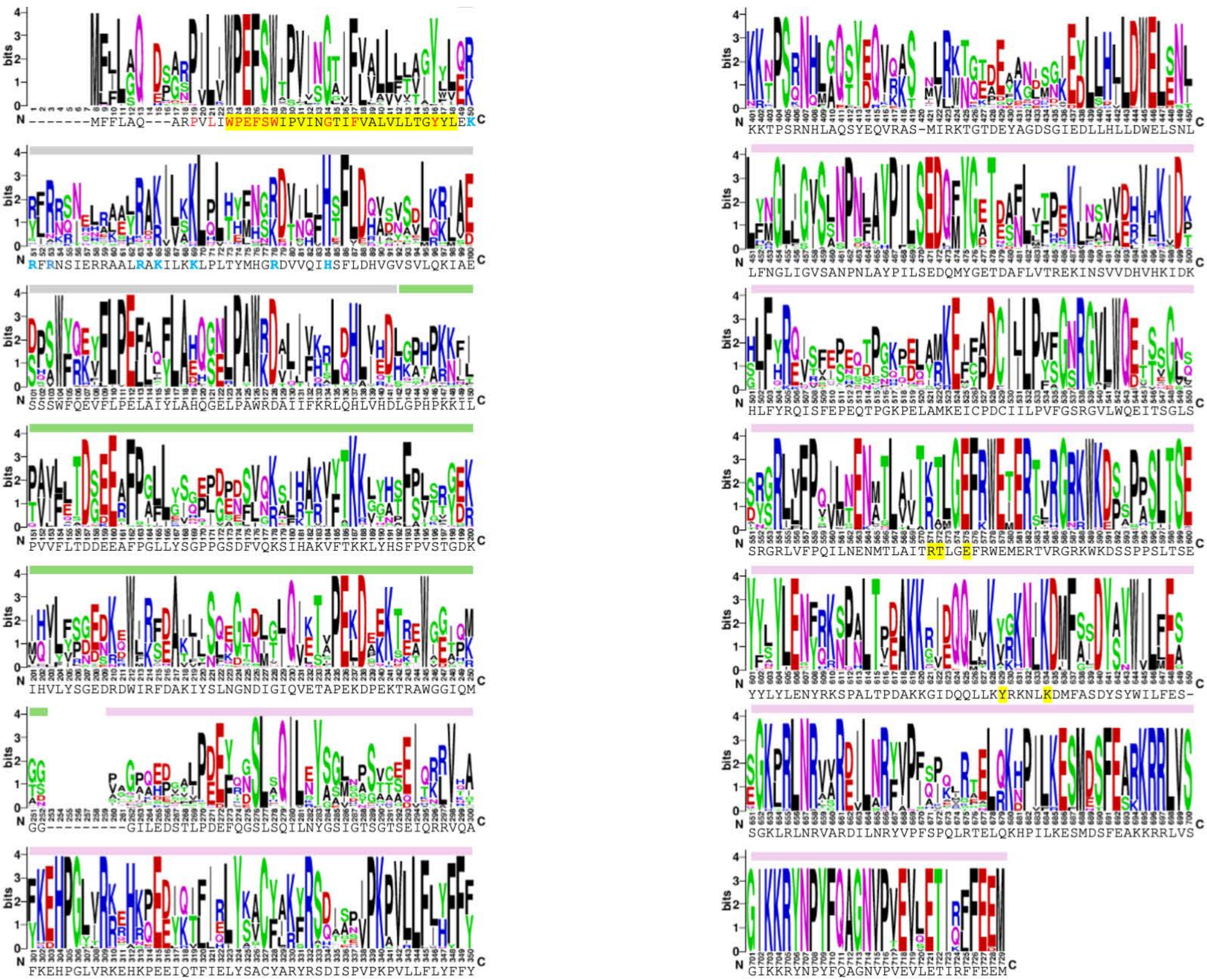
-------MFFLAQ---ARPVLIWPEFSWIPVINGTIFVALVLLTGYYLEK
RFRNSIERRAALRAKILKKLPLTYMHGRDVVQIHSFLDHVGVSVLQKIAE
SSSWFQEVFLPELAIYLAHQGELPAWRDAIIFKRLQHLVHDLGPHPKKIL
PVVFLTDDEEAFPGLLYSGPPGSDFVQKSIHAKVFTKKLYHSFPVSTGDK
IHVLYSGEDRDWIRFDAKIYSLNGNDIGIQVETAPEKDPEKTRAWGGIQM
GG---------GILEDSTLPDEFQGSLSQILNYGSIGTSGTSEIQRRVQA
FKEHPGLVRKEHKPEEIQTFIELYSACYARYRSDISPVPKPVLLFLYFFY
MDENLLSPTRIVQLYETLEKIKD-TQDPYPSDHKLAVYFLPEWLGLILSG
KKTPSRNHLAQSYEQVRAS-MIRKTGTDEYAGDSGIEDLLHLLDWELSNL
LFNGLIGVSANPNLAYPILSEDQMYGETDAFLVTREKINSVVDHVHKIDK
HLFYRQISFEPEQTPGKPELAMKEICPDCIILPVFGSRGVLWQEITSGLS
SRGRLVFPQILNENMTLAITRTLGEFRWEMERTVRGRKWKDSSPPSLTSE
YYLYLENYRKSPALTPDAKKGIDQQLLKYRKNLKDMFASDYSYWILFES-
SGKLRLNRVARDILNRYVPFSPQLRTELQKHPILKESMDSFEAKKRRLVS
GIKKRYNPYFQAGNVPVEVLETIRFFEEM

E

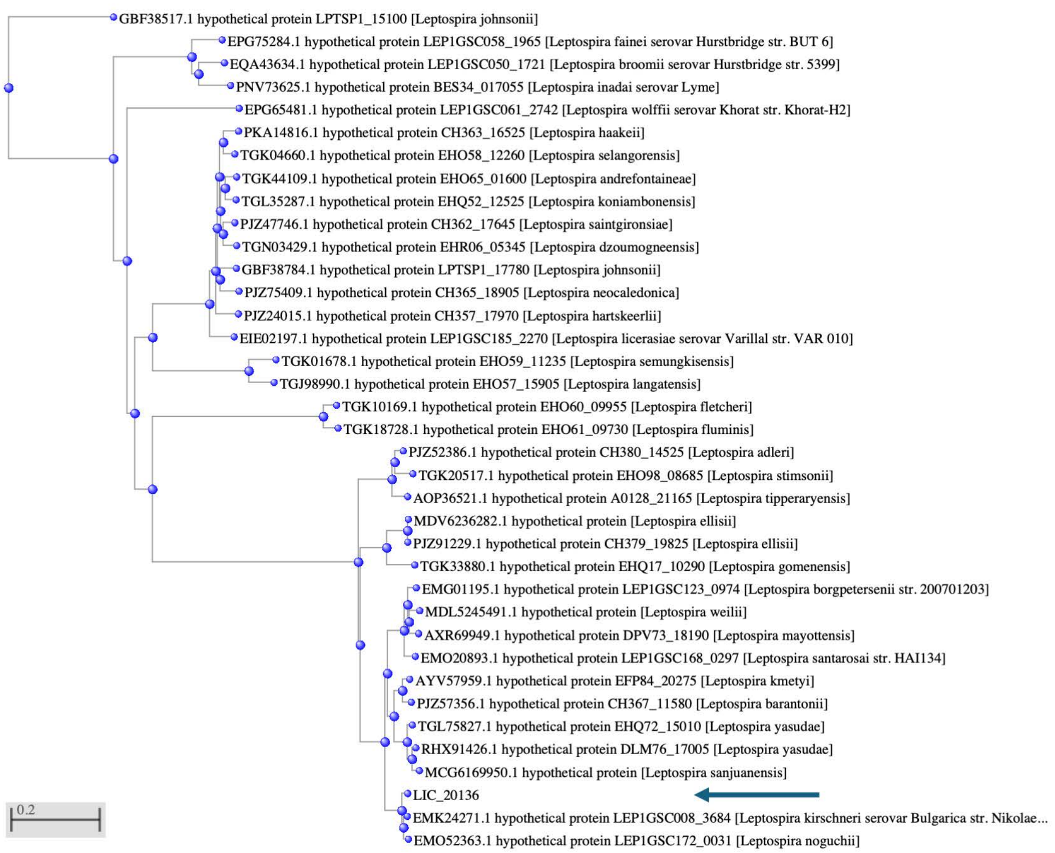

Figure S16. LIC_20136 contains a Transmembrane and D1, PilZ and D2 Domains.

(A) LIC_20136 structure with a transmembrane domain (residues 16-38), a globular pentahelical domain (D1, residues 39-131), a non-canonical PilZ domain (residues 132-241), and a long C-terminal domain (D2, residues 243-707). **(B)** Structural comparison of D1 (in gray) with Zuotin (PDB 7X34 [33], in yellow), which shows structural divergence. **(C)** Structural comparison of D2 (colored in pink) with the ATLF domain of Zmp1 from *Clostridioides difficile* (the peptide recognized by the protein is colored in black, PDB 6R4Z [34]); with the wild type of the ATLF domain of Zmp1 from *Clostridioides difficile* (PDB: 5A0P [35]); with the ATLF domain of the anthrax lethal factor protein (PDB 1JKY [36]); the ATLF domain of the certhrax toxin from *Bacillus cereus* (PDB 4FXQ [37]); the Peptidase_M4 domain of ProA of *Legionella pneumophila* (PDB 6YA1 [38]); with the ATLF-like domain of the Edema factor exotoxin of the *Anthrax* bacteria (PDB 1XFU [39]). **(D)** Conservation amino acid profile represented by a WebLog of orthologs of LIC_20136 (domains of LIC_20136 are represented as bars in the top and the amino acid sequence is shown at the bottom). The residues of the TM domain are highlighted in yellow, in blue are residues positively charged located in the D1 domain, and those highlighted in yellow in the D2 domain are the corresponded residues of the active site of Zmp1 ($H_{142}E_{143}xxH_{146}$ motif and E_{140}, W_{103}, and Y_{178}). **(E)** the phylogenetic tree showing the presence of this protein among *Leptospira* species. The sequences used for multiple sequence alignment were obtained by searching for homologs of the LIC_20136 protein using BLASTp. One representative sequence per species was selected, with identity ranging from 35% to 99% and coverage between 70% and 100%. These sequences were then aligned using COBALT:Multiple Alignment Tool [40], followed by WebLogo analysis [26,27]. The phylogenetic tree was constructed using COBALT.

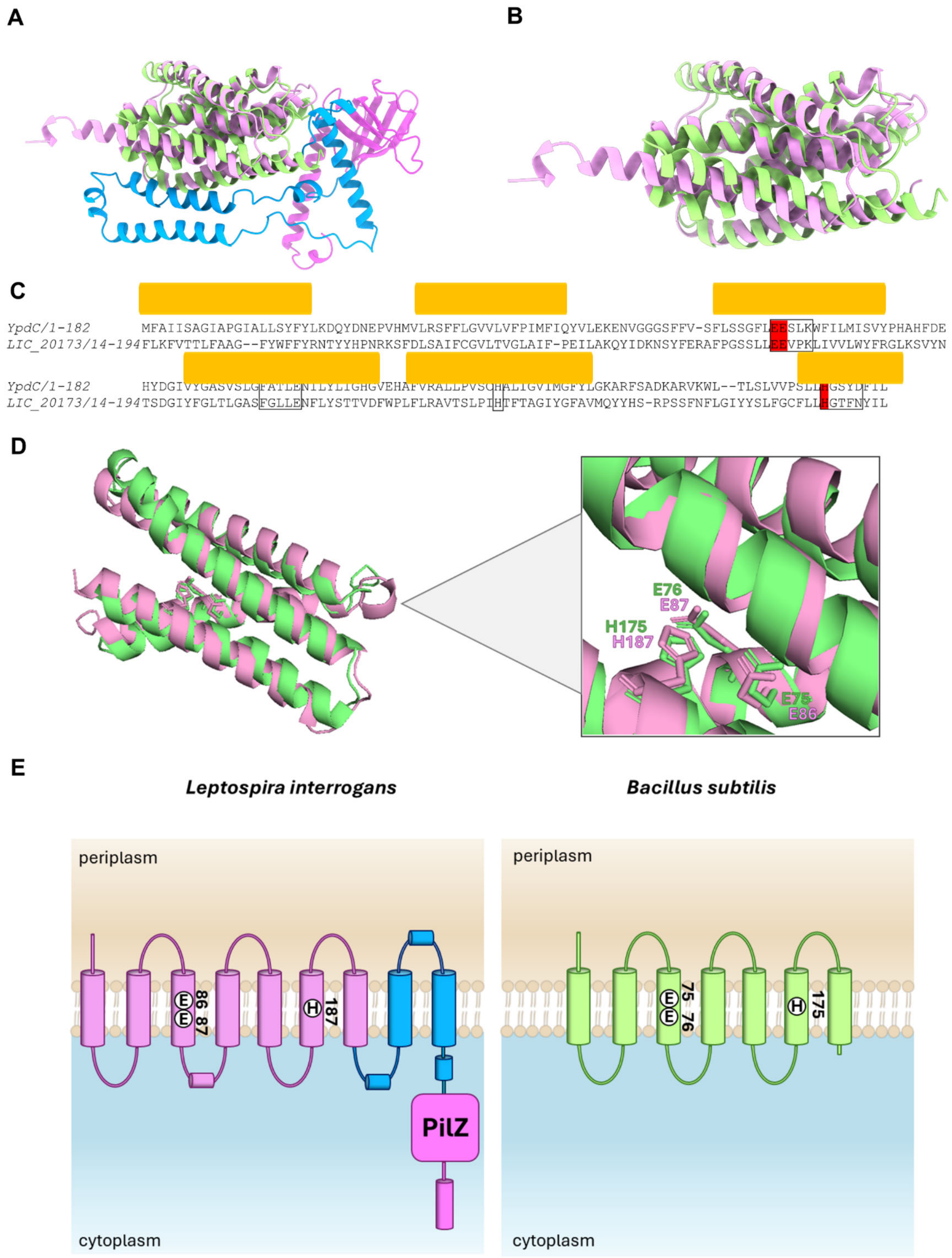

Figure S17. Structure and amino acid sequence alignment of the PrsW domain of the YpdC protein from *B. subtilis* and LIC_20173 from *L. interrogans* serovar Copenhageni strain Fiocruz L1-130.

(A) Structure alignment of the protein PrsW domain of YpdC (in green) and full-length structure of LIC_20173 with the PrsW domain in pink, the four additional helices in blue and the PilZ domain in magenta. **(B)** Structure alignment of the protein PrsW domain of YpdC (in green) and the PrsW domain of LIC_20173 (in pink). **C.** Sequence alignment of the PrsW domain of YpdC and LIC_20173, showing in red the residues necessary for the active protease

activity and in black the four motifs of the PrsW domain: E75E76xxK79, F110xxxE114, H138 and H175xxxD/N179 [41]. The structure of the PrsW domain of YpdC and LIC_20173 has been predicted by Alphafold 3 [22]. The orange bars represents the prediction of the LIC_20173 transmembrane domain by the TMHMM - 2.0 server. **(D)** Comparative alignment of the PrsW domain from the YpdC protein of *B. subtilis* (in green) and the LIC_20173 protein of *L. interrogans* (in pink). The analysis highlights the conservation of key amino acid residues essential for protease activity. In YpdC, these residues are E75, E76, and H175, while in LIC_20173, the corresponding residues are E86, E87, and H187. This strong conservation suggests that LIC_20173 is a functional protease. **(E)** This panel illustrates the insertion of both proteins into the inner membrane of their respective bacteria. The LIC_20173 of *L. interrogans* (left) and the YpdC of *B. subtilis* (right) are shown as transmembrane proteins. This localization supports the hypothesis that LIC_20173 acts as a membrane protease, playing a crucial regulatory role in essential cellular processes [42].The structure of the PrsW domain of YpdC and LIC_20173 has been predicted by Alphafold 3 [22].

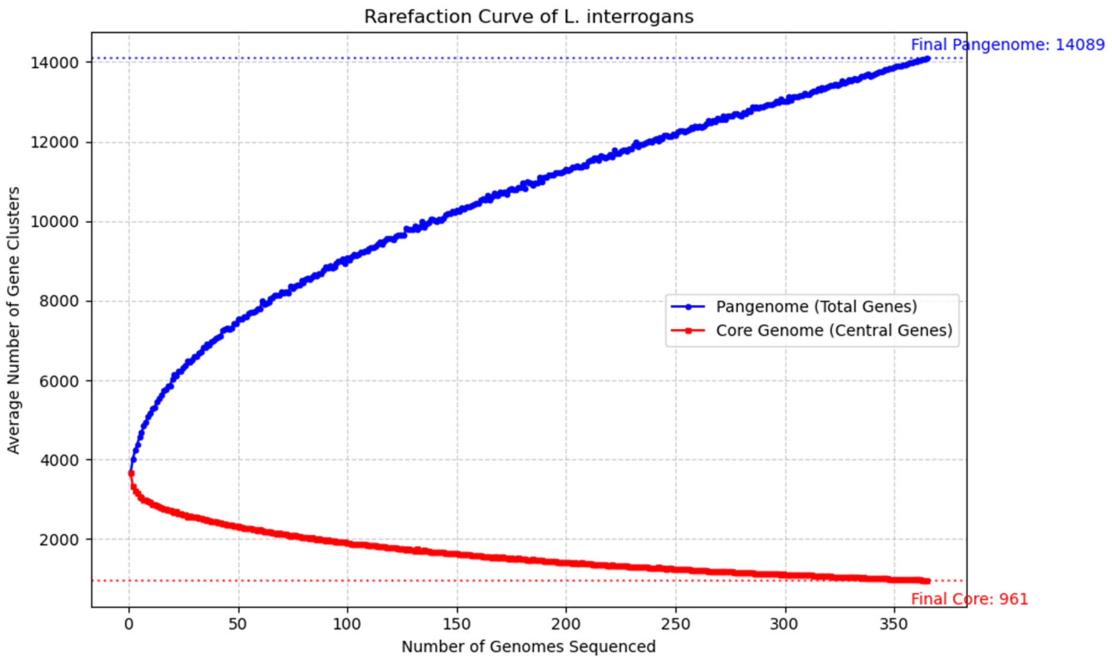

Figure S18. Rarefaction curve of the *L. interrogans* pangenome. The analysis reveals 14,089 genes in the pangenome (blue curve) and 961 core genes (red curve). Based on the slope of the pangenome curve, we can infer that the pangenome is open and novel accessory genes are expected to be discovered as new isolates are sequenced.

[Provided as a separate PDF file in the Supplementary Materials]

Figure S19. Genome-based phylogeny of all 365 *L. interrogans* isolates. The tree was inferred using the neighbor-joining method based on genome similarity estimates calculated with the FastANI software. The heatmap represents the number of genes in each orthologous group related to c-di-GMP turnover in each genome. The header and footer indicate, from top to bottom: (i) the gene identifiers of *L. interrogans* serovar Copenhageni strain Fiocruz L1-130; (ii) the percentage of genomes in the total pangenome that contain members of each group; (iii) the family or subfamily of the c-di-GMP–binding domain; and (iv) the domain architecture of the proteins in each group. The tree was rooted using the midpoint method, and the order of its leaves was used to order the heatmap rows. The figure was rendered using the ete3 library [43].

Table S1 – Keywords to search proteins related to c-di-GMP in the Uniprot database [44]**.**

**GGDEF domains keywords**
GGDEF domain and *Leptospira;* Diguanylate cyclase and *Leptospira;* Cyclic diguanylate phosphodiesterase and *Leptospira;* Bifunctional diguanylate cyclase/phosphodiesterase and *Leptospira*; Sensor domain-containing diguanylate cyclase and *Leptospira*; cGMP-specific phosphodiesterase and *Leptospira*; IPR000160 and *Leptospira*; cd01949 and *Leptospira*; PF00990 and *Leptospira.*
**EAL domain keywords**
Cyclic diguanylate phosphodiesterase and *Leptospira*; EAL domain-containing protein and *Leptospira*; Bifunctional diguanylate cyclase/phosphodiesterase and *Leptospira*; Diguanylate phosphodiesterase and *Leptospira*; Signal transduction protein containing a EAL domain and *Leptospira*; IPR001633 and *Leptospira*; cd01948 and *Leptospira*; PF00563 and *Leptospira.*
**HD-GYP domain keywords**
HD-GYP domain and *Leptospira*; C-di-GMP phosphodiesterase and *Leptospira*; HD 5 and *Leptospira*; IPR037522 and *Leptospira*; cd00077 and *Leptospira.*
**PilZ domain keywords**
PilZ domain and *Leptospira*; Type IV pilus assembly protein PilZ and *Leptospira*; DUF1577 domain-containing protein and *Leptospira*; Pilus assembly protein PilZ and *Leptospira*; PF07614 family protein and *Leptospira*; IPR009875 and *Leptospira*; PF22006 and *Leptospira*; PF07238 and *Leptospira*; PF13487 and *Leptospira.*
**MshEN domain keywords**
MshEN and *Leptospira*; gspE and *Leptospira*; IPR037257 and *Leptospira*; IPR007831 and *Leptospira*; PF05157 and *Leptospira*; PF00437 and *Leptospira*; cd01129 and *Leptospira.*

Table S2 - Table with the proteins identified by Blastp [1] in *L. interrogans* serovar Copenhageni strain Fiocruz L1-130, which may be related to c-di-GMP signaling.

locus_tag	Description
LIC_10030	Protein found in Uniprot database.
LIC_10043	Protein found in Uniprot database.
LIC_10049	Confirmed protein with PilZ domain and found in Uniprot and NCBI c-di-GMP database.
LIC_10122	Confirmed protein with HD-GYP in our analysis. In the UniProt database, it is annotated as an "uncharacterized protein", with no previous association with c-di-GMP signaling.domain and found in Uniprot database.
LIC_10128	Confirmed protein with PilZ domain and found in Uniprot and NCBI c-di-GMP database.
LIC_10138	Confirmed protein with HD-GYP domain and found in Uniprot, NCBI c-di-GMP database, and in literature.
LIC_10139	Confirmed protein with HD-GYP domain and found in Uniprot, NCBI c-di-GMP database, and in literature.
LIC_10321	Confirmed protein with GGDEF domain in our analysis. In the UniProt database, it is annotated as an "uncharacterized protein," with no previous association to c-di-GMP signaling.
LIC_10384	Protein found in Uniprot database.
LIC_10481	Protein found in Uniprot database.
LIC_10486	Protein found in Uniprot database.
LIC_10641	Confirmed protein with EAL domain and found in Uniprot database and in literature.
LIC_10986	Protein found in Uniprot database.
LIC_10996	Confirmed protein with EAL domain and found in Uniprot, NCBI c-di-GMP database, and in literature.
LIC_11079	Protein found in Uniprot database.
LIC_11125	Confirmed protein with GGDEF domain and found in Uniprot, NCBI c-di-GMP database, and in literature.

LIC_11126	Confirmed protein with GGDEF domain and found in Uniprot, NCBI c-di-GMP database, and in literature.
LIC_11127	Confirmed protein with GGDEF domain and found in Uniprot, NCBI c-di-GMP database, and in literature.
LIC_11128	Confirmed protein with GGDEF domain and found in Uniprot, NCBI c-di-GMP database, and in literature.
LIC_11129	Confirmed protein with GGDEF domain and found in Uniprot, NCBI c-di-GMP database, and in literature.
LIC_11130	Confirmed protein with GGDEF domain and found in Uniprot, NCBI c-di-GMP database, and in literature.
LIC_11131	Confirmed protein with GGDEF domain and found in Uniprot, NCBI c-di-GMP database, and in literature.
LIC_11155	Protein found in Uniprot database.
LIC_11167	Confirmed protein with GGDEF domain in our analysis. In the UniProt database, it is annotated as an "putative lipoprotein", with no previous association with c-di-GMP signaling.and found in the Uniprot database.
LIC_11189	Confirmed protein with HD-GYP domain and found in Uniprot, NCBI c-di-GMP database, and in literature.
LIC_11198	Protein found in Uniprot database.
LIC_11203	Confirmed protein with EAL domain and found in Uniprot, NCBI c-di-GMP database, and in literature.
LIC_11247	Protein with a GGDEF domain confirmed in our analysis. In the UniProt database, it is annotated as a "cGMP-specific phosphodiesterase", linked to c-di-GMP signaling but classified as a phosphodiesterase rather than a diguanylate cyclase. Confirmed protein with GGDEF domain and found in the Uniprot database.
LIC_11300	Confirmed protein with GGDEF domain and found in Uniprot, NCBI c-di-GMP database, and in literature.
LIC_11433	Protein found in Uniprot database.
LIC_11437	Protein found in Uniprot database.
LIC_11444	Confirmed protein with GGDEF domain and found in Uniprot, NCBI c-di-GMP database, and in literature.

LIC_11447	Confirmed protein with PilZ domain in our analysis. In the UniProt database, it is annotated as an “uncharacterized protein”, with no previous association with c-di-GMP signaling.and found in Uniprot database.
LIC_11563	Confirmed protein with HD-GYP domain and found in Uniprot, NCBI c-di-GMP database, and in literature.
LIC_11571	Confirmed protein with MshEN domain and found in Uniprot and NCBI c-di-GMP database.
LIC_11628	Protein found in Uniprot database.
LIC_11706	Confirmed protein with GGDEF domain in our analysis. In the UniProt database, it is annotated as an “uncharacterized protein”, with no previous association with c-di-GMP signaling.and found in the Uniprot database.
LIC_11838	Protein found in Uniprot database.
LIC_11914	Protein found in Uniprot database.
LIC_11920	Confirmed protein with PilZ domain and found in Uniprot and NCBI c-di-GMP database.
LIC_11921	Confirmed protein with EAL domain and found in Uniprot, NCBI c-di-GMP database, and in literature.
LIC_11993	Confirmed protein with PilZ domain and found in Uniprot database.
LIC_12087	Protein found in Uniprot database.
LIC_12273	Confirmed protein with GGDEF domain and found in Uniprot, NCBI c-di-GMP database, and in literature.
LIC_12491	Confirmed protein with PilZ domain and found in Uniprot and NCBI c-di-GMP database.
LIC_12505	Confirmed protein with GGDEF and EAL domain and found in Uniprot, NCBI c-di-GMP database, and in literature.
LIC_12546	Confirmed protein with PilZ domain and found in Uniprot and NCBI c-di-GMP database.
LIC_12616	Protein found in Uniprot database.
LIC_12628	Protein found in the Uniprot database and annotated like a PilZ domain.

LIC_12641	Protein found in Uniprot database.
LIC_12723	Confirmed protein with PilZ domain and found in Uniprot and NCBI c-di-GMP database.
LIC_12979	Protein found in Uniprot database.
LIC_12994	Confirmed protein with PilZ domain and found in Uniprot and NCBI c-di-GMP database.
LIC_12997	Protein found in Uniprot database.
LIC_13030	Protein found in Uniprot database.
LIC_13082	Protein found in Uniprot database.
LIC_13120	Confirmed protein with GGDEF and EAL domain and found in Uniprot, NCBI c-di-GMP database, and in literature.
LIC_13127	Protein found in Uniprot database.
LIC_13137	Confirmed protein with GGDEF domain and found in Uniprot, NCBI c-di-GMP database, and in literature.
LIC_13146	Protein found in Uniprot database.
LIC_14002	Confirmed protein with PilZ domain and found in Uniprot and NCBI c-di-GMP database.
LIC_20100	Protein found in Uniprot database.
LIC_20106	Confirmed protein with GGDEF and EAL domain and found in Uniprot, NCBI c-di-GMP database, and in literature. But the gene was described as having only EAL domain.
LIC_20136	Confirmed protein with PilZ domain in our analysis. In the UniProt database, it is annotated as an “uncharacterized protein”, with no previous association with c-di-GMP signaling.and found.
LIC_20173	Confirmed protein with PilZ domain and found in Uniprot and NCBI c-di-GMP database.
LIC_20180	Confirmed protein with GGDEF and EAL domain and found in Uniprot, NCBI c-di-GMP database, and in literature.

LIC_20181	Confirmed protein with GGDEF domain and found in Uniprot, NCBI c-di-GMP database, and in literature.
LIC_20182	Confirmed protein with GGDEF domain and found in Uniprot, NCBI c-di-GMP database, and in literature.
LIC_20198	Confirmed protein with GGDEF and EAL domain and found in Uniprot, NCBI c-di-GMP database, and in literature. But the gene was described as having only EAL domain.
LIC_20201	Protein found in Uniprot database.
LIC_20271	Protein found in Uniprot database.
LIC_20272	Protein found in Uniprot database.

Table S3 - Sensor and signaling domains found in proteins associated with c-di-GMP signaling in the *L. interrogans* genome.

Short Name of Sensor Domain (Pfam [30] and InterPro [23] IDs)	Possible function	Ref.
dCACHE_1<br><br>(PF02743 and IPR033479)	CACHE is an acronym for calcium channels and chemotaxis receptors, and dCACHE means double CACHE domain 1. The dCACHE_1 domain is predicted to be located in the periplasm of Gram-negative bacteria due to the presence of a transmembrane (TM) α-helices, with three strands between them that form a β-sheet that is similar to the PAS domain fold. The dCACHE_1 domain is predicted to bind different small-molecules in a wide range of proteins. Some examples of dCACHE_1 domain ligands are: amino acids, citrate, and autoinducer 2 (AI-2), but may also recognize molecules not yet described.	[45–48]
GAF<br><br>(PF13185 and IPR003018)	GAF is an acronym for cGMP-phosphodiesterase, *Anabaena* adenylyl cyclases, and *E. coli* FhlA. The GAF domain is a cytoplasmic sensory domain that shares a similar fold to the PAS domain. The structure contains a six strands antiparallel β-sheet with 2 to 4 stabilizing α-helices. The GAF domain, as observed for the dCACHE_1 domain, is predicted to bind different small-molecules in a wide range of proteins that may perform different biological functions. Some examples of GAF ligands are: cAMP, cGMP, phycocyanobilin, biliverdin IXα, and others.	[48–50]
PAS<br><br>(PF13188 and IPR000014)	PAS is an acronym for Per-Arnt-SIM. PAS domains are cytoplasmic sensory and the structure is characterized by the PAS fold, which is composed of five strand antiparallel β-sheets and flanked by several α-helices. Although the conserved three-dimensional structure of the PAS fold, the sequence similarities may be as low as 20%, making it highly diverse. Moreover, the PAS domain is also find in a wide range of proteins and binds different small molecules to regulate the protein function, some examples of ligands are: p-coumaric acid, flavin adenine dinucleotide (FAD), flavin mononucleotide (FMN), heme, metals, fatty acids, autoinducers, and others.	[45,48, 51–54]
REC<br><br>*Response_reg*<br><br>(PF00072 and IPR001789)	REC domain is an acronym for phosphoacceptor receiver domain of RRs (response regulators) and PRRs (pseudo response regulators), and is posttranslationally modified by a partner kinase making part of two-component systems. The REC domain may be found in a wide range of proteins modulating by the two-component system a broad range of bacterial function, such as membrane transport, chemotaxis, sporulation, pathogenicity, and virulence. Structurally, the REC domain has a 5 β-strands parallel that is situated in between α-helices (2 on one side and 3 on the other). The 3 β-strands contain 6 residues that are essential for phosphotransfer from the kinase to the target aspartate residue, which is phosphorylated by the kinase. From the phosphorylation pocket, the phosphoryl group forms several hydrogen bonds and salt bridges that make positional changes in the β-strands of the REC domain, changing the activity of the adjacent output domain.	[48,55–57]

YkuI_C<br><br>(PF10388 and IPR018842)	In most cases, the YkuI_C domain is located immediately downstream of the EAL domain (IPR001633). Therefore, in general, YkuI_C is located in the C-terminal region of proteins containing EAL domains. Together, these domains form a monomer that may dimerizes to carry out the enzymatic function of the EAL domain. YkuI consists of three α-helices and five β-strands.	[48,58]
DUF3391<br><br>(PF11871 and IPR021812)	Domain of unknown function 3391 (DUF3391) is located in the N-terminal portion of a wide number of bacterial proteins and it is often found associated with the HD_5 domain. The structure is formed by four β-strands with a 3_{10} helix between strands 3 and 4, followed by two long α-helices.	[29]
GAZ<br><br>DUF1577<br><br>(PF07614 and IPR011471)	The GAZ domains refer to *GAF Associated* with *PilZ*. The GAZ domain is a divergent GAF that was inserted within a PilZ domain. The GAZ domain doesn't have essential structural elements for dimerization and distinguishes GAZ domains from conventional GAF domains that often dimerizes. Most of the GAZ domain was observed in a novel family of YcgR proteins, $YcgR^{GAZ}$, among Spirochaetes.	[59]
PrsW-protease<br><br>(PF13367 and IPR026898)	PrsW-protease domain is a peptidase domain that one member from *Bacillus subtilis* (protein annotated as YpdC) was experimentally characterized and cleavages the anti-sigma factor RsiW in the site 1. PrsW-protease binds antimicrobial peptides by a cluster of acidic residues and senses cell envelope stress. This domain contains four predicted core transmembrane segments and exhibits similar sets of sequence motifs: 'EExxK', 'FxxxE', a conserved histidine, and 'HxxxB'. The PrsW-protease domain may be found in a wide broad protein with different domain architecture, suggesting a participation of this domain in different biological processes.	[42,60]
T2SSE<br><br>(IPR001482<br><br>and PF00437)	The T2SSE domain belongs to the P-loop_NTPase Clan that has an ATPase activity. This domain is found in ATPases belonging to the Type II protein secretion system (T2SS), the Type 4 pilus (T4P), and the Type IV protein secretion system (T4SS) found in Gram-negative bacteria. This domain has four conserved sequence motifs to bind and hydrolyze ATP: the Walker A box with the P loop $GX_4GK(S/T)$, the atypical Walker B box motif Dh_4GE (h stands for hydrophobic residue), the His box, and the Asp box. Walker A binds ATP and Walker B performs the hydrolysis.	[61–63]

Table S4 - Functions of the key residues in the HD-GYP domain, using as a model of the protein of *Persephonella marina* (locus_tag PERMA_0986) [19]**.**

**Domain and protein reference**	**Residue**	**Function**	**Other informations**	**References**
HD-GYP *P.marina* PmGH (PERMA_0986)	E/D185	Metal binding (binds to Me2 and Me3). Residue important for PDE-B activity.	Proteins that have D or E residue hydrolyze c-di-GMP to two GMP. In some proteins, like in *Pseudomonas aeruginosa* (PA4781) has a proline and in *Vibrio cholerae* (VCA0681) has a phenylalanine, have a PDE-B activity. Proteins that have a tyrosine, like in *Aquifex aeolicus* (Aq_2027), don't have a PDE-B activity.	[19,29]
	H189	Metal binding (binds to Me3).		[19,29]
	H221 and D222	Metal binding.	H221 binds to metal 3 and D222 binds to metal 1 and 2. Alanine replacements of the residues H221 and D222 caused reduction or abolished activity of PDE in *P. maritima.* However in *B. burgdorferi* (BB_0374), D222K was replaced and still has activity.	[19,29,64,65]
	K225	Metal binding (binds to Me2).	Cooperates in stabilizing the tri-iron centre and responsible for the protonation of the leaving group during the nucleophilic attack. K225A mutation abolished enzymatic activity in *P. maritima*. However, in BB_0374, K225F still has activity.	[19,29,64,65]
	K235	Chain carbonyl oxygen binds N2 of c-di-GMP Gua2.		[19,29,65,66]
	G237	Chain carbonyl oxygen binds N1 of c-di-GMP Gua2.		[19,29,65,66]

	H250	Metal binding (binds to Me1).	H250A mutation abolished enzymatic activity. However, in BB_0374, H250Y still has activity.	[19,29,65]
	H276	Metal binding (binds to Me1).	H276A mutation abolished enzymatic activity in *P. maritima.*	[19,29]
	H277	Metal binding (binds to Me1).	H277A mutation abolished enzymatic activity in *P. maritima*. In BB_0374, H277 was replaced by K and still has activity.	[19,29,65]
	E278	Site of binding to c-di-GMP and structuralstrucutural stabilization.	Stabilizes the 18-aa loop between α4 and α5.	[29]
	G284		Conserved residue in the GYP motif, Stabilizes the 18-aa loop between α4 and α5. Catalytic activity was not significantly altered with the G284A mutation, PPI interaction was decreased.	[19,29,67]
	Y285		Binds nonbridging O of c-di-GMP ribose-phosphate ring. Catalytic activity was not significantly altered with the Y285A mutation, PPI interaction was decreased.	[19,29,67]
	P286		Conserved residue in the GYP motif, Stabilizes the 18-aa loop between α4 and α5. Catalytic activity was not significantly altered with the P286A mutation, PPI interaction was decreased.	[19,29,67]
	I294	Structural stabilization.	Catalytic activity was not significantly altered with the I294A substitution	[19,29]
	D305	Metal binding (binds to Me3).	D305A substitution causes decreased enzymatic activity. In BB_0374*,* D305S was replaced and still has activity.	[19,29,65]

	D308	Conserved residue.	The D308A mutation abolished enzymatic activity. In some proteins, like BB_0374, D308S or in VCA0681, D308Q still has activity.	[19,29,65]
	A309	Hydrophobic interaction with c-di-GMP guanine base Gua1.		[29]
	L310	Hydrophobic interaction with c-di-GMP guanine base Gua1.	In some proteins, L310 can be replaced with methionine or isoleucine.	[29]
	R314	Binds N7 and O6 of c-di-GMP guanine base Gua1.	In some proteins, R314 can be replaced with histidinehistine (like in PA4781), lysine (like BB_0374).	[19,29,65]
	K317	Binds O6 of c-di-GMP guanine base Gua1.	In some proteins this residue can change to arginine, serine (Vnz_24090 from *Streptomyces venezuelae*) or to histidinehistine (in some deltaproteobacteria) and maintain specificity to c-di-GMP. However, when these residues are changed to glutamine or to asparagine, towards the specificity to cGAMP, like in Bd2325, Gmet_3476, and Moth_2495.	[19,29]

Table S5 - Corrected sequences of proteins used in this work based on the sequence of RefSeq proteins with the name of old locus_tag and the locus_tag of RefSeq are below. In blue are the residues sequence that was missing in the protein LIC_12273 in the KEGG database when compared to the RefSeq. In red are the residue sequences that are missing in some proteins in the RefSeq database when compared to the KEGG database.

Old locus_tag	Locus_tag	Sequence
LIC_10049	LIC_RS00250	MSVQFQTHMQAQRSWETIQDVNKIKYILKEYIHFQGLLVKESPFHQELKPMEI REDGTFIFPIDSTLTNINDELVLYRTLSKHIEISFDVIEKTDTQIICKPLFARIAKA KRMSPRIEGLMGKVIAHKFLIPRKELDIAKVLGTSGQIILNDLNRKVKQIYPSSK LVFNSSKELSPEEELVKKYKKPIYIRDTSTLDSNPEGEFANLDLLPIKETLQEELI LEERLRFFKSSKTRSLLIYPILFKSGGETQIFAMGVIESSEGPISDQVVPLYKEME EIFNSRMGDSNTKSLDKRQNILNISEGGILLEVTDPELIQSFLHKPVFTADITFK MQAPLRFAFHIRHISQVGEIYHVGAEIVGSNDAKANMTLLKKNMNFIKTQ
LIC_10122	LIC_RS00630	MKVTHSCLEFDSIEGLIDFAREFETGSMIRFLSPIEDNSGNVLVKEEVQVKESTL ARLKDIKGQYTPKFEIKLNKELLEQIQNILAIKIVNQLKVTDMKFLKFMYENTN YNFKGIIRNSLLSKKTTLTLLKVYNQNLNFFKYISELGLLSLGIVMIPDTMRFRL LRRYAFTAGILMDVPRIGVDKFTKLPSDDNEKVRVAHKCSDILQKLDLIEFTY GSISNHMPLGMIEDSTSSDKAAPGENIDETFLDDIISNDGESDSKVDGSREDAIP EKSYDIFQALLTDALKLARYIANVSHNAVDKDYVMEELVYYIAYNTSKKYFD ELLANPLVATFKEFEVNVKRLRKIAEVEMKCVYPPSAWAYPKPKSSQVLCKN KVWDCPNIVMGWDIHVITAQEAFGWVGTSLPVDNYPKCRLEEELDEIMVEPE KPKKK
LIC_10128	LIC_RS00660	MRAEIGQKKTTGDIFADKRFYKRFRKNNLVKMVLGKNEILGNLEDMSMIGAS ISSREEILLGERVKFMSPMLSTAIEADVIRKDLIEEKYKYGLVFHNLSDSAIAEIL NKIASAD
LIC_10138	LIC_RS00710	MDAQKDLQKFDFTEEIIQHFKINSVIPVDFYNRNGQILIHKKENADGDDITKLL RFESQGIYFLKSEFEKISGGKQGDGPNNVNGRDVSFAKLVNAELTVDLAKNAS NFLSELKKFPLHGNQLRHLNKSIDGILEDFKSTPDMETGLVNIIEVMSSAGVPM DSEILTKRTVISMAMKVRAGKAFTKVDMEQKKLDQMNLMMSSYLADVGYT QMKIPMERDLKAEEFEYIKNHPIISYLMIANLPDLDDNIKTLVLNHHRPHKGEG MNNNYPQPKVLIHKLNVYKEKYKDDPKKTVLVADIQKQIRNILTNNLPMEDI GVISIAGEFASLTTRQAWREAFDPLVAMKLILNNSFFAYNEKTLRDFYDHIGLS LCNNQPFIREGDFVIVVTQDSNQKVFFEVCIIREMYKTQIRPMLERIGTIKPNFS

		NMGKLRISGFDIASLKLDRRKAVYNLEKNQDPRRIVYVLDSNMDARLYEELT<br>KQTGEIPKESA
LIC_10139	LIC_RS00715	MEFDKMNSNASHHSQSVNRELLEKFEFNSDVIKSFISQSEIPVDFYNKNGQILI<br>HKKSDASEEDVTRLQKFESQGIYFLISEKDKVTKPKDNPDMVHGREVSFTKLV<br>NPNLTVALAKEASELLEELKHFPLTNNHIRLVQKGIDDILADFKGSTDMELGL<br>VNVIEVMRQAGIKADSEMMTKRTVISMAMKLRGLKALSKTDNEIQKTKQLNI<br>MLASFMVDIGKSRMKLPNHTDLRPEEFDYIKNHPIISYLMIGNLSGVNSEVKSA<br>VLNSHRTFRGEGLNNNYPTTNIIIRRLTEYLQKYKDDKTKKILIEDIQKQIHYAL<br>NNTYTDEDPGIISISGEFASLSSDQEWRNSYDALTSMKLILNNSFFSYNEKIVRD<br>FFDFMALSLCENQSVLNPGDYVIVVSTDSQRKIHFETCVIKEIFRHQTRPLLERI<br>GTIRPVIINKGKIKIQGYDPHSFRQDKRKAVFDLNNSMDPRRVIYVIDPELEPSL<br>YEKVDQSFRGTVPRSAA
LIC_10321	LIC_RS01670	MTGLKLRIAILILSVLSFFYYRFIPYFPDGIYYKNRMNSAFTNLNEELRHLETGL<br>GQINSISELENLQLEFPIVSGLKFVTESDLSSRKDPEGKLLKETLKDGTSRLFFL<br>KPSLVFCLPHPEKKKLILAELREDLFRVSFSGVESMLIPDLKFGGYAEPGGFNK<br>GRISFLLIEELSRSENAVNRIEIGSSPFIGYYYATPENSYGFLKGILILKPGNDGLF<br>FLFLSGFLILLFLIDFMIRILRIKRNFFTHKEGKEIQEIIGQISKRIDALQTAKQKAI<br>ESAQKSEVEEIQTLTSEEVDFDLQNVPIPMKEVKQEDGPSIFVLPFELKREGYV<br>SPAFLRDPEKFKEPEPIAPEIEKKRSEIFTPELQDLISKVNEPIREKPDIQIAKQEIE<br>PEPISTNHTELGPGYMKWLNTLPIRERRKILEVLDELRYGLESEYSFILKYYISV<br>FLDLKLYGFAIHYYDRRNGSYSPFVTQGLRERTSGNMIFLYDDQYIGKESGTY<br>SIIEITDERKMDRFFRKKFDPIDLEICTSILTIPLSNFGIPFRFFLFFKDPLTKENAQ<br>EIENLIFHSLEPVIPAFEEYDRKILGELFRDKRDVVSSRVHLMRIATDGERGLTR<br>SFKIEFHGKNFKTLESLRKKTMSQISEIIGPEDICFGIGVGAFGLYTRKNLEKQIR<br>SLIDQTGNPYDFVEDIYPENGKNLFIYL
LIC_10641	LIC_RS03310	MRSPVSRISVKGFLNKYMLYSQDTSNLLSYGENNYQPHYQPILEVTNCNIIG<br>YEVLGRFYFPEKNEYRSLGYQFHNPELDAIRLIQIDRLIREKAIRHLKDSGLRTK<br>LFLNMMPNFLSMIHTGDVLDLKRIHVLNLIEKYDIPPSEVVLEITEDKFDGNIEK<br>LLSIVNVFKDYGFKIAVDDLGVGFSNLERIGYIHPDIMKVDIKIMRESLNRRSFK<br>NVLSAIADMSQKLGSDLLFEGIETEEELHLALSMGANLLQGFYFSRPQVEFQD<br>KKQFNRTLRDTLEKFSGLRFMELLEEFQKGQSVIDSLGEKLEALRHNGKEDLP<br>LVLHLMLSELPTEILSVFACDIFGYQITPTYFRVHPGEEWDSDLTEIGNNYAWR

		PFFIRHKAKVVQNAAKWTVTEPMYDMDLHKQVVIFTYTLRDNYILVIKMDW<br>EKEGGGLKTEDRRQ
LIC_10996	LIC_RS05140	MKEVFDLPPNADLWSSKSTSTIDTQTLFSFNEKYEMALEILTKFENLSIVDSSN<br>LKISSTQNYDGEILPQLTEILKTGDFHTEYQPILSLETGKIYAYEALARFRIKEKN<br>ICPEFVFNELHQDPDLFFEFEKELKRFQIQNRPQGKHLFLNLDPHVCKNRSQAA<br>EWRKLLSKEKDIVCEIIENTDSTLIENTRFCLDVLRRENVPIALDDVGGDQNLF<br>CFNFLEYSKFIKFDKCWLRLFEIKPSYKNIAWGFLDFAKESNILCILEGIETSEDF<br>LMAAEMGFPLAQGYLFQSRNVLV
LIC_11125	LIC_RS05800	MDLENEYNHEKFFNYSLDLHAIQKMDGIILQINQSFQRIMGWTNEDLKGRTHF<br>HLLHPEDVESSLKEFEQLNEGVSHLSIQNRCRCADGTYKYFSWTAFPDLESDRI<br>YVTGRDITDIIESNQKISKLASDLEEANNKLLEQASTDPLTKLKNRRSFNEEINH<br>LIRLGQHQEKSISLMMIDVDHFKDYNDKFGHPAGDRILIRLAEVFTETLRSCDL<br>VARFGGEEFVVALSDTNEEKAVEVAERLMANVKKKSWENSPITISVGITTLNF<br>NGPVPIYHTDLSTGIIEDADRALYRSKANGRNQATHSSQLKTENQTSVRKN
LIC_11126	LIC_RS05805	MEKRHMSYQNEYNLEKFYNYSLDLFSIQRLDGTVISVNPSFERILGWKEEELL<br>GRDPFHLLHPEDLGSAKEFEELDGGVPRSSIQNRIRCADGTYKHFAWTGYPDL<br>EAGLVYVTGRDITETIEANRQISQLATELKEANDLLFEQATTDPLTKLKNRRAF<br>TNDLHSLLRFMQQEKSFLSLLMIDVDHFKTYNDQFGHLAGDKVLVCLAKVLE<br>NTLRSNDLLGRFGGEEFVAALPNTCEKKSIEIGERLVNAVREFNWEYRSITISV<br>GISTSDFEKKQLDVDSTNLIEKADKALYQSKVNGRNKATHSSQIY
LIC_11127	LIC_RS05810	MQYDYEKFFHNSLDILVIARMDNGHVIHVNPSFERIFGWSEKRLLGLGSYEFL<br>HPEDLPSTQEIAAKLATGAPIVSFENRYRSIDGNYRTLSWTAVPEYKSGMVYAI<br>ARDITEAIESNRKISELATELKEANNMLLEQASTDPLTKLKNRRAFNAELNHLL<br>QIAQKQSNPISLLMIDADHFKDYNDKFGHPAGDQVLIDLAFLLTHTLRQHDVI<br>ARYGGEEFIVAMPDTSEVASLQIAERLIHVVGEFNWEKRPVTISIGAATLPGEK<br>QKTSLEENRHFINLIESADKALYHSKINGRNQVNHSSRILPCKEA
LIC_11128	LIC_RS05815	MTNFEKEYDLEKLVNNSLDLLSIQRLDGTVLQVNPAFERLLGWREEELIGRNP<br>FHLLHPEDRESTFQEFKKLNQGLPVFAFQNRFLCSDGTYKYFSWTASPDLSAG<br>LIYVTGRDISDLIESNRKISLLAAELKDANDKLFEQACTDPLTKLKNRRAFNEE<br>LNSLIQLSQKQLSPLSLLMIDVDFFKDYNDKFGHPAGDKVLAILASLLAKTLRK

		DDVTARYGGEEFIVALPNTSEKEAIEIAERLVKVIEESRWEKRSVTISVGITTSQI NPSNNADHSIDIIEEADRALYHSKVNGRNRVTHSSQIS
LIC_11129	LIC_RS05820	MNFENEYDLEKLVNNSLDLLTIVDLSGNVLLVNPAFERTLGWKKEDLVGKDP FHLLHPEDKESTYKEFEKLNQGLLTLSFQNRYICADGQYRYFSWTASPDLVSG LVYVTGRDITDVIESNRKISQLAVKLKETNDRLFEQASTDPLTKLKNRRMFNE ELNNLIHACDKESHPLSLLMIDADHFKDYNDKFGHIAGDKVLVELASILTKTF RKKDVLARYGGEEFIAALPNTSEPEANQIAERLVQTVREFSWEKRSVTISVGIT TYNFNPTSKSINSEYLLNLIEQADKALYCSKVSGRDRITHFSWVQNKKSGSI
LIC_11130	LIC_RS05825	MEYKNEYNLEKFYNYSLDLFSIQRMDGTVISVNPSFERILGWKEEELLGKNPF HLLHPEDQETIVQEFQKLDGGIPRYSIKNRCRCADGTYKHFAWTGFPDIESGLV YITGRDITETIESNRKISQLATELKDANDKLFEQASTDSLTKLKNRRAFNEALN HLVHFSQKQKSPLSLLMIDADHFKDYNDTFGHLEGDKILIILADLLTKALRKG DVLARYGGEEFIVALPLTSEDETIDISERLLQTVREFNWEKRIITVSVGAATYDF NSNSKNINLEYSINLIEQADKALYCSKVNGRNRITHFSKKYSLQQN
LIC_11131	LIC_RS05835	MYSRNRMKYEEMKYDIEKFFDYSLDMLCIARLDGYIFRINPSFQKAFGWKSE DLLAFGSYTFLHPDDVEPTYQVVEKLKKGTPIVSFQNRYRCTNGEYKNFSWT AFPDLRSELIYAIARDITEIVESNRKLNQLAAELKDANDKLFEQASTDPLTKLK NRRTFNEELKDLIVHTNKKRGFLSLLMIDVDHFKTYNDQFGHPAGDQVLIRLA SVLTYALRVSDLLARFGGEEFVVALPETGEYKAIEVADRLVVTIQKETWENRP ITISVGIATLDFSLPVSTFRDTDFSTRIVEEADRALYRSKANGRNQATHCSQILL
LIC_11167	LIC_RS06010	MKIQYLGIIFIFFSCTFDVHTNINKNLFTGDTLVFGGISISLLLLFGLVLKESSQK TSGRSLSEQNEKKTDSAMEIVNAHDTISDLEVGLEKIKAESSDLKNETKTEKNI NSEILRTNQTRIDKIWKREVPTHPELIRAHQKFLEKLGSKLNGAEIYFLSGEVSF LLASRKGKVFHIPESPEENILTKKSIEEIAEGRISFSDRSIMIPLGTAQPFGYLQIS QNEWSQLESREFQSWKEEYEEQVSIQFESSDPETGFGNLHAFELDKVRVDENI LILASIQTDSPYPYVLKWISLWTSKLLHKQVRFYRIRKDRLAFFVSPEEWDFFS KNLNELVESLQKENHLVDLNLGVSILEESREEWSSNARKALGLSIEEGPNRYIC L
LIC_11189	LIC_RS06115	MKPKFPKYIVTDLDSIVKRLGPIISRLEIEFLDFKEFFQSELFRNSSGFLNVIFYLT VSDLKKTQKEIHQQFQRNPLILSRFILNENLDYVHSEDLKIEEELIFSVLPESSSD MVFNKTFLNAFTQLQMITDQFDLQHKVNTTKYEISKLTRVGISLADEKDINKL

		LREIIFSAREIAIADSGSLYLVEKDELGLVRNLRFKISSMDIDTEEFLLPINKSSV<br>AGYVAATGKILNIPDVYNLPEDSEYKFNRNFDVLSNYHTKSMLVVPMKNHRN<br>EVVGVIQLINKKRNFNQKLTLEQMRGNDVFPFDDYSAQLVMGVAGQAAVAI<br>QNNHLLQEIEALFEGFVTASVNAIESRDPTTSGHSFRVAVLTVGLAETVDLIRE<br>GKYKNTKFSKEQIKEIRYASLLHDFGKVGVREKVLVKSKKLEDYELELIRWRF<br>EYIKKDIESRILQKKTDYLKKHGAVGFADYETSLEFELKVEYQKLDQMFQIVV<br>DSNEPSILEESNFQKLEEIAKVNYSTTGGEKLSLISPYEFGFLTIKKGSLDFAERK<br>EIESHVEHTFQFLSKIPWTGDLKMVPSIAHAHHEKLDGTGYPRGLTADSIPIQS<br>KIMAISDIFDALTDKDRPYKRAVPIERALDILQMEARENHVDQDLLKIFIEGKV<br>YDKLYYSGYLR
LIC_11203	LIC_RS06190	MNQTTLPNLLILDDEEEIAGILGDLARQCGFEVTITHEAGVFFERLNEKTQYIIL<br>DLMIPDVDGVDVLRMLSGKKFSISVILISGADRRVLQSAEALAIQYGLKISGVL<br>EKPIRIREYQKLLTGLISEQKNESAHSFTSGKRIVHEQASVIGPEEIEQGIRENQF<br>VLFYQPKIDFKTGTVSGFESLVRWSHPERGLIFPDAFIPTLELYPSLMDAMTEK<br>LILQALSQCSIWNKQFPGIAVAVNVSPVSMNKLILPETIAKMIENFGLKNNQLI<br>VEVTETQLLENMTSTLDILTRIRIRGIGLSVDDFGIGYSSLKQIHRYPFTELKIDR<br>SFVSVAPYDKEALFICQAAIDLGHKLGMTVVAEGIETAEVGELMKQQGCDKG<br>QGYFYSKPMPANSTLQFLAGFKS
LIC_11247	LIC_RS06415	MGLLERVSKLVRSDSSRISTSTSSAEEKKSLLKKSEAFQRKPGFLQKALGMRK<br>ELTEEAAISVPSFENKLQASSKESVDFEESNSEELSIPELDEISLDANEQEEFSDS<br>DLSISEDLVEKDSAFDEIDSQELSQDENDISIDDLFKDETTSSLDQEIVDSDKLSI<br>QDQNEKETSSNDDITSLEEAPFDDWVKEAEQEARRTLPKKDPNVSASEKDGFL<br>FDDDSNFATSPIDLQIASRKKLENYISVFEISKEIGISTDFANFFENLLFSIMGQIG<br>SESIGIFSSKNGNQDFFRLEDYQGENFNQEWTISSEEEIYHAVHNAGSVLYAKE<br>LLKPALPAKEREIIQQSEAELLVPIRTTEEFFGIILLGKTISGEDYTIEDLEFLKIIG<br>EIAGSVYKRIYDTELLHQENQNLKETVRANELIISLARDFSSVRSMDEAYDKLF<br>SAFREELKVRRATFMILENKNEFRVFASNLLTPEHVGSFRLSLESSIVGIVSNIP<br>GVFRIENFRKHPELVQKLSNDELGLMTDFIVIPLINLHWLVGMWIVHETEVPW<br>TDSDRETAVGISEVLAPVFSNLLLVQERDSVFKDPFSPVEEKIEEMILKSTRLGN<br>CFSLTIFKIQNISRMVKLKGSGFFVNYSEELRKAIQDNLSEVDFHYRIGQGKYA<br>MILDGKDREETQVLIRKIKNRLVEVDRRSKDFQTSVVQHTLCYPADTKEKERI<br>LELLEES

LIC_11300	LIC_RS06690	MRLSLGNNQKIKLLARRVFFNPFPDDYLRIYQPDVRRATVIYFFFCIGISFLSFL LPDGASLLGKENQILVYSRLTVLLLSAFFAFLLIKWRPFFRRRIERYSILSSGVIV FSILPYVFLDPGRMGLYFHFYTTLVVSGNILLWLTGTTVVIFNGLFYFSLVLCT SLTGNKNVLQHDFANVLIYLFTGIFGNLLINFWRVMDHRAKKKLQKAVSKLR DKNIQIEKISKVDELTGLYNRRYLIEQFELFLKRAQRYRFSLAMIILDMDYLKEI NDSYGHLAGDLSLRTISDVMKQRVRATDICSRIGGDEFCILLDAIKKEDLVQLC ENLRMEVAEKELSYRTQKGSPVKITVSIGACIFGPGEEFSFDDIYHSIDSALYES KKKGRNRVSFIEPIRYFPREYPGTTAAS
LIC_11444	LIC_RS07400	MDSILILDDAQENCMLMQGILRKSGYKNTFTSQSPDEVIGWLNLKNQEPPQKE CSLILLDILLPGITGLEILKLIREKEELKDIPVIMITALKESDVLQKAFDSGAIDYV VKPFDAIELLARVRSALRLFEEMTRRKEREKELENLTDQLQEVNAYLVAISRT DSLTGLYNRRYFDEILSTEWKRCWRTEASIALIMLDIDHFKLYNDTYGHQGGD QCLKQVASAIRDCARRAGDVAARYGGEEFAIILPETTESNAVVVSRNILENIEN LGIPHSASKTNSIVTVSIGMATLSPSPENSITELIERADKALYLAKEEGRNCLRF YPQND
LIC_11447	LIC_RS07420	MRKFNGTEFYSFLFKSGCTMIEKIIEDFKDSVKESVKSVRIPLEVHSKTGNYS AEIGGWEGTSMSLNLERFNLDSVGEPATLEVKIPFWKDPILLHGTITEKKIVRS RSQDRMTDNVLLFESDAIGLVKKFLPKFPLDLKEWKLLEFSKKAVLPSYFPKN LRMWELI
LIC_11563	LIC_RS08010	MKKFAVSELKPGMRFSKPVYLDKENLFITSNTPVTDSDLDRLNRFGIQEVMTA GELLQLESQYDPNVLETSLDDMIVNTVVDDELQPLKAVYDNLNRIKITFGNLF RESTQIIQDVFKKTMDEKPLEVTPVREIAERLTDFVRSNQNISYLILANNPPGYY LYNQIANSTFYSLILGKLLEYSRPKMIDLGISCLLADIGMSKVPAVISEKNEQLT DEEFKTIMKHTILGYQILSQKMKLKNNLAIVALQHHERYDGNGYPQKLAGNA IEEQARIYSISDNFSALVTNRPHRKKILPHEAIKSMISMDVGKFDLKLVRTLLNH LSLYPVGSCIELSDKRIGVVLGPNLDKPIRPFIRIIKDEYGKMVRNLILVDLLKET NLFISRPVDLQEISA
LIC_11571	LIC_RS08050	MKTLGDILIEEGIISEKDLEDSLKVQKKNNLPLSHIIQKKGIAGEADILRALSKL YQLEFREKLEFTGMEEIFLQIPLKLIQKSRIVPFSLSKKTIRIAVSDPSDLHPMDD ARNFLKGYNVEFILAPEPEIMRIIHSHFDNTSSAAKEMLNEMEGSFSELAEAFE NETLDLSDDAPIIKMVNVILSQAVNERASDIHIEPYEKSLVVRYRVDGILHNVL SPPKSYHAGISSRIKIMSNLNIAENRLPQDGRIKLRLAGKDIDIRVSTIPCQFGERI

		VMRLLNKTDQKYSLDTMGFYPELIQSLRSLIYEPHGIILVTGPTGSGKSTTLYS<br>ALSELNTEERNIITCEDPVEYQIEGISQMQMQEKIGLTFATGLRAILRQDPDVIM<br>VGEIRDEETARIAIQASLTGHLVFSTLHTNDAASAATRLIDMGIEPYLITSTVLG<br>FMAQRLVRVICTQCKETYKPTTSELESIGISKKFLKNGNLHRGKGCSHCMGTG<br>FKGRIGIYELLLVNSPLKQAILHGKDAGQLNEIALEHGFRTLKDYGIRKVVDG<br>VTTIDEVLRVT
LIC_11706	LIC_RS08705	MESSIKPITHKYKITEDPFEEFSDQMIRDIRNSLSNEIVVTHFYFQDLYSYFKLLG<br>EQKSGEIIKELKLIIQAHLRPYDKLYVANSRSMLTLSPDCKIEVVQGRFNEVVF<br>QVNHLIVDYEIQFMELDSPIDSIEPIWKQLLVSFKST
LIC_11920	LIC_RS09795	METIQRKKREKETISDPTKKFHIISKFLVQTDIIAQTSNSLKQIVKILQVSKDATK<br>ILVQTQTPNALPLNSHVTLAKLLAKYVELSCEVIDEKPNNQFILSVSEISIASKE<br>RSLNRIVPPEGTVWITNIRTSKTTIDANLFNIPTSVKVNFADYETKLKSKYDFLK<br>VDVFGTIGDKFDLVKKTRKILYISNTQKEQSYAAYNQEDFIDYAAELGDEEDV<br>HKRIIEYANQKIKSELIVPVIYLSHEEQAIPIGFVHAQNRSREIDILEVMEIKTLTF<br>EMVDRIRESNTILVKERFPIVNISTGGLKVKINHPDLNQDFIKRAGFTFDIFFKM<br>QAPLTAFGVIRSVTKDTEGNLYVGLSIEGNSYRPGERKKYIDNVNRLLVEANPI<br>QSQF
LIC_11921	LIC_RS09805	MIGISPKTKLEWDLWFQSGEIVPFFQPILSVERDSIFGYETLGRFRDQSGKIHSL<br>GPFFLNALSGVEDLQERREIYILKRDIDRSIRKKALLHLLKNQNRFPEAKLFVNI<br>SPAYMRDHIEEEEVDPYTIRLVKEFGLDPSKIVIEIVEEHFDGSIESLRPLISRYK<br>EEGFLVAIDDLGSRSSNLDRIGIFHPDILKVDLQMLRHSVISRNFQEILFTISRLS<br>ESLGCSLLFEGIENETELFQSLTYSARFLQGFYFAEALPNMVGQEELKLRFSAV<br>HECFLNHKRNQLVKRIQLEKELEEKLDLSGIIVNAEEELCSIQIQNPNLLSNFVF<br>RIYATNLIGSQVSPNYMKIGNSSIDIDSSFVGRNWSWRPYFLEQLYKSMKDTR<br>AEWIISNPYYDISYGILLVTYSKKISEQNVLFVDAQVLEY
LIC_11993	LICRS10175	MHYNRIPNTVTVYWSKLADQSLRLAENILKGLLHRTDSPIEPGTVLELKLGTIS<br>LSGGIQIPVKVIRCEKISEAEYDLYLNYTERDFNKIQEIEDLIRDLS
LIC_12273	LIC_RS11610	**MISKENDPLMIEYLEKKIYDQKQLLEISKALNSTLDYKYL**MDAILNICLAQL<br>QTLQAAIYVSPEVDSDFFELDPSYKGFDLSENEKSFRIKTNAALIQFLETRMKA<br>MTINQIEENMGRAVNEVDFLRGIGADLIIPLNAKGKVNGLLVLGEKMTMNEV<br>QEEDRDFLTTLSTLAGIAVENSRLYELATVDMMTGLKVHHYFQTKLKEEMDR<br>CRKKKSYLTLLFTDVDNFKKFNDTHGHQAGDQVLIEVAKQLIRQAGKHDIPA

		RYGGEEFCLVMPGADLERGYEMGEMIRKAVESSSVKNPNGGPDLKVTLSVG VSEFWPKDKNNRDLIERADKALYTAKNSGKNRTVCYKEN
LIC_12491	LIC_RS12745	MSGSPDKQPRSHRYHPGVYADYIIQIEFGLITLHAKIGNISETGICLILNGEDLN MTESVYGSVIEKKSGKRIEFEGDIIWVGEETIDDKIRFVYGLKFREPLILTESLVL INLSLQNP
LIC_12505	LIC_RS12825	MDGNINSQEENKDSRPIILLVDDEPIILRGLKEQLKLAFGKDYDIEIAEDAEAA WEILEEYVEKGIDIPVVVCDQVMPGVKGDELLIRIHNKKPDIRKIMLTGQASA DAVGNALNHANLYRYLSKPWDSNDLILTIREALKSYFSDLSLVELNRKLETTL LYNRETGRPNFESLRKILEEREAKEAQSTLAVIRIESSTATTHHFGVGVYHKVL NQLLAALSSFMGDSGSLFHLYQDEVAVLSNVDEDRFHSLLVAFRILLRSEYIQ ADGVAFRVNVSIGVATHQHSLYYKARIAMMHAAQNVELELMNYSQAMEEG DQYQINLILGRKLNDAIHVGNVIPYFQGIYDNTLEKITKFECLARIQDGDRVYS PASFISIARSTGIIRLLTPIMIEKSIRYFAQYPEYSFSVNISESDLEKKGFASWVISR LEHYKIVPERFTLEILETDRLRGGERGLETLKELKESGCKIAIDDFGVDQSNFER LMEIDPDFIKIDGKFIQGIHLSRTPYLLTSAMTEMAHRIGAKVIAEFVAGKGEFD TVRSLGVEYCQGYYIMQPVPEIFPIPQVSV
LIC_12546	LIC_RS13035	MTMSGIKLKERDLDVINSPEQRKHIIEKHLLNQSLRIKGDLNRETITIQRYVDS GEKIIAGISDETHFSENDEIVLYKILAKYVQLECSFIRKTRPGVAEFSVNKVSIAK SNRAFPRYSVAEDAAHVTNINSSKTVIDASLFNIPTLVKVSFEDYKTKLKPDQL GLVEIDVFRSDQDEKFELIKRTKKYIHIENTSLEESYKSKSENQVDVEDEIHEEI PSLMRKYKDEKIVSEIIYPIIYINHSRQSIPLGYIWVRNKEKTLGNNTIEKLAELS KEMVARIKESNTVLTTEKFPIIDISNNGICIKITEPHLIQTLPKHTGFVFDIYIRMQ GYFKVFGAIRWLSYDEVGSLILGMELVAKSSFPGEREKFHRNVELLGQGKFTG LKTHAI
LIC_12723	LIC_RS11610	MISKENDPLMIEYLEKKIYDQKQLLEISKALNSTLDYKYLMDAILNICLAQLQT LQAAIYVSPEVDSDFFELDPSYKGFDLSENEKSFRIKTNAALIQFLETRMKAMT INQIEENMGRAVNEVDFLRGIGADLIIPLNAKGKVNGLLVLGEKMTMNEVQEE DRDFLTTLSTLAGIAVENSRLYELATVDMMTGLKVHHYFQTKLKEEMDRCRK KKSYLTLLFTDVDNFKKFNDTHGHQAGDQVLIEVAKQLIRQAGKHDIPARYG GEEFCLVMPGADLERGYEMGEMIRKAVESSSVKNPNGGPDLKVTLSVGVSEF WPKDKNNRDLIERADKALYTAKNSGKNRTVCYKEN

LIC_12994	LIC_RS15405	MEYIEKTERETDTITSAEQKDHVITKYLLDKELVFKIDPFDRKAVIKKVLEEGE<br>KIIIQVSKEEDLPKENSFILYTILAKYIQLECILLQKLENSLFIIKVEKLAIARKNR<br>ETQRFQVESDTMYVTNVVSSKTVIEANMFNIPTLVKVNFEDFKNRLKQKTND<br>TVNIDTFGPGLNHKFEIVKKTLKYLLLENTQDPNSYKNNSPGRIHYEKEIDDDL<br>DSCIQEYKDQQIISELIVPIVYINHSEEQIPIGYFSIQSKEQSFTEKDIQEFQTLAN<br>DMIERIKESNTIKTSEHFSILEISKGGIRVKIENPHLIETLPKQNDFVFDIFFKMQA<br>PFTVHGIIRWLTLDENGHLILGIELAGKSDLPGERARFESNIELLASN
LIC_13120	LIC_RS16055	MGIQNLSPSLQKSLLYFSDQSAEGIVVLDRDWKTVYENHKFQNFWSFPNFQV<br>LYEKIIPLLKSKKKERFENDISISNLLQEIEDSKDSFLNETNFQLQLSVHDFEDSY<br>MIRFKPQPNAQPKIEYETGHWDRNTNLPNQKYFLNHFGNQISEETTFLEEHFSF<br>LISISNPDAIISQERNSYFESIYVKVADRLKKHLNRNDHLFRIESDKFLISSLQIDS<br>ETKAEWFAECIRMLFDFPFTYEEREFHLNVNIGYTKFDLKSGSDMNALGILKE<br>ALHRSCLIGPNSLFYYDQNAIITTYEKAKIEIDLRKVLNRNELEIHFQPIIDLKEN<br>RFFSMETLVRWNHPEKGKLLPGSFISIAESSSFIKTIGEWMIWETFQYYENSILR<br>SENISLSLNISPKQLGDKNIFPLLKEASDYYKIEPNNIILEIVEDSFDFRKSQISKVI<br>TSLKDYGFKFAIDDFGKGYSSLGRLIHLPIDYIKLDKMFLFNYFQTSTRAVITS<br>MVNLVQAMGKSIIVEGVENETQHNLLKELNCDFGQGYYYSHPMEIELAEKLI<br>QNKAIPAT
LIC_13137	LIC_RS16140	MTLKDSKPELIKLYSSLGKIITSSLEQQEVLSAVMEEVRLFFSPKNWSLMRYDE<br>NSEELFFLIAEGIQFNHIRSIRLKSGEGIAGSVVQTKSPIFVENVKNDPRFSKKVD<br>EKTGFETKTIIAVPMIFRGEVHGVIELVNRFDGSSFSPEDLVILQTIADFTAISLA<br>HSDQYEKTKILAFRDSLTGAFNRNKLNHLKEKWSNRTMENQIALVALLDLNG<br>FKMINDTYGHKTGDQVLCYFADILRYVIRGTDKIFRIGGDEFLILVRHENQEKI<br>LQTQNRFRETMSVLLKKCKENDPPFNFTWGMSIGSIQKLDELIHEADLSMYAS<br>KG
LIC_14002	LIC_RS11585	MDKFINDAEGIHKILQSLFTRLPVVILVDNRPLPVRVAGLKDSFRIVVTLPPGTP<br>NEQSRKLFLVHNNHRFAAFCTVELHNPSNSVELLLTSAIQVTIAQRTEKRVHID<br>STSQITLTNIINQYKVRKAVGFADKKIDGIVKKHAKLLKETYPLSSIFFSDKMD<br>NRLRLMYNFDKPIYILDRYAKSDGSAGFQFLTFSEYQKLIAVNNLESGIVSEISI<br>MIRYKGYTPLGYVQILSDKELSASDFNTANITANSISKEIIASGFFQESKEKCNV<br>DNISMQGVGFFHHQSIFFSRSFAVGETILFDINFSAESKGTFRAVIRNITNTDKM<br>FRIGCEFFNLNEREENMIQTYIDSKENQT

LIC_20106	LIC_RS18525	MISEYNDLYVENKALSSDIYSYSLYSLEGKQLYSEPNYNSLLNLDPKLKKAVE<br>RFIDYGNSNVSTFRRFKNDRSIQLMVRSENDRIYKVSFEINTKGSYVLAHIESTT<br>RNLSSTEKNRIQRFRSLKHDLVRTLKLEKTYFYLINIEIYNHPFLHKYETQIYEA<br>VFLDLHTEFLTITNSQNVGLRISTNQIFFAYQINKPDVDINWIPSSLISYMKNTIQ<br>IDNHEFHLKLSIGGFHTSEANTSPLNILKGLKANLRKVIDSPFSRYATQNQNDSS<br>NLIATYLSLRNSVHKKELLLYYQPIVNSENKSLHSLEALSRWNHSAKGMISPDI<br>FIPLAEESGLISSIGTWVIQNALLDLSQIRKNNSFSSDPIVSINISPFQLKNPEFAD<br>NLISYFSQLNLHPSSVVLEITESRYEETVLIIEQMAILKRFGFQIAIDDFGIGNSNF<br>SRIEKIECDYVKLDKSLIIGVDTNQSKRSVLKAISQVLFSLGKKMVFEGIENAEL<br>ESIAIDYGANFLQGFYYGKPTQITDLSSFQFQDHFLK
LIC_20136	LIC_RS18685	MFFLAQARPVLIWPEFSWIPVINGTIFVALVLLTGYYLEKRFRNSIERRAALRA<br>KILKKLPLTYMHGRDVVQIHSFLDHVGVSVLQKIAESSSWFQEVFLPELAIYLA<br>HQGELPAWRDAIIFKRLQHLVHDLGPHPKKILPVVFLTDDEEAFPGLLYSGPPG<br>SDFVQKSIHAKVFTKKLYHSFPVSTGDKIHVLYSGEDRDWIRFDAKIYSLNGN<br>DIGIQVETAPEKDPEKTRAWGGIQMGGGILEDSTLPDEFQGSLSQILNYGSIGTS<br>GTSEIQRRVQAFKEHPGLVRKEHKPEEIQTFIELYSACYARYRSDISPVPKPVLL<br>FLYFFYMDENLLSPTRIVQLYETLEKIKDTQDPYPSDHKLAVYFLPEWLGLILS<br>GKKTPSRNHLAQSYEQVRASMIRKTGTDEYAGDSGIEDLLHLLDWELSNLLF<br>NGLIGVSANPNLAYPILSEDQMYGETDAFLVTREKINSVVDHVHKIDKHLFYR<br>QISFEPEQTPGKPELAMKEICPDCIILPVFGSRGVLWQEITSGLSSRGRLVFPQIL<br>NENMTLAITRTLGEFRWEMERTVRGRKWKDSSPPSLTSEYYLYLENYRKSPA<br>LTPDAKKGIDQQLLKYRKNLKDMFASDYSYWILFESSGKLRLNRVARDILNR<br>YVPFSPQLRTELQKHPILKESMDSFEAKKRRLVSGIKKRYNPYFQAGNVPVEV<br>LETIRFFEEM
LIC_20173	LIC_RS18880	MLLYLGFEELLTSFLKFVTTLFAAGFYWFFYRNTYYHPNRKSFDLSAIFCGVL<br>TVGLAIFPEILAKQYIDKNSYFERAFPGSSLLEEVPKLIVVLWYFRGLKSVYNTS<br>DGIYFGLTLGASFGLLENFLYSTTVDFWPLFLRAVTSLPIHTFTAGIYGFAVMQ<br>YYHSRPSSFNFLGIYYSLFGCFLLHGTFNYILLMDGDLVVLLPFILAIGFFVLEY<br>LLTISQNILPIEVLQSIGLFRDDYTVISRFTRYDSWMRSSQSQAQKVESIPLFRQL<br>SKVKVFVSVFLFLIPTLLYFIYSTFPELIPLLLGGIRTSEFIGLFLVYPIWLSVLILF<br>RGILNPKFFRERILKIPLFIAVTIVQEEREYHSLAYSLSGKGFYSPVEKNLIIGDR<br>VYVTFYVAGKEFSNILAIPVWLNVREDDPEFEPGAVFIFVTPPWRLLFWRLLV<br>RTKQQFQNLIHQILHPIESSHSI

LIC_20180	LIC_RS18920	MGNSEFVQNTSLNIKPVILIVDDEVIILGSIKYAIQLSFGNRFDIEMAEDADSAM EILARCKSEQIDVPLVICDQVLRGKSGDELLIQIHQEYPQIYKVMLTGYASAKS LGNALNKANLYRYLTKPWDSEDLILTISEAVKAYFQNRKVAELSSKLEETYLF NRETLFPNFENLKRRIDRRLIENENSILALIRIESFGSIAENFGIETYRKMLFEFLS VLDCFIGGYNGEIFHIYDNMIAVLTKIEEDRFYSLLSAVRIFLRSECIEVDGISFQ VKISIGVSSDQSDVYDKARLAMMTASNNSSVEYIPYSETTNKVDRIYANLKLG KKFNSALNAGSIIPYFQGIYDNKLNRITKYECLARIMEGDQIYNPAMFIPIAKST GIIRLLTPLMVEKTFKYFSKHPEYSFSINISESDLDKKSFPLWIMNRLLHYRISPD RVIFEILENDHWNGSSNSTRSLQELKEIGCKIAIDDFGVERSNFERLIEIQPDFIKI DGKFIQGIHENQTSYRLAAAITEMAHTIGAKVVAEFVSNEEELEAVKSLSIDYS QGYYLMEPAEDIIYTETVLNLA
LIC_20181	LIC_RS18925	MMKKNLDFLFDGTYVIFPLSDLFIEVIQLQFKTQINSQIENVRVKKRIIFSLLAII LIFMFLFLIYENFRMEKEIDRVVGMRARVMKDYIRKVGSQTRALGLSITDYLT FYENTPINPDLIKKFKNYPNLNRFGIPHISRENQEGSGPDAGTLTAVGSVHKLN PSLLKEIEAALNLRGQFESLTEKQSEVVWAYYLSERKFLYITPKFKDENYYFTD DLYAEPYWTQANPIANPAGRQIITDLYEDIAGKGLMISVSEPVYVKGKFMGVA AIDIGLDAMQRILESGDCIGRSMLIDENGKIVAKPDNFALNDRLPSSVFSSILES KKSFFLQKGSFWIGVKIKEEEIWLVHEIQIFEYIIYIIKNLLPFWVLTFTFFIVLVL YVKLRSSMNQVSKLIHTDPLTGIWNRRGFLKLTQRSIALENRHKNEWAILMIDI DHFKQVNDRFGHDAGDKTLIRVAQILSRSIRQTDVVCRWGGEEFAIFLCGVGQ EISINIAEELRKEVENQIRLKDGNPVTLSIGVSGGKQNLEEAFTNADQALYQAK TSGRNRVCAFNFLQSSLK
LIC_20182	LIC_RS18930	MNVYQQNESRIQKKKILMVLAVIPILMIGTFVYEVKQIQSEIHRVVWMRARVT QDYIRRISNQTRALKLSISHSMDYYEDSILDKRVLHKFKKYSSLKIFGNRNQIK TNSSTSEFLATSASITPFFLREVEAALGLGGQFETLVETETQSEVVWAYYLSAQ KFLYFAPTLKPYKDIFNEGLYQRPFWVQATPKTNPQRKQVISELYNDIGGQGL MITISEPVYFRDQFIGVAAIDIGLDAMRRVLAVGDCIGESILIDENNKIIAKESWI DINDSTIQLPSGPSEKIFLQEGYYWAFFEIKDQEVRLVHRISAVEFLFSVILNLLP FWGLICTLGIVSILYIKLKASMEQVSKMIHTDPLTGIANRRGFLKLTQKSLVTIH RHGQSWTILMIDIDHFKQVNDRFGHDTGDRILIKVSQILSANIRQTDVVCRWG GEEFAVFLFGVTPEDSINIAEHLRKEVESKVVLKDGKPVTLSIGISEGKSGKSSG SGLENAFACADQALYQAKTLGRNRVCVFDTTTEMF

LIC_20198	LIC_RS19005	MTTYKSPKILSFDEGEIEQFKNIFINENRGKPIVLLRFQDIKSLSFLDFLQLVPIKI<br>SELYPGAEGHYTYYCYGDKKSLLIGVAPLRVNGSNGFLNFDSLLGKLREISIKS<br>GSMNFDFGIARTQCNYISYVDEIFHELEVSSLKNLQDNLIRWSWTYLNRVNDY<br>FAGEKADAVIQPIIHYNHKTQTYSMKGGEVFVGGEAYAGYADLIRDIPHDQDL<br>NRIELLILEKLTMCCNGAPGLLKFNISPQTLIDTFDTDEKVTRFHNLLLDQNLNP<br>FLVRMELIEKPYEEKDVTLKNVCKRFWNFGISFAADDFGVKSQSHQIVLDLGE<br>MIKEFKLDPISFKFKADQDLTKFLDNLAFIDYCRRLSDNREAIITAEALEDIDSL<br>NFLLTHQVYYFQANLFCKKISIKEYKEIFKDMQDLPEAVVNQILTSEELLLQLK<br>EKGNIFELARKLELVP

Table S6 - Orthologs proteins presents in the genomes of *L. interrogans* serovar Lai strain 56601 (Taxonomy ID: 189518) [68], *L. borgpertersenii* serovar Hardjo - bovis strain JB197 (Taxonomy ID: 355277) [69], and, *L. biflexa* serovar Patoc strain Patoc 1 (Paris) (Taxonomy ID: 456481) [70].

Protein	Lenght (AA)	Domain architecture	*L. interrogans* serovar Lai str. 56601 (% identities, % similarities)	*L. borgpertersenii* serovar Hardjo - bovis str. JB197 (% identities, % similarities)	*L. biflexa* serovar Patoc str. Patoc 1 (% identities, % similarities)
LIC_10321	696	dCACHE_1 - GAF - GGDEF	LA_0374 (99,99)	LBJ_2760 (79,87)	ND
LIC_11125	320	PAS - GGDEF	LA_2933 (100,100)	LBJ_2289 (67,81) with the gene with locus_tag LIC_11129	LEPBI_I3382 (29,45) with the gene with locus_tag LIC_11129
LIC_11126	311	PAS - GGDEF	LA_2932 (100,100)		
LIC_11127	311	PAS - GGDEF	LA_2931 (99,99)		
LIC_11128	308	PAS - GGDEF	LA_2930 (100,100)		
LIC_11129	317	PAS - GGDEF	LA_2929 (99,100)		
LIC_11130	315	PAS - GGDEF	LA_2927 (99,100)		
LIC_11131	359	PAS - GGDEF	LA_2926 (100,100)		
LIC_11167	384	GAF - GGDEF	LA_2877 (99,100)	LBJ_2031 (68,81)	ND
LIC_11247	718	GAF - GAF - GGDEF	LA_2772 (100,100)	LBJ_1748 (77,84)	LEPBI_I0811 (38, 62)
LIC_11300	409	GGDEF	LA_2704 (100,100)	LBJ_2009 (86,91)	LEPBI_I1800 (34,53)

LIC_11444	330	REC - GGDEF	LA_2528 (100,100)	LBJ_1672 (92,96)	LEPBI_p0053 (34,53)
LIC_11706	147	GGDEF	LA_2227 (99,100)	LBJ_1371 (85,93)	ND
LIC_12273	352	GAF - GGDEF	LA_1483 (100,100)	LBJ_0983 (94,99)	LEPBI_I1337 (67,82)
LIC_13137	327	GAF - GGDEF	LA_3929 (100,100)	LBJ_0386 (78,91)	ND
LIC_20181	524	CACHE1 - GGDEF	LB_237, LB_238 (57,57)	LBJ_4068 (75,85)	ND
LIC_20182	561	CACHE1 - GGDEF	LB_240 (100,100)	LBJ_4068 (53,69)	ND
LIC_12505	569	REC - GGDEF -EAL	LA_1185 (100,100)	LBJ_4069 (56,77)	LEPBI_I1882 (45,63)
LIC_13120	555	GGDEF - EAL	LA_3909 (99,100)	LBJ_2510 (86,92)	ND
LIC_20106	534	PAS - GGDEF - EAL	LB_133 (99,99)	LBJ_2510 (24,41)	LEPBI_I3429 (17,29)
LIC_20180	572	REC - GGDEF -EAL	LB_235 (100,100)	LBJ_4069 (90,95)	LEPBI_I1882 (43, 65)
LIC_20198	450	GGDEF - EAL	LB_261 (100,100)	LBJ_4114 (90,96)	LEPBI_I0054 (53,71)
LIC_10641	442	EAL - YkuI	ND	LBJ_2357 (88,91)	LEPBI_I1921 (50,65)
LIC_10996	297	EAL	LA_3104 (100,100)	LBJ_0354 (68,81)	ND
LIC_11203	404	REC -EAL	LA_2827 (100,100)	LBJ_1002 (88,93)	ND
LIC_11921	424	EAL - YkuI	LA_1983 (100,100)	LBJ_2357 (32,55)	ND
LIC_10122	433	DUF 3391 - HD-GYP	LA_0135 (100,100)	LBJ_01189 (86,93)	LEPBI_I1560 (35,58)
LIC_10138	489	DUF 3391 - HD-GYP	LA_0153 (100,100)	LBJ_0134 (92,97)	LEPBI_I1137 (41,63) with the gene with locus_tag LIC_10138
LIC_10139	503	DUF 3391 - HD-GYP	LA_0156 (100,100)	LBJ_0135 (89,95)	

LIC_11189	660	REC - GAF - HD-GYP	LA_2847 (100,100)	LBJ_0788 (86, 94)	LEPBI_I1194 (59,78)
LIC_11563	394	DUF 3391 - HD-GYP	LA_2383 (100,100)	LBJ_1428 (92,96)	LEPBI_I1668 (44,67)
LIC_10049	382	PilZN - GAZ - PIlZ	LA_0055 (100,100)	LBJ_0043 (91, 96)	LEPBI_I3237 (36,56)
LIC_10128	115	PilZ	LA_0142 (100,100)	LBJ_0125 (84,90)	ND
LIC_11447	167	PilZ	LA_2519 (100,100)	LBJ_1670 (77,81)	ND
LIC_11920	389	PilZN - GAZ - PIlZ	LA_1985 (100,100)	LBJ_1531 (88,94)	LEPBI_I1271 (46,68)
LIC_11993	101	PilZ	LA_1910 (99,100)	LBJ_1497 (89,96)	ND
LIC_12491	119	PilZ	LA_1204 (99,100)	LBJ_0892 (77,92)	LEPBI_I2971 (32,58)
LIC_12546	391	PilZN - GAZ - PIlZ	LA_1135 (100,100)	LBJ_2681 (80,91)	LEPBI_I2723 (36,62)
LIC_12723	428	PilZN - GAZ - PIlZ	LA_0920 (100,100)	LBJ_2209 (90,97)	LEPBI_I0868 (52,72)
LIC_12994	379	PilZN - GAZ - PIlZ	LA_0576 (100,100)	LBJ_0581 (76,87)	LEPBI_I2723 (39,66)
LIC_14002	357	PilZN - GAZ - PIlZ	LA_1489 (100,100)	LBJ_0988 (92,95)	LEPBI_I1342 (40,61)
LIC_20136	707	D1 - PilZ - D2	LB_167 (99,100)	*	ND
LIC_20173	463	PrsW - PilZ	LB_218 (99,99)	LBJ_4137 (85,91)	LEPBI_I1745 (29,48)
LIC_11571	557	MshEN - T2SSE	LA_2374 (100,100)	LBJ_1420 (94,97)	LEPBI_I1676 (72,88)

References

1. Altschul, S.F.; Gish, W.; Miller, W.; Myers, E.W.; Lipman, D.J. Basic Local Alignment Search Tool. *J. Mol. Biol.* **1990**, *215*, 403–410, doi:10.1016/S0022-2836(05)80360-2.
2. Stelitano, V.; Giardina, G.; Paiardini, A.; Castiglione, N.; Cutruzzolà, F.; Rinaldo, S. C-Di-GMP Hydrolysis by Pseudomonas Aeruginosa HD-GYP Phosphodiesterases: Analysis of the Reaction Mechanism and Novel Roles for pGpG. *PLoS One* **2013**, *8*, e74920, doi:10.1371/journal.pone.0074920.
3. Probst, A.J.; Ladd, B.; Jarett, J.K.; Geller-McGrath, D.E.; Sieber, C.M.K.; Emerson, J.B.; Anantharaman, K.; Thomas, B.C.; Malmstrom, R.R.; Stieglmeier, M.; et al. Differential Depth Distribution of Microbial Function and Putative Symbionts through Sediment-Hosted Aquifers in the Deep Terrestrial Subsurface. *Nat. Microbiol.* **2018**, *3*, 328–336, doi:10.1038/s41564-017-0098-y.
4. Ward, L.M.; Bertran, E.; Johnston, D.T. Genomic Sequence Analysis of Dissulfurirhabdus Thermomarina SH388 and Proposed Reassignment to Dissulfurirhabdaceae Fam. Nov. *Microb. Genom.* **2020**, *6*, doi:10.1099/mgen.0.000390.
5. Harf-Monteil, C.; Flèche, A.L.; Riegel, P.; Prévost, G.; Bermond, D.; Grimont, P.A.D.; Monteil, H. Aeromonas Simiae Sp. Nov., Isolated from Monkey Faeces. *Int. J. Syst. Evol. Microbiol.* **2004**, *54*, 481–485, doi:10.1099/ijs.0.02786-0.
6. Laskowski, R.A.; Jabłońska, J.; Pravda, L.; Vařeková, R.S.; Thornton, J.M. PDBsum: Structural Summaries of PDB Entries. *Protein Sci.* **2018**, *27*, 129–134, doi:10.1002/pro.3289.
7. Klein, B.J.; Bose, D.; Baker, K.J.; Yusoff, Z.M.; Zhang, X.; Murakami, K.S. RNA Polymerase and Transcription Elongation Factor Spt4/5 Complex Structure. *Proc. Natl. Acad. Sci. U. S. A.* **2011**, *108*, 546–550, doi:10.1073/pnas.1013828108.
8. Tallant, C.; Savitsky, P.; Moehlenbrink, J.; Chan, C.; Nunez-Alonso, G.; Newman, J.A.; von Delft, F.; Arrowsmith, C.H.; Edwards, A.M.; Bountra, C.; et al. Crystal Structure of Human TDRD1 Extended Tudor Domain in Complex with a Symmetrically Dimethylated E2F Peptide 2016.
9. Keidel, A.; Kögel, A.; Reichelt, P.; Kowalinski, E.; Schäfer, I.B.; Conti, E. Concerted Structural Rearrangements Enable RNA Channeling into the Cytoplasmic Ski238-Ski7-Exosome Assembly. *Mol. Cell* **2023**, *83*, 4093–4105.e7, doi:10.1016/j.molcel.2023.09.037.
10. Vanden Broeck, A.; Klinge, S. Principles of Human Pre-60S Biogenesis. *Science* **2023**, *381*, eadh3892, doi:10.1126/science.adh3892.
11. Mohanty, S.; Jobichen, C.; Chichili, V.P.R.; Velázquez-Campoy, A.; Low, B.C.; Hogue, C.W.V.; Sivaraman, J. Structural Basis for a Unique ATP Synthase Core Complex from Nanoarcheaum Equitans. *J. Biol. Chem.* **2015**, *290*, 27280–27296, doi:10.1074/jbc.M115.677492.
12. Bian, C.; Xu, C.; Ruan, J.; Lee, K.K.; Burke, T.L.; Tempel, W.; Barsyte, D.; Li, J.; Wu, M.; Zhou, B.O.; et al. Sgf29 Binds Histone H3K4me2/3 and Is Required

for SAGA Complex Recruitment and Histone H3 Acetylation. *EMBO J.* **2011**, *30*, 2829–2842, doi:10.1038/emboj.2011.193.
13. Das, D.; Kozbial, P.; Axelrod, H.L.; Miller, M.D.; McMullan, D.; Krishna, S.S.; Abdubek, P.; Acosta, C.; Astakhova, T.; Burra, P.; et al. Crystal Structure of a Novel Sm-like Protein of Putative Cyanophage Origin at 2.60 Å Resolution. *Proteins* **2009**, *75*, 296–307, doi:10.1002/prot.22360.
14. Tobiasson, V.; Berzina, I.; Amunts, A. Structure of a Mitochondrial Ribosome with Fragmented rRNA in Complex with Membrane-Targeting Elements. *Nat. Commun.* **2022**, *13*, 6132, doi:10.1038/s41467-022-33582-5.
15. Holm, L. Dali Server: Structural Unification of Protein Families. *Nucleic Acids Res.* **2022**, *50*, W210–W215, doi:10.1093/nar/gkac387.
16. Shim, S.R.; Kim, S.-J.; Lee, J.; Rücker, G. Network Meta-Analysis: Application and Practice Using R Software. *Epidemiol. Health* **2019**, *41*, e2019013, doi:10.4178/epih.e2019013.
17. Gu, Z.; Eils, R.; Schlesner, M. Complex Heatmaps Reveal Patterns and Correlations in Multidimensional Genomic Data. *Bioinformatics* **2016**, *32*, 2847–2849, doi:10.1093/bioinformatics/btw313.
18. Paradis, E.; Blomberg, S.; Bolker, B.; Brown, J.; Claramunt, S.; Claude, J.; Cuong, H.S.; Desper, R.; Didier, G.; Durand, B.; et al. Ape: Analyses of Phylogenetics and Evolution. *CRAN: Contributed Packages* 2002.
19. Bellini, D.; Caly, D.L.; McCarthy, Y.; Bumann, M.; An, S.-Q.; Dow, J.M.; Ryan, R.P.; Walsh, M.A. Crystal Structure of an HD-GYP Domain Cyclic-Di-GMP Phosphodiesterase Reveals an Enzyme with a Novel Trinuclear Catalytic Iron Centre: Structure of an HD-GYP Domain Phosphodiesterase. *Mol. Microbiol.* **2014**, *91*, 26–38, doi:10.1111/mmi.12447.
20. Meng, E.C.; Goddard, T.D.; Pettersen, E.F.; Couch, G.S.; Pearson, Z.J.; Morris, J.H.; Ferrin, T.E. UCSF ChimeraX: Tools for Structure Building and Analysis. *Protein Sci.* **2023**, *32*, e4792, doi:10.1002/pro.4792.
21. Barrio-Hernandez, I.; Yeo, J.; Jänes, J.; Mirdita, M.; Gilchrist, C.L.M.; Wein, T.; Varadi, M.; Velankar, S.; Beltrao, P.; Steinegger, M. Clustering Predicted Structures at the Scale of the Known Protein Universe. *Nature* **2023**, *622*, 637–645, doi:10.1038/s41586-023-06510-w.
22. Abramson, J.; Adler, J.; Dunger, J.; Evans, R.; Green, T.; Pritzel, A.; Ronneberger, O.; Willmore, L.; Ballard, A.J.; Bambrick, J.; et al. Accurate Structure Prediction of Biomolecular Interactions with AlphaFold 3. *Nature* **2024**, *630*, 493–500, doi:10.1038/s41586-024-07487-w.
23. Blum, M.; Andreeva, A.; Florentino, L.C.; Chuguransky, S.R.; Grego, T.; Hobbs, E.; Pinto, B.L.; Orr, A.; Paysan-Lafosse, T.; Ponamareva, I.; et al. InterPro: The Protein Sequence Classification Resource in 2025. *Nucleic Acids Res.* **2025**, *53*, D444–D456, doi:10.1093/nar/gkae1082.
24. Marchler-Bauer, A.; Zheng, C.; Chitsaz, F.; Derbyshire, M.K.; Geer, L.Y.; Geer, R.C.; Gonzales, N.R.; Gwadz, M.; Hurwitz, D.I.; Lanczycki, C.J.; et al. CDD: Conserved Domains and Protein Three-Dimensional Structure. *Nucleic Acids Res.* **2013**, *41*, D348–D352, doi:10.1093/nar/gks1243.

25. Waterhouse, A.M.; Procter, J.B.; Martin, D.M.A.; Clamp, M.; Barton, G.J. Jalview Version 2--a Multiple Sequence Alignment Editor and Analysis Workbench. *Bioinformatics* **2009**, *25*, 1189–1191, doi:10.1093/bioinformatics/btp033.
26. Schneider, T.D.; Stephens, R.M. Sequence Logos: A New Way to Display Consensus Sequences. *Nucleic Acids Res.* **1990**, *18*, 6097–6100, doi:10.1093/nar/18.20.6097.
27. Crooks, G.E.; Hon, G.; Chandonia, J.-M.; Brenner, S.E. WebLogo: A Sequence Logo Generator. *Genome Res.* **2004**, *14*, 1188–1190, doi:10.1101/gr.849004.
28. Sievers, F.; Wilm, A.; Dineen, D.; Gibson, T.J.; Karplus, K.; Li, W.; Lopez, R.; McWilliam, H.; Remmert, M.; Söding, J.; et al. Fast, Scalable Generation of High-Quality Protein Multiple Sequence Alignments Using Clustal Omega. *Mol. Syst. Biol.* **2011**, *7*, 539, doi:10.1038/msb.2011.75.
29. Galperin, M.Y.; Chou, S.-H. Sequence Conservation, Domain Architectures, and Phylogenetic Distribution of the HD-GYP Type c-Di-GMP Phosphodiesterases. *J. Bacteriol.* **2022**, *204*, e0056121, doi:10.1128/jb.00561-21.
30. Paysan-Lafosse, T.; Andreeva, A.; Blum, M.; Chuguransky, S.R.; Grego, T.; Pinto, B.L.; Salazar, G.A.; Bileschi, M.L.; Llinares-López, F.; Meng-Papaxanthos, L.; et al. The Pfam Protein Families Database: Embracing AI/ML. *Nucleic Acids Res.* **2025**, *53*, D523–D534, doi:10.1093/nar/gkae997.
31. Tamura, K.; Stecher, G.; Kumar, S. MEGA11: Molecular Evolutionary Genetics Analysis Version 11. *Mol. Biol. Evol.* **2021**, *38*, 3022–3027, doi:10.1093/molbev/msab120.
32. van Kempen, M.; Kim, S.S.; Tumescheit, C.; Mirdita, M.; Lee, J.; Gilchrist, C.L.M.; Söding, J.; Steinegger, M. Fast and Accurate Protein Structure Search with Foldseek. *Nat. Biotechnol.* **2024**, *42*, 243–246, doi:10.1038/s41587-023-01773-0.
33. Chen, Y.; Tsai, B.; Li, N.; Gao, N. Structural Remodeling of Ribosome Associated Hsp40-Hsp70 Chaperones during Co-Translational Folding. *Nat. Commun.* **2022**, *13*, 3410, doi:10.1038/s41467-022-31127-4.
34. Pichlo, C.; Juetten, L.; Wojtalla, F.; Schacherl, M.; Diaz, D.; Baumann, U. Molecular Determinants of the Mechanism and Substrate Specificity of Clostridium Difficile Proline-Proline Endopeptidase-1. *J. Biol. Chem.* **2019**, *294*, 11525–11535, doi:10.1074/jbc.RA119.009029.
35. Schacherl, M.; Pichlo, C.; Neundorf, I.; Baumann, U. Structural Basis of Proline-Proline Peptide Bond Specificity of the Metalloprotease Zmp1 Implicated in Motility of Clostridium Difficile. *Structure* **2015**, *23*, 1632–1642, doi:10.1016/j.str.2015.06.018.
36. Pannifer, A.D.; Wong, T.Y.; Schwarzenbacher, R.; Renatus, M.; Petosa, C.; Bienkowska, J.; Lacy, D.B.; Collier, R.J.; Park, S.; Leppla, S.H.; et al. Crystal Structure of the Anthrax Lethal Factor. *Nature* **2001**, *414*, 229–233, doi:10.1038/n35101998.
37. Visschedyk, D.; Rochon, A.; Tempel, W.; Dimov, S.; Park, H.-W.; Merrill, A.R. Certhrax Toxin, an Anthrax-Related ADP-Ribosyltransferase from Bacillus

Cereus. *J. Biol. Chem.* **2012**, *287*, 41089–41102, doi:10.1074/jbc.M112.412809.
38. Scheithauer, L.; Thiem, S.; Schmelz, S.; Dellmann, A.; Büssow, K.; Brouwer, R.M.H.J.; Ünal, C.M.; Blankenfeldt, W.; Steinert, M. Zinc Metalloprotease ProA of Legionella Pneumophila Increases Alveolar Septal Thickness in Human Lung Tissue Explants by Collagen IV Degradation. *Cell. Microbiol.* **2021**, *23*, e13313, doi:10.1111/cmi.13313.
39. Shen, Y.; Zhukovskaya, N.L.; Guo, Q.; Florián, J.; Tang, W.-J. Calcium-Independent Calmodulin Binding and Two-Metal-Ion Catalytic Mechanism of Anthrax Edema Factor. *EMBO J.* **2005**, *24*, 929–941, doi:10.1038/sj.emboj.7600574.
40. Papadopoulos, J.S.; Agarwala, R. COBALT: Constraint-Based Alignment Tool for Multiple Protein Sequences. *Bioinformatics* **2007**, *23*, 1073–1079, doi:10.1093/bioinformatics/btm076.
41. Pei, J.; Mitchell, D.A.; Dixon, J.E.; Grishin, N.V. Expansion of Type II CAAX Proteases Reveals Evolutionary Origin of γ-Secretase Subunit APH-1. *J. Mol. Biol.* **2011**, *410*, 18–26, doi:10.1016/j.jmb.2011.04.066.
42. Ellermeier, C.D.; Losick, R. Evidence for a Novel Protease Governing Regulated Intramembrane Proteolysis and Resistance to Antimicrobial Peptides in Bacillus Subtilis. *Genes Dev.* **2006**, *20*, 1911–1922, doi:10.1101/gad.1440606.
43. Huerta-Cepas, J.; Serra, F.; Bork, P. ETE 3: Reconstruction, Analysis, and Visualization of Phylogenomic Data. *Mol Biol Evol* **2016**, *33*, 1635–1638, doi:10.1093/molbev/msw046.
44. UniProt Consortium UniProt: The Universal Protein Knowledgebase in 2025. *Nucleic Acids Res.* **2025**, *53*, D609–D617, doi:10.1093/nar/gkae1010.
45. Hutchin, A.; Cordery, C.; Walsh, M.A.; Webb, J.S.; Tews, I. Phylogenetic Analysis with Prediction of Cofactor or Ligand Binding for Pseudomonas Aeruginosa PAS and Cache Domains. *Microbiol. Spectr.* **2021**, *9*, e0102621, doi:10.1128/spectrum.01026-21.
46. Brewster, J.L.; McKellar, J.L.O.; Finn, T.J.; Newman, J.; Peat, T.S.; Gerth, M.L. Structural Basis for Ligand Recognition by a Cache Chemosensory Domain That Mediates Carboxylate Sensing in Pseudomonas Syringae. *Sci. Rep.* **2016**, *6*, doi:10.1038/srep35198.
47. Upadhyay, A.A.; Fleetwood, A.D.; Adebali, O.; Finn, R.D.; Zhulin, I.B. Cache Domains That Are Homologous To, but Different from PAS Domains Comprise the Largest Superfamily of Extracellular Sensors in Prokaryotes. *PLoS Comput. Biol.* **2016**, *12*, e1004862, doi:10.1371/journal.pcbi.1004862.
48. Randall, T.E.; Eckartt, K.; Kakumanu, S.; Price-Whelan, A.; Dietrich, L.E.P.; Harrison, J.J. Sensory Perception in Bacterial Cyclic Diguanylate Signal Transduction. *J. Bacteriol.* **2022**, *204*, e0043321, doi:10.1128/JB.00433-21.
49. da Costa Vasconcelos, F.N.; Maciel, N.K.; Favaro, D.C.; de Oliveira, L.C.; Barbosa, A.S.; Salinas, R.K.; de Souza, R.F.; Farah, C.S.; Guzzo, C.R. Structural and Enzymatic Characterization of a cAMP-Dependent Diguanylate Cyclase from Pathogenic Leptospira Species. *J. Mol. Biol.* **2017**, *429*, 2337–2352, doi:10.1016/j.jmb.2017.06.002.

50. Heikaus, C.C.; Pandit, J.; Klevit, R.E. Cyclic Nucleotide Binding GAF Domains from Phosphodiesterases: Structural and Mechanistic Insights. *Structure* **2009**, *17*, 1551–1557, doi:10.1016/j.str.2009.07.019.
51. Stuffle, E.C.; Johnson, M.S.; Watts, K.J. PAS Domains in Bacterial Signal Transduction. *Curr. Opin. Microbiol.* **2021**, *61*, 8–15, doi:10.1016/j.mib.2021.01.004.
52. Taylor, B.L.; Zhulin, I.B. PAS Domains: Internal Sensors of Oxygen, Redox Potential, and Light. *Microbiol. Mol. Biol. Rev.* **1999**, *63*, 479–506, doi:10.1128/MMBR.63.2.479-506.1999.
53. Heintz, U.; Meinhart, A.; Winkler, A. Multi-PAS Domain-Mediated Protein Oligomerization of PpsR from Rhodobacter Sphaeroides. *Acta Crystallogr. D Biol. Crystallogr.* **2014**, *70*, 863–876, doi:10.1107/S1399004713033634.
54. Hefti, M.H.; Françoijs, K.-J.; de Vries, S.C.; Dixon, R.; Vervoort, J. The PAS Fold. A Redefinition of the PAS Domain Based upon Structural Prediction. *Eur. J. Biochem.* **2004**, *271*, 1198–1208, doi:10.1111/j.1432-1033.2004.04023.x.
55. Galperin, M.Y. Diversity of Structure and Function of Response Regulator Output Domains. *Curr. Opin. Microbiol.* **2010**, *13*, 150–159, doi:10.1016/j.mib.2010.01.005.
56. Bourret, R.B. Receiver Domain Structure and Function in Response Regulator Proteins. *Curr. Opin. Microbiol.* **2010**, *13*, 142–149, doi:10.1016/j.mib.2010.01.015.
57. Pao, G.M.; Saier, M.H., Jr Response Regulators of Bacterial Signal Transduction Systems: Selective Domain Shuffling during Evolution. *J. Mol. Evol.* **1995**, *40*, 136–154, doi:10.1007/bf00167109.
58. Minasov, G.; Padavattan, S.; Shuvalova, L.; Brunzelle, J.S.; Miller, D.J.; Baslé, A.; Massa, C.; Collart, F.R.; Schirmer, T.; Anderson, W.F. Crystal Structures of YkuI and Its Complex with Second Messenger Cyclic Di-GMP Suggest Catalytic Mechanism of Phosphodiester Bond Cleavage by EAL Domains. *J. Biol. Chem.* **2009**, *284*, 13174–13184, doi:10.1074/jbc.M808221200.
59. Visnardi, A.B.; Ribeiro, R.A.; de Souza, A.S.; Churasacari Vinces, T.G.; Llontop, E.E.; de Almeida Ferrari, A.S.; França Henrique, P.A.; Valdivieso, D.; Sánchez-Limache, D.E.; Silva, G.R.; et al. Insertion of a Divergent GAF-like Domain Defines a Novel Family of YcgR Homologues That Bind c-Di-GMP in Leptospirales. *ACS Omega* **2025**, *10*, 3988–4006, doi:10.1021/acsomega.4c09917.
60. Heinrich, J.; Hein, K.; Wiegert, T. Two Proteolytic Modules Are Involved in Regulated Intramembrane Proteolysis of Bacillus Subtilis RsiW. *Mol. Microbiol.* **2009**, *74*, 1412–1426, doi:10.1111/j.1365-2958.2009.06940.x.
61. Cianciotto, N.P.; White, R.C. Expanding Role of Type II Secretion in Bacterial Pathogenesis and beyond. *Infect. Immun.* **2017**, *85*, doi:10.1128/IAI.00014-17.
62. Desvaux, M.; Parham, N.J.; Scott-Tucker, A.; Henderson, I.R. The General Secretory Pathway: A General Misnomer? *Trends Microbiol.* **2004**, *12*, 306–309, doi:10.1016/j.tim.2004.05.002.
63. Turner, L.R.; Lara, J.C.; Nunn, D.N.; Lory, S. Mutations in the Consensus ATP-

Binding Sites of XcpR and PilB Eliminate Extracellular Protein Secretion and Pilus Biogenesis in Pseudomonas Aeruginosa. *J. Bacteriol.* **1993**, *175*, 4962–4969, doi:10.1128/jb.175.16.4962-4969.1993.
64. Römling, U.; Liang, Z.-X.; Dow, J.M. Progress in Understanding the Molecular Basis Underlying Functional Diversification of Cyclic Dinucleotide Turnover Proteins. *J. Bacteriol.* **2017**, *199*, doi:10.1128/JB.00790-16.
65. Sultan, S.Z.; Pitzer, J.E.; Boquoi, T.; Hobbs, G.; Miller, M.R.; Motaleb, M.A. Analysis of the HD-GYP Domain Cyclic Dimeric GMP Phosphodiesterase Reveals a Role in Motility and the Enzootic Life Cycle of Borrelia Burgdorferi. *Infect. Immun.* **2011**, *79*, 3273–3283, doi:10.1128/IAI.05153-11.
66. Cutruzzolà, F.; Paiardini, A.; Scribani Rossi, C.; Spizzichino, S.; Paone, A.; Giardina, G.; Rinaldo, S. A Conserved Scaffold with Heterogeneous Metal Ion Binding Site: The Multifaceted Example of HD-GYP Proteins. *Coord. Chem. Rev.* **2022**, *450*, 214228, doi:10.1016/j.ccr.2021.214228.
67. Correction for Ryan et Al., Cell-Cell Signal-Dependent Dynamic Interactions between HD-GYP and GGDEF Domain Proteins Mediate Virulence in Xanthomonas Campestris. *Proc. Natl. Acad. Sci. U. S. A.* **2017**, *114*, E1303, doi:10.1073/pnas.1700657114.
68. Ren, S.-X.; Fu, G.; Jiang, X.-G.; Zeng, R.; Miao, Y.-G.; Xu, H.; Zhang, Y.-X.; Xiong, H.; Lu, G.; Lu, L.-F.; et al. Unique Physiological and Pathogenic Features of Leptospira Interrogans Revealed by Whole-Genome Sequencing. *Nature* **2003**, *422*, 888–893, doi:10.1038/nature01597.
69. Bulach, D.M.; Zuerner, R.L.; Wilson, P.; Seemann, T.; McGrath, A.; Cullen, P.A.; Davis, J.; Johnson, M.; Kuczek, E.; Alt, D.P.; et al. Genome Reduction in Leptospira Borgpetersenii Reflects Limited Transmission Potential. *Proc. Natl. Acad. Sci. U. S. A.* **2006**, *103*, 14560–14565, doi:10.1073/pnas.0603979103.
70. Picardeau, M.; Bulach, D.M.; Bouchier, C.; Zuerner, R.L.; Zidane, N.; Wilson, P.J.; Creno, S.; Kuczek, E.S.; Bommezzadri, S.; Davis, J.C.; et al. Genome Sequence of the Saprophyte Leptospira Biflexa Provides Insights into the Evolution of Leptospira and the Pathogenesis of Leptospirosis. *PLoS One* **2008**, *3*, e1607, doi:10.1371/journal.pone.0001607.
