# Supplementary material for: Discovery of Novel c-di-GMP-Related Genes in Leptospira interrogans
Source: Pathogens. 2026 Jan 30;15(2):151. doi: 10.3390/pathogens15020151 (PMC12943098; doi:10.3390/pathogens15020151)

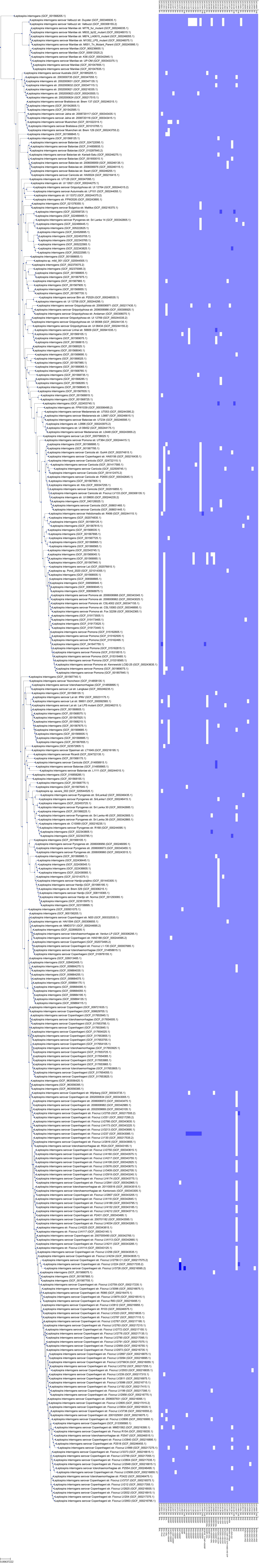

Supplement: Supplementary file 1 [file Figure S19.pdf › Figure S19.pdf]
